# Duckweeds: Model organisms for research on plant sterols and steroids

**DOI:** 10.1111/plb.70095

**Published:** 2025-08-25

**Authors:** J. Klein, K.‐J. Appenroth, K. S. Sree

**Affiliations:** ^1^ Department of Plant Physiology, Matthias‐Schleiden‐Institute for Genetics, Bioinformatics and Molecular Botany Friedrich Schiller University Jena Jena Germany; ^2^ School of Biotechnology, Institute of Science Banaras Hindu University Varanasi India

**Keywords:** Androgens, brassinosteroids, phytosterols, progestogens, specialized metabolism, *Spirodela polyrhiza*

## Abstract

More than two centuries since the birth of the botanist Matthias Jacob Schleiden, who first described many duckweed (Lemnaceae) species, interest in these small aquatic monocots is still alive. Lemnaceae have high biomass production capacity and can be used as animal feed and in human nutrition. Efficient transformation protocols and available genome data for several Lemnaceae species make them an ideal model system for research into biosynthesis and physiology of sterols and/or steroids in plants, especially monocots. Here we emphasize how studies using duckweed species can address current problems in plant physiology, with a strong focus on sterol and steroid biology in monocots. Further, we discuss how this knowledge can be translated to solve agricultural and industrial problems.

## Graphical abstract

Duckweeds (Lemnaceae), the fastest‐growing angiosperms and promising vegan protein source, offer a powerful model to address key questions in plant physiology. This article highlights their potential for dissecting the biosynthesis and function of phytosterols, sterol conjugates, brassinosteroids, and specialized steroidal metabolites, such as cardenolides.

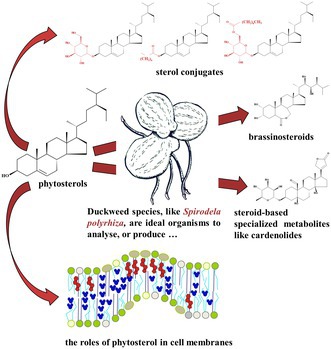

## INTRODUCTION

**Fig. 1:**
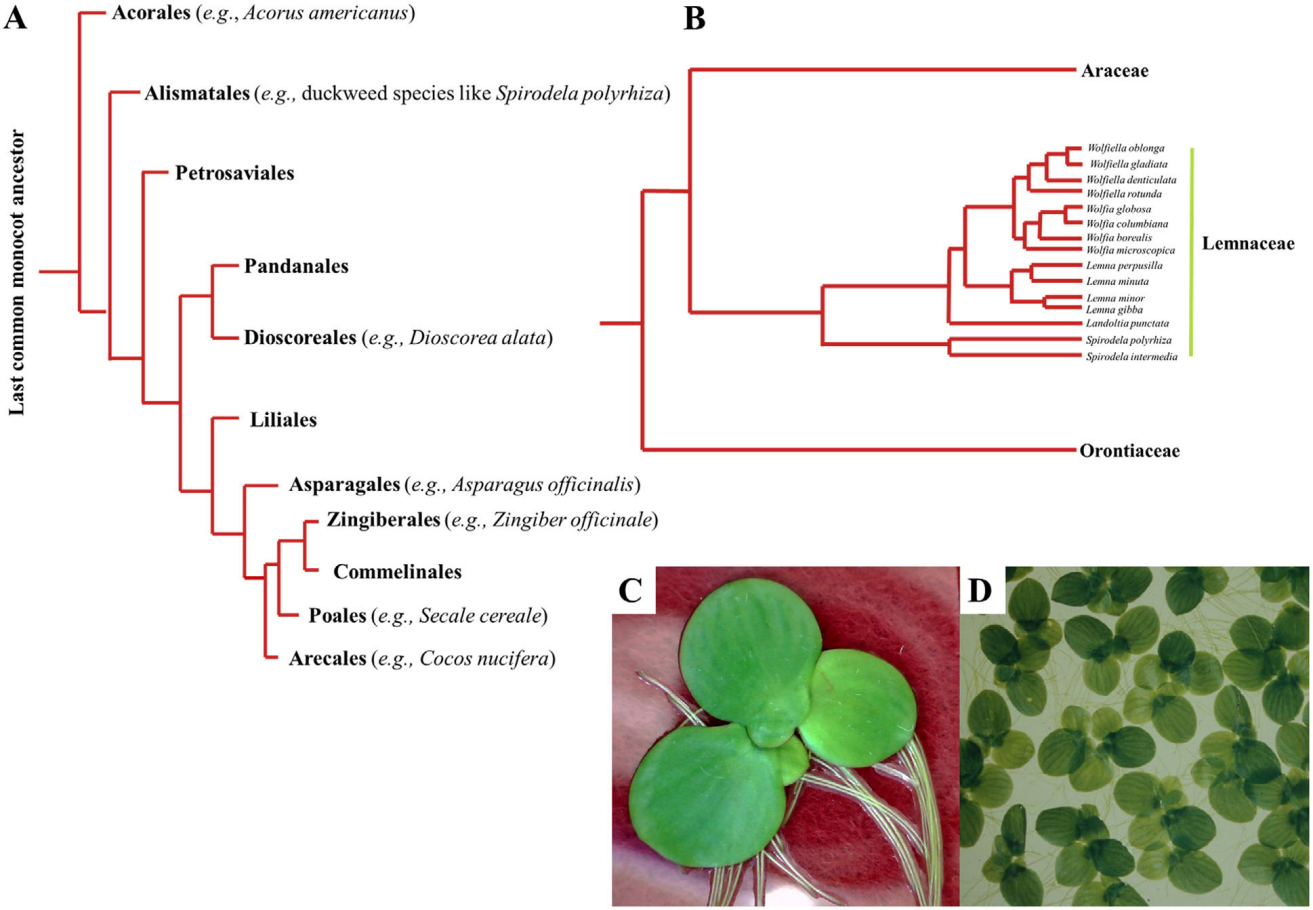
*Spirodela polyrhiza* as monocot species. (A) All orders of monocots (Arecales, Poales, Commelinales, Zingiberales, Asparagales, Liliales, Dioscoreales, Pandanales, Petrosaviales, Alismatales, and Acorales) have one common ancestor. *S. polyrhiza* is a member of Lemnaceae, order Alismatales. *S. polyrhiza* is a secondary aqueous monocot easily cultivated in liquid culture. (B) Lemnaceae are sister family of Araceae and Orontiaceae within the Alismatales (Phylogeny modified Tippery *et al*. 2021). A single plant of *S. polyrhiza* clone 9509 (C) and axenic population of *S. polyrhiza* (D). Both photographs were taken with material from Friedrich Schiller University stock, cultivated in sugar‐free liquid medium.

Duckweeds are in the Lemnaceae, order Alismatales (Fig. 1), with 35 species and two interspecific hybrids (nothotaxon) (Appenroth *et al*. 2018). In contrast to the definition of the Angiosperm Phylogeny Group III (APG, 1998), which categorizes Lemnaceae as a subfamily (Lemnoideae) of the Araceae, most researchers regard duckweeds as an independent family (Lemnaceae; Tippery *et al*. 2021). This aligns with general taxonomic principles, as noted by Bog *et al*. (2020) and Tippery & Les (2020).

The Lemnaceae contains five genera: *Spirodela, Landoltia, Lemna*, *Wolffiella*, and *Wolffia*. The duckweed stock collection at the Friedrich Schiller University of Jena, Germany, contains 500 duckweed clones, comprising all known duckweed species. Waterlentils, as they are also called, are the smallest (Landolt 1986; Ziegler *et al*. 2023) and most rapidly growing angiosperm (Ziegler *et al*. 2015), with doubling times from 1.24 to 4.54 days (Sree *et al*. 2015; Ziegler *et al*. 2015). Estimated yield for duckweed cultivation can compete with land‐based crop plants (Ziegler *et al*. 2015) and they are used as food in several Asian countries. Duckweeds comprise 20%–30% protein, 10%–20% starch, 1%–5%, and ~25% fibre per unit dry weight, make them a source of plant‐based protein (Appenroth *et al*. 2017, 2018). Duckweed associated bacteria produce vitamin B12, an important nutrient in plant‐based diets (Acosta *et al*. 2024). Therefore, duckweeds deserve our attention (Acosta *et al*. 2021). High growth rates and ease of cultivation (cf. Appenroth *et al*. 2018) make them interesting model organisms for use in controlled set‐ups and *in vitro* culture (Romano *et al*. 2022).

### Advantages of working with duckweeds

Monocotyledon (Fig. 1) sister groups are the Ceratophyllales and Eudicotyledons, while dicotyledons are divided into Magnoliopsida and Rosopsida. The evolutionary distance between the monocot *Secale cereale* (Poales) and *Spirodela polyrhiza* (Alismatales, duckweed) is 1.49 compared to that between the edible dicot *Daucus carota* and the secondary aquatic dicot *Nymphaea colorata*, which is 3.11, based on the coding sequence of DET2 calculated with Evolutionary Distance Calculator (http://www.nccbiology.com/support/242/[29 February 2024]) using the Jukes‐Cantor distance metric, Phytozome 13 (https://phytozome‐next.jgi.doe.gov/[29 February 2024]; Goodstein *et al*. 2012 and EnsemblePlants https://plants.ensembl.org/Secale_cereale/Tools/Blast?db=core [29 February 2024]; Harrison *et al*. 2024; Supporting Information S1). This close relationship within the monocots simplifies transfer of knowledge from one monocotyledon species to others.

Duckweeds are characterized by the alteration, simplification, or loss of numerous morphological and anatomical features. There are continuous increases in DNA content in the order *Spirodela*, *Landoltia*, *Lemna*, *Wolffiella*, and *Wolffia*, which parallel successive reduction in morphological complexity (Wang *et al*. 2011; Hoang *et al*. 2018). Nevertheless, some duckweed species have small genomes, and most have diploid genomes of 40 chromosomes (Hoang *et al*. 2022). Small genomes simplify generation of draft genomes (genomes of all five Lemnaceae genera are available; Wang et al. 2014; van Hoeck et al. 2015; Cao et al. 2020; Buendía‐Ávila & Marí‐Ordóñez 2024), transcriptome data (e.g., *L. punctata* under nutrient deficiency; Tao *et al*. 2013), and protein coding sequences (e.g., *S. polyrhiza* using Phytozome 13; Goodstein *et al*. 2012; Pakdee *et al*. 2022).

Unfortunately, the transformation of monocotyledonous crop species is complicated, time‐consuming and not very efficient. For genetic transformation of *Secale cereale*, a biolistic protocol (Popelka & Altpeter 2003a) or an *Agrobacterium tumefaciens*‐based protocol can be used (Popelka & Altpeter 2003b). In both strategies immature embryos are prepared from immature caryopses (Popelka & Altpeter 2003a, 2003b). Moreover, transformation efficiency is limited and depends strongly on the cultivar used. For *S. cereale*, an inbred line is most efficient (Popelka & Altpeter 2003a, 2003b), while for *Asparagus officinalis* (Chen *et al*. 2019) or *Elaeis guineensis* (Izawati *et al*. 2012), a preparation of embryos or cell suspension cultures (*Dioscorea alata*; Tör *et al*. 1993) is necessary, and for other species, protocols based on biolistic strategies are available (e.g., *Zingiber officinalis*) or no transformation protocol is required (e.g., *Cocos nucifera*). In this context, advances in genetic transformation of duckweed species offers several advantages. For an overview of transformation efficiencies of different monocot orders and the explant used see Table 1.

1. In duckweeds, fronds or even whole plants can be used for transformation and regeneration (Yamamoto *et al*. 2001; Vunsh *et al*. 2007; Khvatkov *et al*. 2015; Yang *et al*. 2018; Wang *et al*. 2021). Because of their rapid growth rate duckweed explants can be prepared in a very time‐efficient manner.
2. Rapid growth allows rapid regeneration of transgenic plants. *Lemna minor* T‐DNA plants can be regenerated in 8–12 weeks, which is less than half the time required for other monocots. For example, production of transgenic *S. cereale* needs 5 days of callus induction, 2 weeks of callus culture, and 5 weeks of callus regeneration, before plants can be adapted to the greenhouse and tested for transgenic nature (Popelka & Altpeter 2003a, 2003b).
3. Viral promoters such as the CamV 35S promoter can be used for overexpression of transgenes in duckweeds (Yang *et al*. 2018). These promoters are used in many constructs for transformation of dicots (e.g., *Arabidopsis thaliana*) and Lemnaceae.
4. Duckweeds also contain comparably high amounts of protein and therefore transgenic duckweed species produce high amounts of transgenic proteins (Vunsh *et al*. 2007; Firsov *et al*. 2018) because of their rapid growth rate.
5. In nature and in *in vitro* cultures, duckweed proliferation is mainly vegetative, allowing maintenance of transgenic cultures. Use of T1 generations allows rapid follow‐up experiments after transformation. The CRISPR/Cas method is available for duckweeds (Liu *et al*. 2019).

**Table 1:** Efficiencies of genetic transformation of monocot species.

species	*L. minor* (a) alismatales	*S. cereale* (b) poales	*A. officinalis* (c) asparagales	*E. guineensis* (d) arecales	*D. alata* (e) dioscoreales
Explant type	Fronds (leaves)	Embryos	Embryos	Embryos	Suspension culture
Stable	n.d.	Max. 4%	21%	n.d.	n.d.

The table depicts usable explant types and efficiencies of stable transformations for monocots from five of the 11 monocot orders. (a) Yang *et al*. (2018); (b) Popelka & Altpeter (2003b); (c) Chen *et al*. (2019); (d) Izawati *et al*. (2012); (e) Tör *et al*. (1993).

n.d., not determined.

In summary, duckweed species can be used to address complicated questions, such as sterol and steroid biology, and to transfer the obtained knowledge to monocot crop species like asparagus, maize, rice, wheat, barley, rye, banana, or coconut.

### Sterol and steroid biology of duckweeds

#### Sterol chemistry

**Fig. 2:**
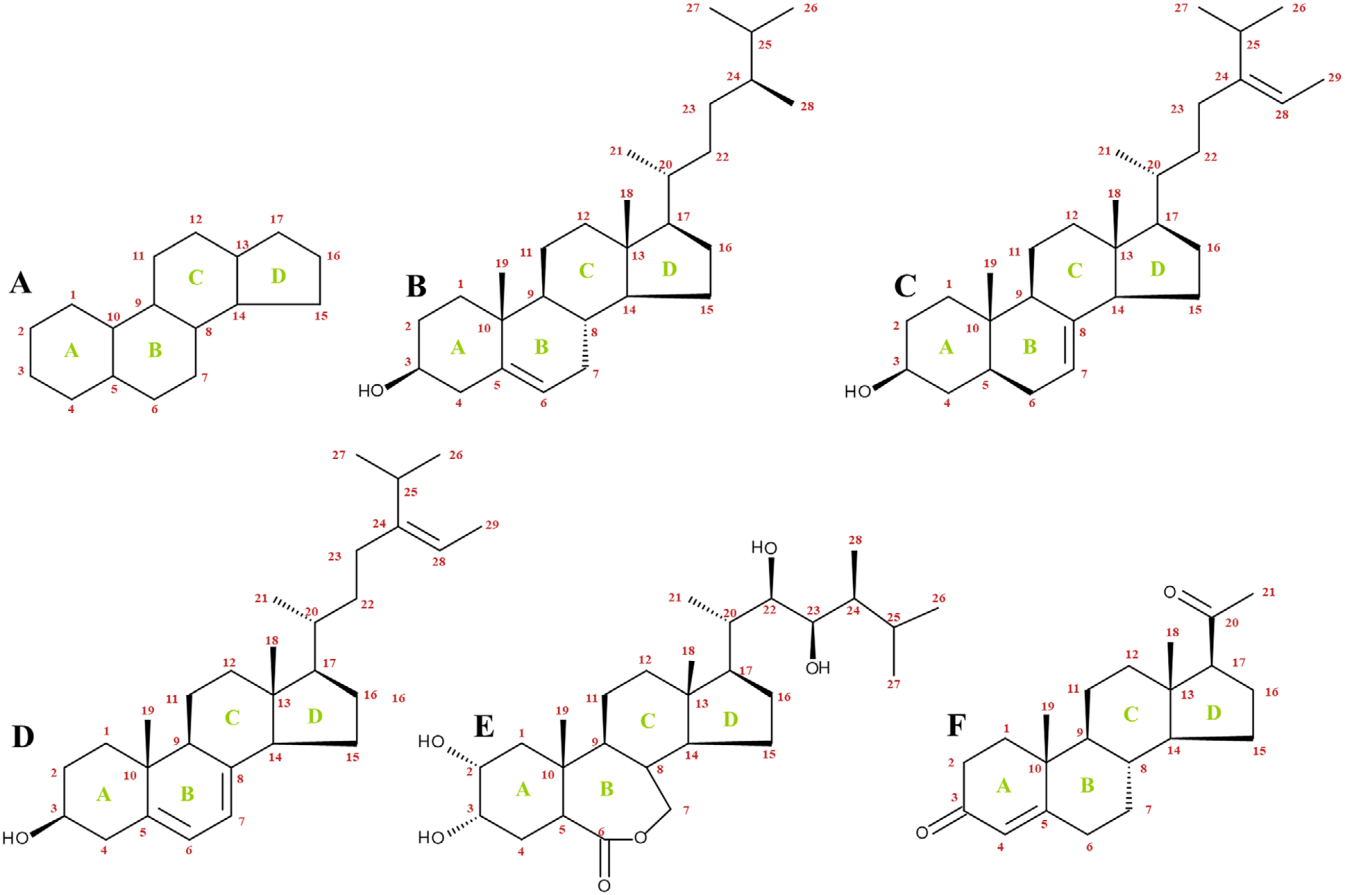
Structure and numbering of carbon atoms in the sterane skeleton of steroids. Steroids are tetracyclic triterpenoids with a sterane skeleton. This figure depicts the sterane skeleton (A) of steroids. Campesterol (B), a Δ^5^‐phytosterol, is characterized by the typical hydroxyl group at C3, a C_9_‐side chain at C17, and methyl groups at C10, C13, C20, and C24, while a double bond connects C5 and C6. Apart from Δ^5^‐phytosterols, plants contain Δ^7^‐phytosterols like Δ^7^‐avenosterol (C) which is characterized by a hydroxyl group at C3, a double bond between C7 and C8, methyl groups at C10 and C13, while a propylene group is added to C24. A plant Δ^5,7^‐sterol is Δ^5,7^‐avenosterol (D). Shown is the structure of Δ^7^‐avenosterol with an additional double bond between C5 and C6. Brassinolide (E), a brassinosteroid, can be described as campesterol with additional hydroxyl groups at C2, C22, and C23. In contrast to the double bond between C5 and C6 in campesterol, brassinolide contains an oxygen atom between C6 and C7. Progesterone (F), a C_21_ steroid, is the precursor of pharmaceutically important plant metabolites, the cardenolides (Klein 2024). In progesterone the sterane skeleton is substituted with a methyl group at C10 and C13, and an oxo moiety on C3, while C4 and C5 are connected by a double bond. A propan‐2‐ol group is added to C17. Carbon numbering (red) and annotation of the rings (green) in the molecules follows IUPAC.

Sterols and steroids are tetracyclic triterpenoids based on a sterane skeleton (Fig. 2). Phytosterols are divided into Δ5‐steroids (double bond between C5 and C6) and plant stanols or 5α‐sterols with a saturated sterol ring (MacKay & Jones 2011). Sterols (e.g., campesterol) are squalene‐derived steroid alcohols (Fig. 2) with a basic structure of 5α‐cholestan‐3β‐ol, comprising a tetracyclic cyclopenta[a]phenanthrene ring and an extended, flexible side chain at C17 carbon. These structures have *trans*‐ring junctures in the four rings (A, B, C, D), creating a flat α‐system. The side chain, together with two methyl groups (C18, C19), is at an angle to the ring structure and positioned above the plane, adopting a β‐stereochemistry. Additionally, because of a preference for the 20*R* conformation in the side chain, sterols adopt a planar configuration at both top and bottom of the molecule. This configuration facilitates multiple hydrophobic interactions between the rigid sterol nucleus and the membrane matrix. Generally, the hydroxyl group at C3 also has β‐stereochemistry. Plant sterols can be categorized based on their structure and biosynthesis into 4‐desmethyl sterols, 4α‐monomethyl sterols, and 4,4‐dimethyl sterols. The 4,4‐dimethyl sterols and 4α‐methyl sterols serve as precursors for plant sterols and are present at lower levels compared to the final products, which are 4‐desmethyl sterols. The 4‐desmethyl sterols further fall into subcategories: Δ^5^‐sterols (e.g., campesterol; Fig. 2B), Δ^7^‐sterols (e.g., Δ^7^‐avenosterol; Fig. 2C), and Δ^5,7^‐sterols (e.g., Δ^5,7^‐avenosterol; Fig. 2D), based on position and number of double bonds in the B ring (Silvestro *et al*. 2013). Most plant sterols, such as campesterol and sitosterol, have a Δ^5^ bond and an additional one‐carbon or two‐carbon substituent in the side chain at C24. The phytosterol campesterol (Fig. 2B) contains a double bond between C5 and C6 (Δ^5^ bond), as well as a methyl group (one‐carbon substituent) at C24 (phytosterol chemistry is reviewed in: Piironen *et al*. 2000).

**Fig. 3:**
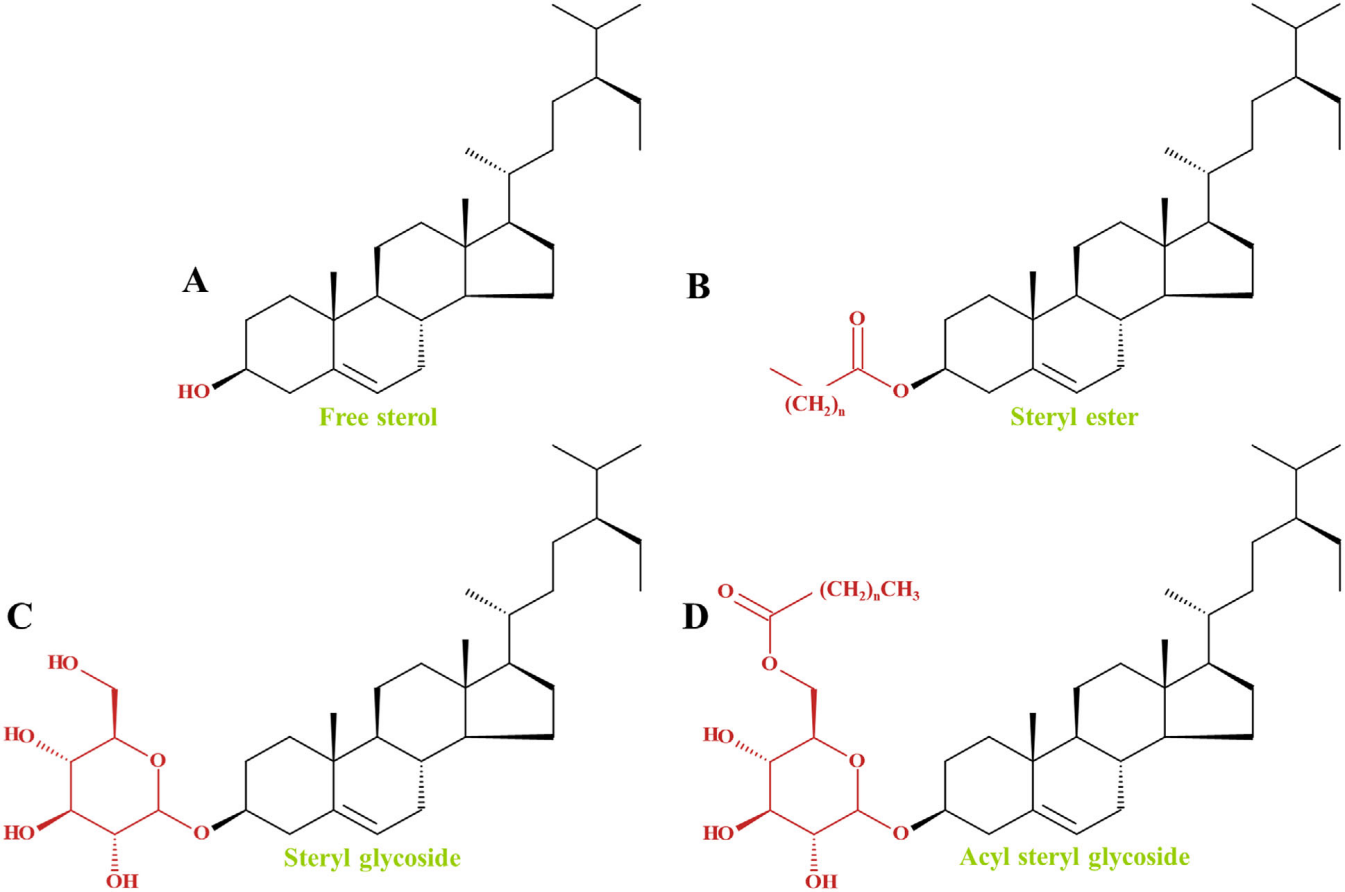
Sterol conjugates of β‐sitosterol. Major sterol conjugates derived from the Δ^5^‐phytosterol β‐sitosterol. The free sterol (A) contains a hydroxyl group at the C3 position (highlighted in red), which serves as the functional site for conjugation. In steryl esters (B), a fatty acid (red) is linked to C3 via an ester bond. In steryl glycosides (C), a monosaccharide (red) is attached through a β‐glycosidic bond to C3 of the sterol. Acyl steryl glycosides (D) represent a further modification, where the glycosidic moiety is acylated with a fatty acid at the C6 position of the sugar residue.

Apart from free phytosterols, plants contain sterol conjugates. These include steryl esters (SEs), steryl glycosides (SGs) and acyl steryl glycosides (ASGs; cf. Ferrer *et al*. 2017; Zhang, Li, *et al*. 2020; Zhang, Lin, *et al*. 2020; Rogowska & Szakiel 2020), which vary in residues bound to the hydroxyl group at C3, such as a fatty acid bound as an ester (SEs), or a sugar bound by a β‐glycosidic bond (SGs). In ASGs the sugar residues of an SG have a fatty acid, bound by an ester bond at C6 of the sugar moiety (Ferrer *et al*. 2017; Rogowska & Szakiel 2020). Sterol conjugates based on the Δ^5^‐sterol β‐sitosterol are shown in Fig. 3.

#### Phytosterol physiology

**Fig. 4:**
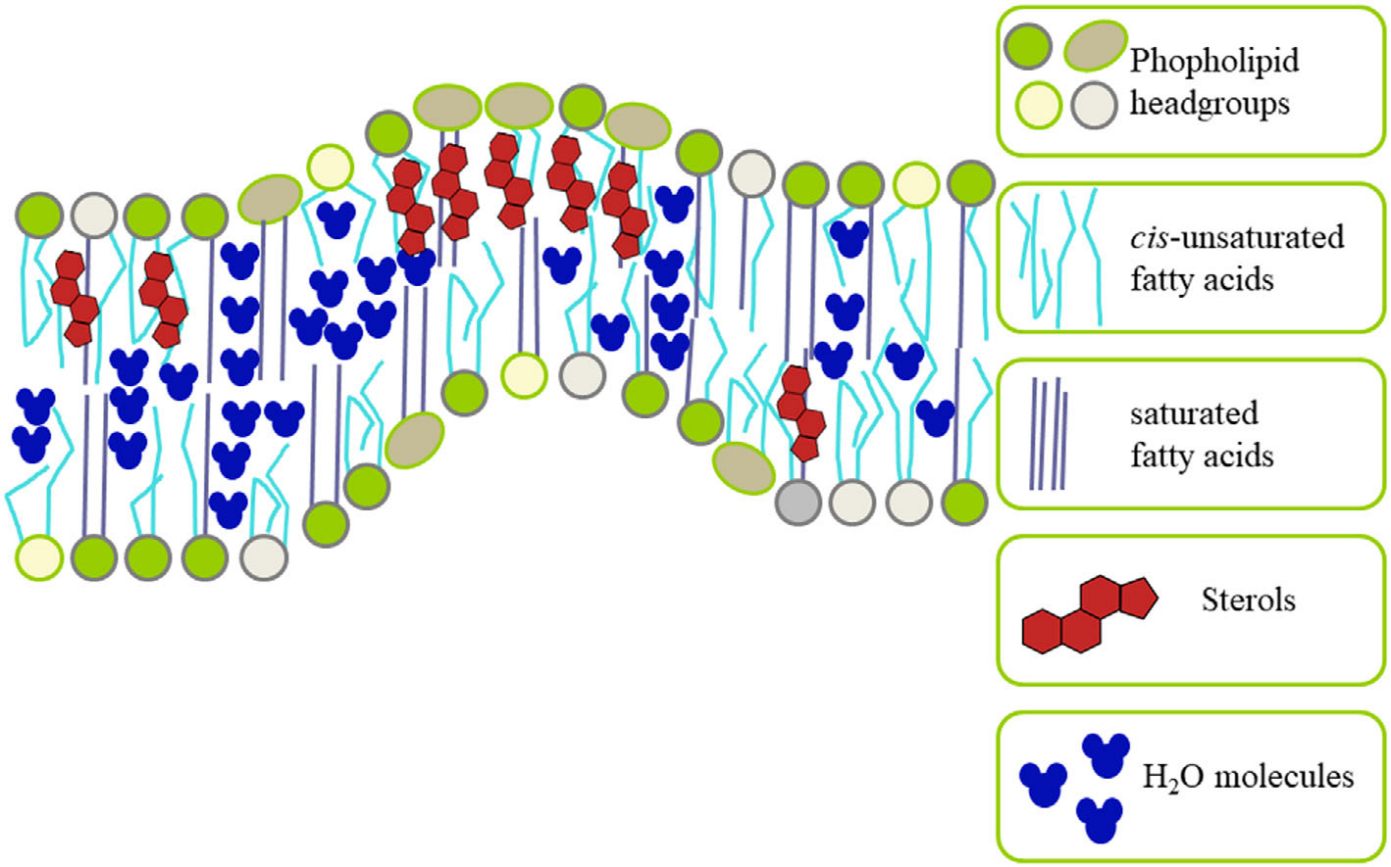
Sterols influence membrane fluidity of duckweeds and other plants. Phosphoglyerolipids, the main components of bio‐membranes, consists of a hydrophilic head group and hydrophobic fatty acids. Sterols are incorporated, with local peaks, and determine the specific properties of specific membrane areas. The water content within membrane leaflets is influenced by factors such as the quantity of membrane sterols and the characteristics (amount, chain length, and saturation) of fatty acids constituting phospholipids and sphingolipids. For instance, the presence of *cis*‐unsaturated fatty acids induces a change in the angle between adjacent carbons, creating space for water molecules. These water molecules enhances membrane fluidity. Likewise, uneven distribution of sterols in the leaflets can lead to asymmetries, promoting membrane curvature. These curves play a crucial role in molecular transport and endocytosis but can harm membrane integrity (Trautenberg *et al*. 2022).

Free phytosterols and their conjugates are produced by all known eukaryotic organisms, and probably even by the last common eukaryote ancestor (Brocks *et al*. 2023), for controlling membrane fluidity (Trautenberg *et al*. 2022; Fig. 4), while hopanoids are the respective molecules in bacteria (Nair & Kochupurackal 2023). The free hydroxyl group of free sterols (e.g., β‐sitosterol Fig. 3A) is important for the interaction of sterols with phospholipids and membrane proteins (Piironen *et al*. 2000). Some specific sterols are part of the control mechanism of membrane‐associated metabolism (Piironen *et al*. 2000). Sterols are not evenly distributed within cell membranes, leading to the formation of membrane curves (Fig. 4) which are necessary for transport and endocytosis but reduce cell membrane integrity (Trautenberg *et al*. 2022). That is why the biosynthesis of sterols is strictly controlled and regulated by a feedback regulation (Zhang, Li, *et al*. 2020; Zhang, Lin, *et al*. 2020). Phytosterol content and composition in the duckweed *Wolffia microscopica* is very similar to that in other monocot orders (Table 2). Unfortunately, available data are limited. For example, lipid extracts of *W. microscopica*, kernels of *S. cereale*, pulp of *Z. officinalis*, fibre of *A. officinalis*, oil of *C. nucifera* and yams of *Dioscorea alata* (Table 2) using different methods for sterol analysis. Therefore, a structured analysis of phytosterol content and composition in monocots is urgently needed to expand understanding of their physiological functions in crops necessary for the survival of billions of people.

**Table 2:** Phytosterol pattern in monocots as percentage of total sterol fraction.

sterol	*W. microscopica* lipid extracts (a)	*S. cereale* kernels (b)	*Z. officinale* (c)	*A. officinalis* fibre (d)	*C. nucifera* oil (e)	*D. alata* yam (f)
Campesterol	18%	20%	n.d.	≈29%	19%	13%
Stigmasterol	15%	5%	Identified	≈11%	10%	22%
Sitosterol	53%	59%	Identified	>50%	46%	65%
Sitostanol	n.d.	8%	n.d.	n.d.	n.d.	n.d.
Δ^5^‐Avenasterol	11%	n.d.	n.d.	n.d.	16%	n.d.
Δ^5,24(25)^‐Stigmastadienol	1%	n.d.	n.d.	n.d.	n.d.	n.d.
Cycloartenol	2%	n.d.	n.d.	n.d.	7%	n.d.
Total	500 mg kg^−1^	970–760 mg kg^−1^	n.d.	600–1000 mg kg−1	640–540 mg kg−1	190 mg kg−1

(a) Appenroth *et al*. (2017); (b) Zangenberg *et al*. (2004); (c) Marrelli *et al*. (2015); (d) Fuentes‐Alventosa *et al*. (2009); (e) Cui *et al*. (2023); (f) Lu *et al*. (2017)

**Fig. 5:**
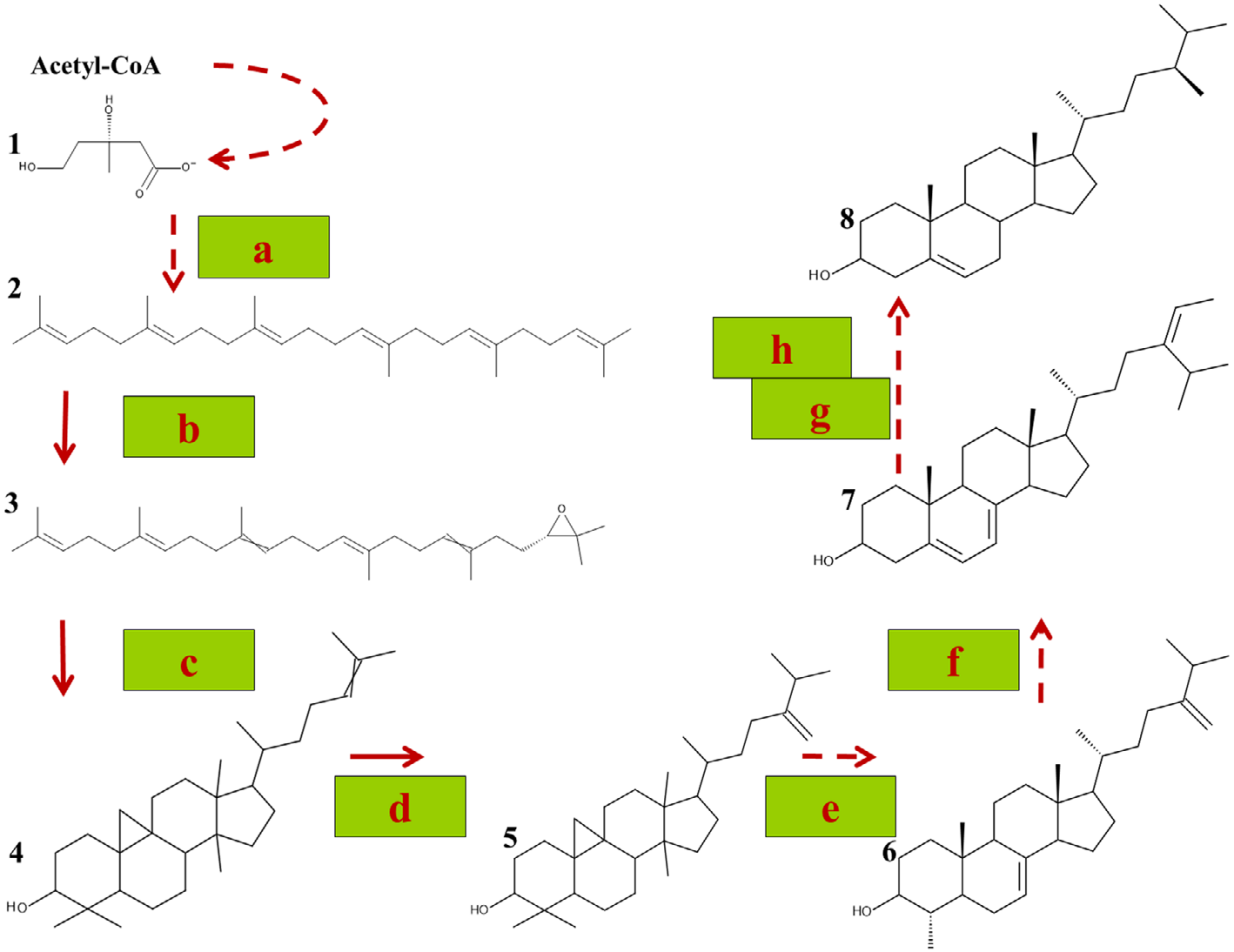
Phytosterol biosynthesis. Synthesis of phytosterols from Acetyl‐CoA follows the mevalonate pathway. R‐mevalonate (1) is converted by a multistep pathway into squalene (2). The last step (the conversion of two molecules of farnesyl diphosphate into squalene) is catalysed by squalene synthase (a). A squalene epoxidase (b) converts squalene (2) into 2,3‐oxidosqualene (3) which is converted by a cycloartenol synthase (c) into cycloartenol (4), the first structure with sterane skeleton. A sterol methyltransferase (d) produces 24‐methylene cycloartenol (5) before a multistep reaction (including a sterol 8,7‐isomerase; e) for formation of the sterane backbone in 24‐methylene lophenol (6) completes. A C4 demethylase and a Δ7‐sterol‐C5 desaturase (f) lead to the formation of 5‐dethydroavenasterol (7), before a Δ7‐sterol‐C5 reductase (g) and a C24 reductase (h) for formation of campesterol (8) complete. The enzymes a–h can be found in the transcriptome of *S. polyrhiza* (Supporting Information S2).

Sterols are produced from Acetyl‐CoA by the mevalonate pathway (Fig. 5; Goswami *et al*. 2023). Most enzymes involved were characterized in *A. thaliana* (Goswami *et al*. 2023). Homologues of these enzymes can also be found in the transcriptome of *S. polyrhiza* (e.g., using Phytozome 13). From the protein sequences of the monocot species *A. americanus*, *A. officinalis*, *D. alata* and *S. polyrhiza*, the enzymes in Fig. 5 were identified on Phytozome 13 (Goodstein *et al*. 2012) and can be found in Supporting Information S2. These sequence data were produced by the US Department of Energy Joint Genome Institute. We added protein sequences from *S. cereale* (https://plants.ensembl.org/Secale_cereale/Tools/Blast?db=core; Harrison *et al*. 2024), *C. nucifera* and *Z. officinale* (https://www.ncbi.nlm.nih.gov/). As the sterol content and profile are similar across several monocot species, it is not surprising that these enzymes show high identity between these monocot species (Supporting Information S2).

Phytosterol homeostasis must be strictly regulated to avoid damage to cell membranes. Hence, the phytosterol surplus forms sterol conjugates, for example, steryl esters, steryl glycosides, and acyl steryl glycosides (reviewed in: Ferrer *et al*. 2017; Zhang, Li, *et al*. 2020; Zhang, Lin, *et al*. 2020; Rogowska & Szakiel 2020). Steryl esters (SE) are produced by sterol acyl transferases (Ferrer *et al*. 2017). In *A. thaliana* two sterol acyl transferases (phospholipid:sterol acyltransferase and acyl CoA:sterol acyltransferase; Banas *et al*. 2005; Chen *et al*. 2007) were described, but the phospholipid:sterol acyltransferase is of higher importance for formation of SEs. SEs hardly interact with the cell membrane and have an important role in cell membrane sterol homeostasis. They can be considered as a sterol storage that can be activated during growth and development (Schaller 2004). SEs accumulate in cytosolic lipid droplets and are also a transport form of sterols. High levels of SEs can be found in the phloem sap, with cholesterol ester being the most abundant phytosterol (Behmer *et al*. 2013). During senescence this transportable form is also used for recovery of sterols (Bouvier‐Navé *et al*. 2010).

Glycosylated sterols (steryl glycosides, SGs; acylated steryl glycosides, ASGs) are at much lower concentrations than free sterols and SEs, except in *Solanum* (Solanaceae; Nyström *et al*. 2012). Sterol glycosyltransferases transfer a monosaccharide to the hydroxyl group at C3 of the sterol backbone, and build a β‐glycosidic bond (Chaturvedi *et al*. 2011). The most abundant sugar in glycosylated sterols is glucose, but galactose, mannose, and xylose have also been found, also sugar chains of up to four monosaccharides (Ferrer *et al*. 2017). In *A. thaliana* there are two sterol glycosyltransferases (UGT80A2 and UGT80B1; DeBolt *et al*. 2009). Analysis of knock‐out lines indicate participation of these enzymes in seed formation, and seedling root development (DeBolt *et al*. 2009). Steryl glycoside acyltransferase activity is detected in protein extracts from many plant organs and tissues, but no gene has yet been found to encode a steryl glycoside acyltransferase in any organism (Ferrer *et al*. 2017). Interestingly, we only detected a homologue to UGT80A2 in *S. polyrhiza* but no homologue to UGT80B1 (Supporting Information S2). Known enzymes with high identities for the production of sterol conjugates have been found in the monocot species *Acorus americanus*, *A. officinalis*, *C. nucifera*, *D. alata*, *S. cereale*, *S. polyrhiza* and *Z. officinale* (Supporting Information S2).

Both free and conjugated sterols impact plant responses to stress (Ferrer *et al*. 2017; Rogowska & Szakiel 2020). UV‐B results in increased levels of stigmasterol and decreased levels of campesterol, crinosterol, and cholesterol in the leaves of *Withania somnifera*, while SEs accumulated in roots of these plants (Takshak & Agrawal 2015). In *Vitis vinfera* changes in sterol profiles are more obvious at low UV levels (Gil *et al*. 2012). Duckweed species form a single layer on unshaded freshwater surfaces with high light intensities, including UV. We consider that this makes duckweeds a great model for analysis of the effects of UV radiation on sterol formation. Enhanced sterol content has been observed in plants exposed to cold stress (Rogowska & Szakiel 2020), including *A. sativa* (Takahashi *et al*. 2016), *S. cereale* (Takahashi *et al*. 2016), and *T. aestivum* (Valitova *et al*. 2016). Some of the duckweed species like *Lemna minor* can thrive in the harsh cold season of the temperate climate and could be used as monocot models for understanding the role of plant sterols in cold stress response.

#### Phytosterols and their role in human nutrition

Phytosterols have anti‐diabetic (Babu & Jayaraman 2020), anti‐cancer, anti‐inflammatory, and anti‐infective properties (Bakrim *et al*. 2022). Unsaturated phytosterols also act as antioxidants (Bakrim *et al*. 2022). However, the role of phytosterols in human nutrition as cholesterol‐lowering metabolites is debated. Half of cholesterol is synthesized in the liver, but all human cells can produce cholesterol (Luo *et al*. 2020). Cholesterol in human cell membranes interacts with membrane lipids and regulates rigidity, fluidity, and permeability of the bilayer. In addition, cholesterol can bind transmembrane proteins, helping to maintain or alter their conformation. Cholesterol also interacts with sterol transport proteins that facilitate cholesterol trafficking and regulate its subcellular distribution (Luo *et al*. 2020). It is also a sterol precursor for all steroids in the human body (Miller & Auchus 2011). While nearly every human cell can synthesize cholesterol, most cells are unable to degrade it, except for hepatocytes, adrenal cells, and gonad cells. Cholesterol homeostasis must be tightly regulated (Luo *et al*. 2020) as high values are an increasing problem of dietary choices. Phytosterols in human cells/serum are completely attributed to human consumption (Scholz *et al*. 2022), with daily uptake ranging from 150 to 450 mg (de Vries *et al*. 1997; Ostlund 2002; MacKay & Jones 2011). Therefore, in a normal diet, cholesterol and phytosterols levels are similar (Scholz *et al*. 2022), and phytosterols can reduce cholesterol levels (Pollak 1953) through several mechanisms:

1. Phytosterols inhibit uptake of cholesterol in the small intestine (Ferguson *et al*. 2016).
2. Phytosterols replace cholesterols in mixed micelles (Plat *et al*. 2019).
3. Δ^22^‐phytosterols inhibit endogenous cholesterol biosynthesis by competitive inhibition of the Δ^24^‐reductase (Fernández *et al*. 2002).

However, phytosterol levels in human serum are strictly controlled and around 200 times lower than that of cholesterol (Chan *et al*. 2006). Individuals with enhanced sitosterol serum levels (e.g., caused by SNPs in transport proteins, ATP‐binding cassette sub‐family G member 5 or ATP‐binding cassette sub‐family G member 8) have enhanced risk of coronary artery disease (Scholz *et al*. 2022).

### Duckweed species as a tool in phytosterol biotechnology

Healthy nutrition provides sufficient micronutrients and bioactive plant metabolites, like phytosterols. Development of crops with enhanced micronutrient levels is a goal of plant science. In contrast to many model organisms used in biotechnology (e.g., *Escherichia coli* or *Saccharomyces cervisiae*) Lemnaceae produce phytosterols endogenously. Moreover, these fast‐growing organisms are used in food in several Asian countries and as novel food in Europe (EFSA 2021, 2022; EU Regulation 2025). One strategy to enhance nutritional values by genetic engineering introduced a sterol 22‐desaturase from *Solanum lycopersicum* into *Lemna minor* to enhance the content of stigmasterol (Yang *et al*. 2018). Unfortunately, little is not known about the total sterol content in transgenic *L. minor* lines.

Below are some strategies that can be tested in Lemnaceae species to enhance phytosterol content:

- Overexpression of essential enzymes for phytosterol production, such as the cycloartenol synthase (Supporting Information S2): Gas‐Pascual *et al*. (2014) showed that reduced expression of CAS1 in tobacco leaves reduced phytosterol levels. This indicates that overexpression of this locus might enhance phytosterol levels. In transgenic ginseng, overexpression of squalene synthase (Lee *et al*. 2004) and squalene epoxidase (Han *et al*. 2020) had enhanced phytosterol levels.
- Inducible promoters in plant cells might overcome current limitations. Cultivation of transgenic duckweed and expression of a transgene enzyme could ensure production of significantly enhanced amounts of phytosterols. Use of salicylic acid‐responsive promoters (e.g., Cestrum yellow leaf curl virus (CmYLCV)) promoter; Sarkar *et al*. 2018) or endogenous plant promoters of *pathogenesis*‐*related protein 1C*‐*like* (*PR1*) and *glucose endo*‐*1*,*3*‐*beta*‐*glucosidase* (*BGL*) (Wu *et al*. 2024). Salicylic acid is an antithrombotic, reduces fever, inflammations and pain (Fiala & Pasic 2020) and is a promising and safe metabolite for transgene expression in duckweeds to be used for human consumption.
- Inhibiting conversion of phytosterols into their derivatives using gene knock‐down or knock‐out. Monocots convert phytosterols into a range of derivatives such as brassinosteroids. A simultaneous knock‐down of three enzymes needed for the conversion of phytosterols into brassinosteroids four‐fold enhanced campesterol in *A. thaliana* (Chung *et al*. 2010). Knock‐down plants show a typical dwarf‐mutism, but inducible inhibition of this pathway could enhance phytosterol levels in duckweeds.

### Phytosterol derivatives

Apart from brassinosteroids (see above), monocots can convert phytosterols into pharmaceutically important metabolites like progestogens, androgens, corticosteroids, oestrogens (Shiko *et al*. 2023), or steroid saponins (e.g., avencoside A from *Avena*), and 5β‐cardenolides (e.g., convallatoxin from *Convallaria majalis*). These metabolites are not produced by wild‐type duckweeds but could be genetically engineered to produce pharmaceutically relevant substances at industrial scale.

In the following, the typical derivatives of phytosterols (brassinosteroids, steroids, and specialized metabolites) are described.

#### Brassinosteroids

**Fig. 6:**
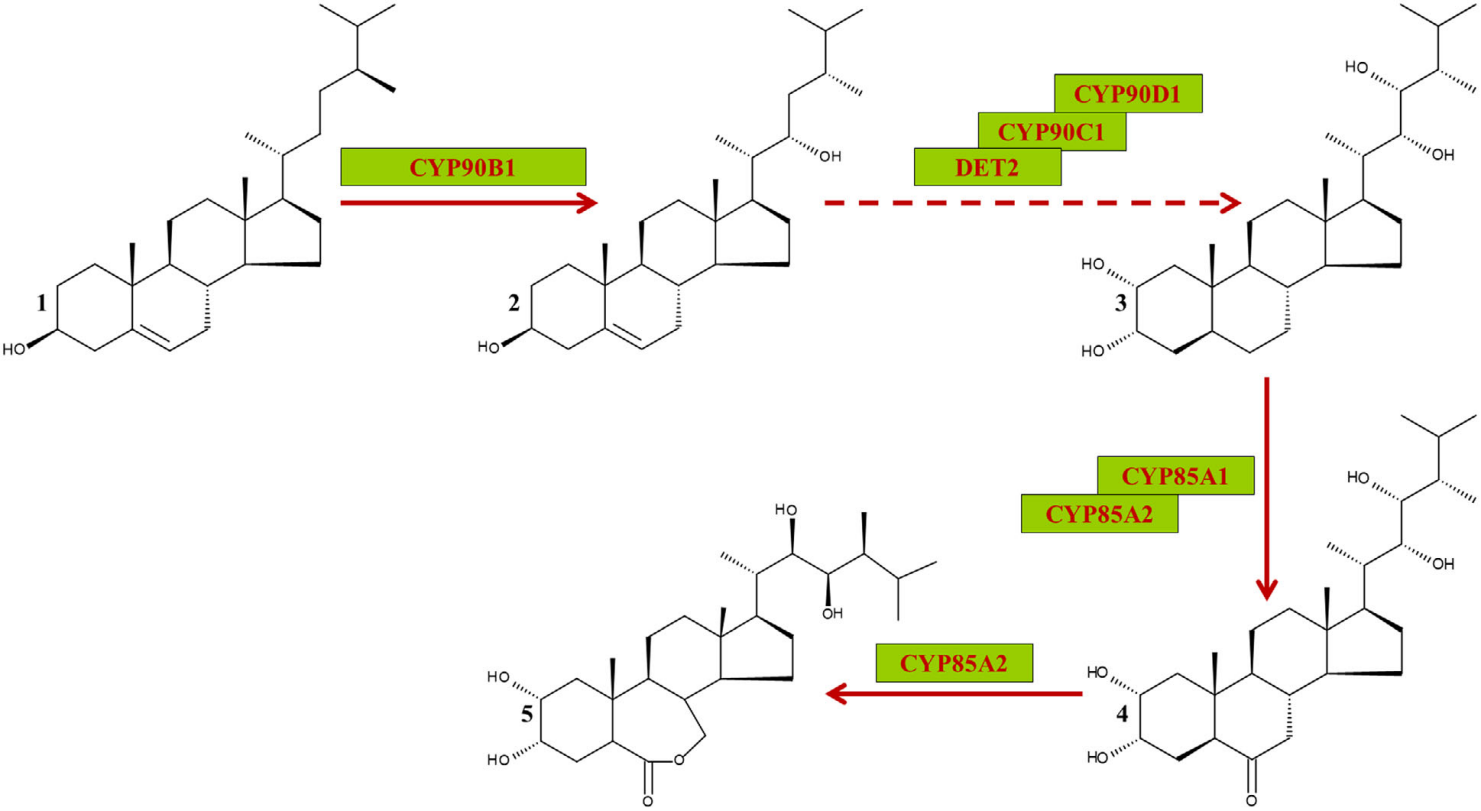
Brassinolide biosynthesis. The pivotal and rate‐limiting step of brassinosteroid (BR) biosynthesis is catalysed by CYP90B1. This catalyses the conversion of campesterol (1) into (22S)‐22‐hydroxycampesterol (2). In the following, DET2 reduces the double bond, while CYP90C1 and CYP90D1 insert hydroxyl groups at C2 and C23. This results in the formation of 6‐deoxocastasterone (3). 6‐deoxocastasterone is carbonylated at C6 by CYP85A resulting in castasterone (4), the final BR form in monocots, while castasterone is lactonized in dicots to the final BR brassinolide (5). Interestingly, CYP90C1 homologues have not been identified in monocots except *Acorus americanus*, raising the question of which enzyme catalyses this reaction in other monocot species (see Fujiyama *et al*. 2022).

Phytosterols can be converted into brassinosteroids (BRs). The biosynthesis of BRs in plants is well‐characterized (Poppenberger *et al*. 2024). BRs are important growth‐regulating phytohormones (Peres *et al*. 2019). Consequently, a disturbance in BR biosynthesis or signalling leads to strong dwarf phenotypes. An overview of BR biosynthesis is provided in Fig. 6.

Enzymes of BR biosynthesis are conserved within plants (Fujiyama *et al*. 2022). The respective genes in *A. thaliana* could also be identified in *S. polyrhiza* (e.g., using Phytozome 13) with exception of CYP90C1. The protein sequences of *A. americanus*, *A. officinalis*, *D. alata* and *S. polyrhiza* have been identified (Supporting Information S3) by the US Department of Energy Joint Genome Institute. Protein sequences from *Secale cereale* (https://plants.ensembl.org/Secale_cereale/Info/Index; Harrison *et al*. 2024), *C. nucifera* and *Z. officinale* (https://www.ncbi.nlm.nih.gov/) have been added.

**Fig. 7:**
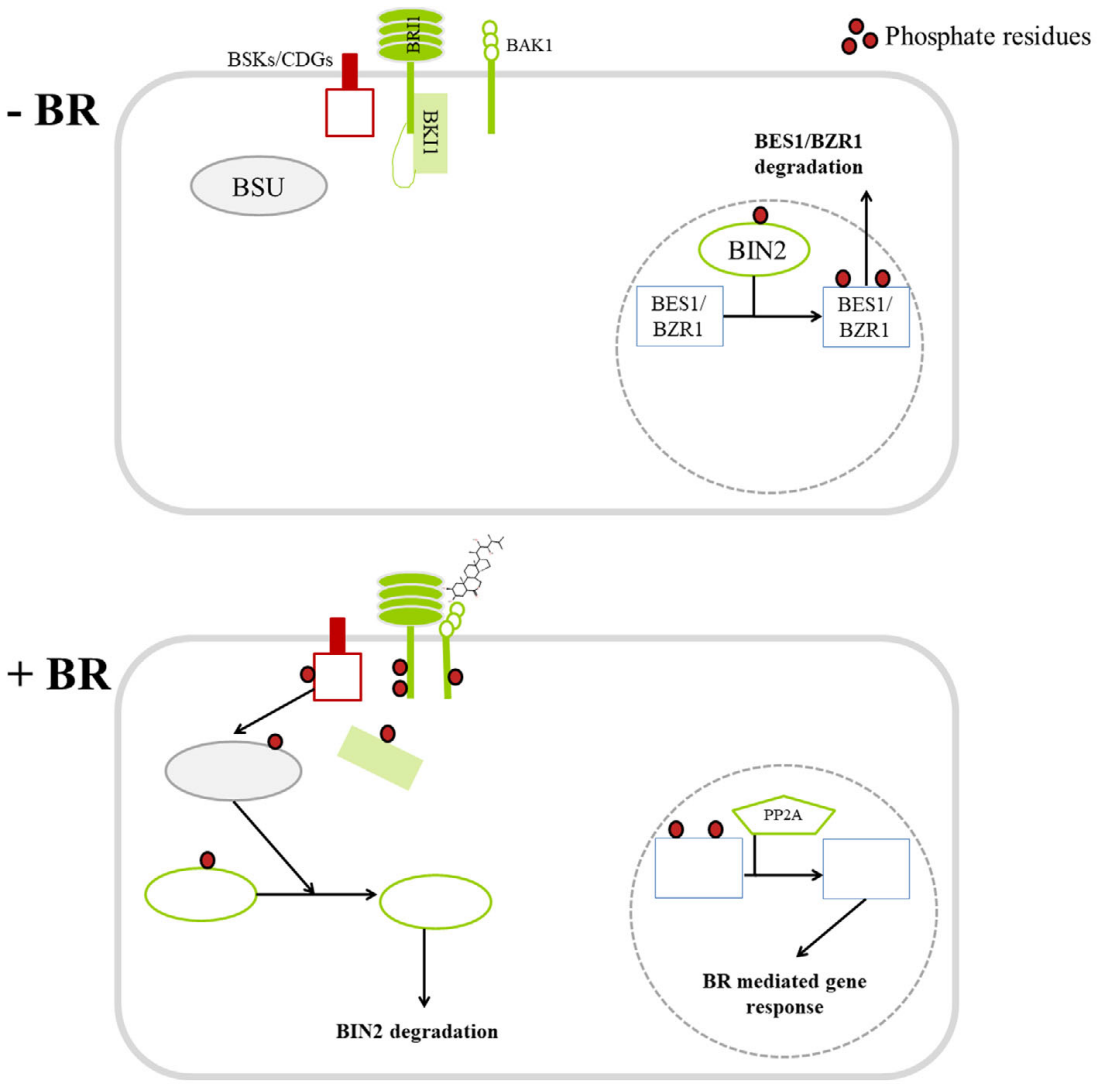
Simplified model of brassinosteroid signalling according to Kim & Russinova (2020). In the absence of brassinosteroids (−BR), the brassinosteroid receptor Brassinosteroid‐Insensitive1 (BRI1) remains inactive due to the autoinhibitory C‐terminus and its association with BRI1 Kinase Inhibitor1 (BKI1). Brassinosteroid‐Insensitive2 (BIN2) is constitutively active and phosphorylates the transcription factors BRI1‐EMS Suppressor1 (BES1)/Brassinazole‐Resistant1 (BZR1). This phosphorylation promotes their 14‐3‐3‐mediated degradation within the cytosol and thereby inhibits their DNA‐binding activity. On the other hand, when BRs are present (+BR) and bind to the extracellular domains of BRI1 and its co‐receptor BRI1 Associated Receptor Kinase1 (BAK1), the two receptor kinases are activated. This activation leads to the dissociation of BKI1 from BRI1, as well as the phosphorylation and activation of BR‐Signalling Kinases (BSKs)/Constitutive Differential Growth (CDGs) and BRI1 Suppressor1 (BSU1). Activated BSU1 dephosphorylates and inactivates BIN2, which, in consequence, will be degraded. This cascade of events results in the nuclear accumulation of Protein Phosphatase 2A (PP2A). This phosphatase dephosphorylates BES1/BZR1, which then binds to BR Response Element (BRRE)/E‐box‐containing promoters. This binding regulates the expression of numerous BR‐responsive genes crucial for plant growth and development.

Brassinosteroid signalling components have been identified, but mainly on the model plant *A. thaliana* (cf. Kim & Russinova 2020; Mao & Li 2020; Fig. 7). The BR signalling pathway is well‐characterized (Poppenberger *et al*. 2024). Most elements in BR signalling in *A. thaliana* can be found in the transcriptome of *S. polyrhiza* (e.g., using Phytozome 13). The protein sequences of the monocots *A. americanus*, *A. officinalis*, *D. alata* and *S. polyrhiza* in Fig. 7 were identified on Phytozome 13 and can be found in Supporting Information S4. We added protein sequences from *S. cereale* (Harrison *et al*. 2024), *C. nucifera*, and *Z. officinale*. Interestingly, no BKI1 homologue could be found in *S. polyrhiza*. Consequences of this for BR signalling in duckweed species needs to be determined.

#### Brassinosteroids as target of crop breeding

Reduced plant growth (using semi‐dwarf varieties) was used enhance crop yields in the 1960s (Zeigler & Mohanty 2010), and growth regulators are still an important topic in crop biology. Unfortunately, this green revolution was based on increased chemical and water input. Some chemicals were plant growth regulators (PGRs) that inhibit gibberellic acid synthesis or signalling like chlormequat (Rademacher 2000). Now PGRs are known to have serious disadvantages:

1. PGRs impact the environment by affecting non‐target organisms and accumulating in soil and water resources (https://lfu.brandenburg.de/cms/media.php/lbm1.a.3310.de/lfu_fb_151.pdf).
2. PGRs are work‐intensive and enhance fuel consumption, thereby increasing the carbon footprint of plant products.
3. PGR use must be adapted to regional situations and local weather and, therefore, optimal PGR use can change every year.

An alternative strategy to ensure yield stability could be crop plants with reduced growth by targeting BR biosynthesis and signalling. However, BR biology is a complex. Several strategies need to be examined to find ways to adapt BR biology of crop plants to the needs of agriculture in a changing environment. In this context, Lemnaceae can be suitable model organisms, with transformation protocols in place and high biomass production capacity.

#### Progestogens, androgens, corticosteroids, and oestrogens

Plants can synthesize progestogens (C_21_‐steroids; Simons & Grinwich 1989; Shiko *et al*. 2023), androgens (C_19_‐steroids; Simons & Grinwich 1989; Shiko *et al*. 2023), corticosteroids (Caspi *et al*. 1968), and oestrogens (Geuns 1978) from sterol or steroid precursors (Klein 2024). The first plant metabolite with a steroid structure was an oestrogen (Skarzynski 1933). The metabolism of progestogens and androgens represent catabolism and anabolism of progesterone (Klein 2024).

Interestingly, the main steps from progesterone to the androgen androstenedione (17α‐hydroxylation and 17,20‐lyase reaction) are most efficient in *Hordeum vulgare* and *S. polyrhiza*. The ability of duckweed species like *S. polyrhiza* to take up and convert progestogens and androgens offers several applications:

1. Duckweeds as filter systems to remove hormone contamination from wastewater: duckweeds grow rapidly (Sree *et al*. 2015; Ziegler *et al*. 2015) and produce a huge amount of biomass in a short time. Moreover, duckweed biomass can easily be separated from the surrounding water compared to microorganisms and algae (which often must be removed by membrane filters or by centrifugation), making duckweeds ideal organisms for wastewater remediation (Ziegler *et al*. 2017).
2. Wastewater is often contaminated with diverse man‐made chemicals, including artificial steroid compounds used as medicinal products. Progestogens, androgens, and oestrogens can be found in wastewater throughout the world (Chang *et al*. 2011; Gimiliani *et al*. 2016; Johnson & Chen 2017; Budeli *et al*. 2021; Yu *et al*. 2022). These compounds are harmful even at picomolar concentrations (Burkhardt‐Holm 2010). In mammals, they act as endocrine disruptors (Burkhardt‐Holm 2010; Gimiliani *et al*. 2016) and adversely affect human and animal health and reproduction (Burkhardt‐Holm 2010). Unfortunately, removal of trace biologically active steroids is complicated and cost intensive. As duckweeds can take up and convert progestogens and androgens (Shi *et al*. 2010; Shiko *et al*. 2023), and the synthetic oestrogen quinestrol (Geng *et al*. 2018), they could be used as green steroid filters.
3. Duckweeds as model organisms for engineering plants with enhanced stress resistance. In contrast to brassinosteroids, progesterone can activate the ROS detoxification machinery in plants (Klein 2024), and perhaps change membrane fluidity (Oktay *et al*. 2025). Exogenous treatment or enhanced production of progestogens (e.g., in *Musa nana*) enhances plant resilience to stress (Klein 2024).

#### Metabolites of specialized monocot metabolism

Monocot species produce a broad range of specialized metabolites based on sterols or steroids, e.g., the steroid saponin avenacosides (e.g., avencoside A) from *Avena* and 5β‐cardenolides (e.g., convallatoxin) from *C. majalis*. Avenacosides are steroidal saponins with more than one sugar moiety in *A. sativa* (Tschesche *et al*. 1969). They have several positive effects on human health (e.g., inhibit survival of pancreatic cancer cells; Kim *et al*. 2019). However, their concentrations in *Avena* are too low for industrial scale medicinal use.

The 5β‐cardenolides, like convallatoxin, have been used for treatment of cardiac insufficiency and supraventricular tachycardia since identification of the active principle in *Digitalis* species in 1844 and 1845. Today use of cardenolides as therapeutic agent for heart insufficiency is outdated, but they might prove a therapeutic agent for malignant tumours (Anderson & Barton 2017; Zhang, Li, *et al*. 2020; Zhang, Lin, *et al*.^2020^; Hafner *et al*. 2021). While there has been progress in analysis of cardenolides from *Digitalis* (Klein *et al*. 2021) and *Erysimum* (Munkert *et al*. 2011, 2014; Horn *et al*. 2021), their biosynthesis in monocots is still poorly understood.

For the medicinal use of avenacosides and cardenolides, biotechnological production would be of great benefit. Based on the advantages of duckweeds (fast growth, small genome, vegetative propagation, ease of cultivation, established transformation), we are convinced the use of duckweed for production of sterol‐ or steroid‐based specialized metabolites for pharmaceutical issues is a promising strategy.

## CONCLUSIONS

Duckweeds represent a compelling model system with considerable potential for advancing plant science. We propose their strategic utilization to drive significant breakthroughs in the study of sterol and steroid metabolism in monocotyledon species, with findings that might translate to crop plants. Such advances are critical for addressing pressing global challenges, including, but not limited to, enhancing the nutritional value and stress tolerance of crops, elucidating the physiological roles of progestogens and androgens in plants, and enabling the sustainable, low‐carbon footprint production of pharmaceutically relevant sterol derivatives, such as cardenolides.

## AUTHOR CONTRIBUTIONS

All authors contributed to the study. **JK** designed the study and wrote the first draft of the manuscript with **KSS. KJA** commented on all versions of the manuscript. All authors read and approved the final manuscript.

## CONFLICT OF INTEREST STATEMENT

The authors have no conflict of interest.

## Supporting information


**Data S1.** DET2 sequences.

**Data S2.** Sterol biosynthesis.

**Data S3.** Brassinosteroid biosynthesis.

**Data S4.** Brassinosteroid signaling.
