# Supplementary material for: Duckweeds: Model organisms for research on plant sterols and steroids
Source: Plant Biol (Stuttg). 2025 Aug 25;28(1):18–30. doi: 10.1111/plb.70095 (PMC12710849; doi:10.1111/plb.70095)
Supplement: Supplementary file 1 — Data S1. DET2 sequences. [file PLB-28-18-s002.pdf]

>*Secale_cereale*_DET2_CDS
ATGTCCGACGGAGGCGGCGGAGCCGCGGGCGGCGACGCGCTGTTCTCGCGGTGCCTGCTCG
CGCTCTACGTCATCAGCCCGGTCACCGTCTTCCTGCTCCGCTACGTCTCCGCGCCCTACGGCA
AGCTCTCGCGCCCGGGGTGGGGCCCGGCCATCCCGGCCGCGCTGGCCTGGTGCGCCATGGA
GAGCCCCACCCTCTGGCTCCCGCTCCTCGTCTTCCCCCCGCCGCTGCTCGCCGCCGCGGCCGC
CTCCCCGCTCGCCGCGCTCCCGCCGGCGCTCTACGCGCTCCACTACGTCAACCGCACGGTCC
TCCACCCGCTGCGCATCTTCCGCCTGCGCCGCGCCGCGGCGCCCGTCCCCGTGCTCGTCGCC
GCCTGCGCCTTCGGATTCAACCTCCTCAACGCCTACGTCCAGGCCCGCTCCTGGGCGCTCCA
CGCCGCCCGCCCCGCATCCGCCTTCGCGCTCGCCCGCTCCCTCGTCGGGCTCGCCCTCTTCGC
GTGGGGGATGCGGGTCAACCTCGCGGCGGACAAGGAGCTCCTGCGGCTCAAGGAGGCCGGG
GGCGGCTACAAGATCCCGCGCGGCGGCTGGTTCGACGCGGTGACCTGCCCCAACTACTTCGG
CGAGATCGTGGAGTGGCTCGGCTACTGCTTGGTGGCCTGGTCGCCGGCGGCCTGGGCCTTCT
TCCTCTACACCTGCGCCAACCTCATGCCGAGGGCCAGGGACCACCGGCAGTGGTACCTCAGC
AAGTTCGGCGGCGAGTACCCGGCGTCGCGCAAGGCGGTCATCCCGTGCATCTACTAG

>*Spirodela_polyrhiza*_DET2_CDS
ATGGCGTCCGACGAGGCCCTGTTCTACGCAGCCGTCGGCATCCTCTACGCCATCTCCCCCCT
GACCTTCCTTCCCCTCCAATCCTTCACAGCCCCCTTCGGCCGCCATGCCCGCCCCGGCTGGGG
CCCTTTCCTCCCGCCGGCCGCCGCCTGGTTCCTCATGGAGAGCCCCACCATCTGGCTCACCCT
CCTCCTCCTCCCTCACGGCCGCCACCGCTCCCACCCGCTCTCTCTCGCCATCATCTCCCTCTA
CCTCCTACACTACCTCAATCGCACCGTCGTCTACCCCCTCCGCCTCCTACGCAGCGGCACCAA
GACGGGCTTTCCCCTGTGCACCGCCGCCGCCGGCTTCTGCTTCAACCTCCTCAACGCCTACGT
CCAGACCCGGTCGGCCTCCCACTACGCCGACTACCCGCCGGCCGGAGACGTCGGCTGGTGG
GTGTGGGCGAGGGTGGCAGCGGGGCTGGCCGTGTTCTTCTGGGGCATGACCGTGAACGTCTC
ATCAGATCTGGCGTTGCTGAGGCTAAAGAAGACGGCGGGCGGCGGGTACAAGGTGCCGGAG
GGCGGATGGTTCGAGCTCGTGGCCTGCCCCAACTACATGGGGGAGGCGGCGGAGTGGCTGG
GCTGGGCTGTGGCGGCGTCCACGCCGGCGGCTCTCGGCTTCTTCCTCTACACCTGCGCCAAT
CTCGTACCCCGGGCCTCTTCTCACCGCCGCTGGTATCTGCAGAAGTTCGGCCCCCGCTACCCG
CCGTCCAGAAAGTGCATCGTACCTTTCGTCTTCTAA

>*Daucus_carota*_DET2_CDS
ATGGAGTTTTCCGATGAGTCTCTTTTCAACGCCTCTCTCCTCACTCTCTTCATCATGGGCCCA
CCCACTTTCATAGCCTGCCAATTCCTCACTGCCCCATATGGCAAACACCACCGTAAGGGCTG
GGGCCCCACCATCTCTCCACCGCTGGCCTGGTTCCTCATGGAAAGCCCAACTCTCTGGCTCA
CTCTCTTTATCTTCCCGTTTGGCAAAAATTACCATGACCCAAAAGCCCATATCCTCATTTCAC
CCTTTCTGTTTCACTATTTTCACCGCACTGTAATTTACCCTTTAAGACAATACTTGAAAATCC
GGCGCCGGAAAACCGCCAGTGGTTTTCCGGTGAGTGTGGCGGGGATGGCGTTTGGGTTTAAT
GTGTTGAATGCGTATTTGCAGGCGAGATGGGTTTCTCATTATGCTGAGTTTGAGGGGGATGA
GTGGTTTTGGTGGCGGTTTGCCGGCGGGTTGGTGGTTTTTGTGAGCGGTATGGCGGTGAATG
TACGGTCAGATATGGTTTTGTTGGGGTTGAAGAGTGAGGGGGGTGGGTATAAAATACCCAG
GGGTGGGTTGTTTGAGTGGGTCAGTTGTCCTAATTATTTTGGGGAGATTGTGGAATGGGCCG
GGTGGGCTATGATGAGCGGGTCTCGGGTCGGGTTCGGGTTTTTTGCTTATACTTGTGCTAATT
TAGTGCCAAGAGCAGGTGCTAATCATAGGTGGTATTTGGAGAAGTTCGGGGAGGATTATCC
GAAGAAGAGAAAAGCTGTGATTCCGTTTGTGTATTGA

>*Nymphaea_colorata*_DET2_CDS
ATGGCGTTGCTGCTAGAGTGGGGAGACGAGGCCTTCTACACGTATGCGCTGATCTCACTCTT
CGTCTTCTGTCCCCTCACCTTCCTCCCTCTCCTCGTCCTCCGCTTCCATGCACCCTACGGCAAG
CACACCCGCGGAGGATGGGGTCCCCTTCTTCCACCTTCCCTCGCTTGGTTCCTCATGGAGAGC
CCCACCGTCTGGCTCACCCTCCTCCTCCTTCCTTTCGGCCGCCATCGCTCCGATCCCGTCGCT
CTCGCTCTCCTCTCTTTCTACCTATTCCATTACATCCACCGCACCCTCATCTACCCGCTTGTCA
TCCGAAAAGCGCCTGCCTCCTCCTCCAGCCGCGGCGTGCCCTTCGCCGTCGCCTTCGCCGCCT
TCGTCTTCAACCTCCTTAATGCCTACCTCCAGAGCCGATGGATAACCCACTTCGGTAACTACA
CGCCAGGATGGCTACGGACGCCGAAGTTTGCGGCGGGGGCGGCGGTCTTTGCCATCGGCAT
GGCGACGAATGTGTGGGCAGATAGGAGGCTGGTGGCGCTGAAGAGGGAGGCGGGGGGAGG
GTACAAGGTTCCCAAAGGAGGACTCTTCGAGTTGGTCAGCTGCCCCAACTACACGGGGGAG
ATAATGGAGTGGCTGGGCTGGGCGATTATGACATGGTCGTTGGCGGGGCTGGCGTTCTTCCT
CTACACAGCGGCGAACCTGGTGCCTAGGGCGGCCGGCCACCACCAGTGGTACCTGCAGAAG
TTCCCCGAGGAGTATCCCAGGTCCAGGAAGAGGGTTATCCCCTTCATCTATTGA
