# Supplementary material for: Duckweeds: Model organisms for research on plant sterols and steroids
Source: Plant Biol (Stuttg). 2025 Aug 25;28(1):18–30. doi: 10.1111/plb.70095 (PMC12710849; doi:10.1111/plb.70095)
Supplement: Supplementary file 2 — Data S2. Sterol biosynthesis. [file PLB-28-18-s004.pdf]

Squalene synthase

***>S._polyrhiza*_SQS**
**MGLGSLWTIIDHPEDLMPLIRLKMASARIEKEIPAEPHWAFCYGMLTKVSRSFALVIQQLCTELRNAI
CIFYLVLRALDTVEDDTSVPSDIKVPILLSFHQHIYDKNWHFSCGTNNYKVLMDKFDLVSTAFLELDR
SYQEVIEDITRRMGAGMAKFICKEVETVEDYNEYCHYVAGLVGLGLSKLFHASGLEDLASDSLSNSM
GLFLQKTNIIRDYLEDINEIPKSRMFWPRQIWSKYANKLEDLKFEKNSINAVQCLNDMVTNALMHGE
DCLQYMSNLRNPAIFRFCAIPQIMAIGTLALCYNNIEVFRGVVKMRRGLTAQIIEHTSAMPDVYCAFF
EFASMLKDKVEENDPNAALALKYVETIQQACIKSGTLNKRRYYVGKSRRFLLVLITVLFALGSILFAR
FLKK***

*>S._cereale*_SQS
MGVLSRPEEVPALVRLKLAAGRIRRQIPPEAHWAFAYDMLQRVSRSFALVIQQLGPELRNAVCVFYLVLRA
LDTVEDDTAIPNEVKLPILRDFYRHIYNPDWLFSCGANDYRVLMDNFRQVSTAFLELGEGYQKAIEEITRR
MGAGMAKFICMEVETIDDYDEYCHYVAGLVGYGLSRLFHATGTEDLAPDHLSNSMGLFLQKTNIIRDYLE
DINEIPKCRMFWPREIWSKYVDKLEDLKYEENSEKAVQCLNDMVTNALIHALDCLQYMSALKDNSNFRFC
AIPQIMAIGTCAICYNNVKVFRGVVKMRRGLTARIIDETKSISDVYSAFYEFSSLLESKIDDNDPTAALTRMR
VGLIKETCKSSGLVKRRGYDLEKSKYRMPMLVMIMLLLVAIILGVLYGK*

*>A._americanus*_SQS
MAMMLRHPDEVLPLMKMKVASAMLKKQIPPEEHWSFCYTMLQRVSRSFALVIQQLGVELRNAVCIFYLV
LRALDTVEDDTSIPSDVKVPILESFYRHIYDRDWHFSCGTKDYKVLMDEFHHVSTAFLELGKSYQEAIEDIT
KKMGSGMAKYICKEVETVDDYDEYCHYVAGLVGLGLSKLFYASGKEELAPEFLSNSMGLFLQKTNIIRDY
LEDINEIPKSRMFWPHDIWSKYADKLEDLKYEENSENAVRCLNEMVTDALRHVDDSLKYMSSLRDLAIFRF
CAIPQIMAIGTLALCYNNLQVFRGVVKMRRGLVAKISDQTKTMSDVYSDFFEFSCLLKSKINEKDPNATLT
QKRIENIQNSCIASGFLKKRSYAGDGNSKYSSTLIILVFFLLSILVAYLTSK*

*>D._alata*_SQS
MGMMGALVENPGEVVALVKLKAAAAKIARQIPPEEHWAFAYTMLQKVSRSFSLVIQQLGPELRNAVCIFY
LVLRALDTVEDDTSIPADVKVPILQEFHRHIYDRDWHFSCGEKDYKVLMDKFHYVSTAFLELGKGYQEAIE
DITRRMGAGMAKFICKEVETVDDYDEYCHYVAGLVGLGLSKLFYAAGLEALASDSLSNSMGLFLQKTNII
RDYLEDINEIPKSRMFWPRQIWSKYANKLEDLKYEENSTKAVECLNDMVTNALMHAEDCLTYMAALKDI
AIFRFCAIPQVMAIGTLALCYDNLEVFRGVVKMRRGLTARVIDQTKTMADVYGAFYDFSSMLKAKVNDK
DPNAVLARQRVEAIQKICVSSGLLNKRGLQVCNTQPTISPMLIIVAVVLLAILLATLSK*

*>A._officinalis*_SQS
MGLIGTMLEHPEEAYALMKLKIAASKISKQIPPEPHWGFSYTMLQKVSRSFALVIQQLETELRNAVCIFYLV
LRALDTVEDDTSIPAEVKVPILQSFHRHIYDRDWHFSCGTKEYKVLMDKFHHVSTAFLELGKSYQEAIEDIT
KRMGAGMAKFICKEVETVDDYDEYCHYVAGLVGLGLSKLFHASGKEDLASDSLSNSMGLFLQKTNIIRDY
LEDINEIPKSRMFWPREIWSKYANKLEDFKYEENSXXXXITNALLHGEDCLQYMAGLKDTAIFRFCAIPQIM
AIGTLALCYNNLEVFRGVVKMRRGLTAKVIGQTRMMSDVYGAFFEFSALLKSKVDESDPNAALTHKRIEAI
QKACISSGKLDKKGYGYFNKQQRVNPVMVMMLVVLLAIIFGILSKK*

*>C._nucifera*_SQS
MGLLGAIVEHPEDLYALVKLKMAADRIKKQIPAEPHWVFSYTMLQKVSRSFALVIQQLGPELRNAVTSSPH
LPFPRYFLGFLVGFRVYVSYNVGLPFKRFDFLYFGDLFFLLGVNLVFGNDLRGRKDCKVLMDKFHSVSTAF
SELGASYQEAIEDITKRMGAGMAKFICKEVETINDYDEYCHYVAGLIGLGLSKLFHVSGLEELAPDSLSNSM
GLFLQKTNIIRDYLEDINEIPKSRMFWPQEIWSKYASKLEDLKYEENSTKAVQCLNEMVTNALMHAEDCLQ
YMSALKDLAIFRFCAIPQAWNSEITVIHISNPNFAVVIIANEIMAIGTLALCYNNVEVFRGVVKMRRGCSNRR
YEIMDHINCRDDFLPPFPHDSFKWDLPSHFTMYSHQGLTARVIEQTRSMSDVYGAFFEFSSLLKSKIDDNDP
NASLAHHHVEAIQKACISSGLLDKRGCHMYESRLLYNSALVNSCARYWSDVPYRCCF*

>*Z._officinalis*_SQS
MGLLGAIVAHPEELYPMVKLKMSMDRVRRQIPAEPHWHFSYSMLQKVSRSFALVIQQLRPQLRNAVCIFY
LVLRALDTVEDDTSIPSDIKVPILQSFYCHIYDSQWHFSCGTKEYKVLMDKFHLVSTAFLELDRSYQEAIEDI
TKRMGAGMAKFICKEVETVDDYDEYCHYVAGLVGLGLSKLFHASGSEDLASDNLSNSMGLFLQKTNIIRD
YLEDINEIPKSRMFWPREIWSKYADKLEDFKYAENSTKAVQCLNDLVTNALMHIEDCLHYMSSLKDLSIFQ
FCAIPQIMAIGTLALCYNNMQVFRGVVKMRRGLTARVIAQTETMADVYGVFFDFSSLLESKINDDDPNASL
TRKRVQAVKDRCISSGLLNKRKFHVHNRKSYTSLLIMVIVLVVAMLFSLPRRKVLVD*

>*P._polyphylla*_SQS
MGMMAAVLDHPGDLYVLVKLKMAASRIERQIPAEPHWAFAYTMLQKVSRSFAIVIQQLGTELRNAVCIFY
LVLRALDTVEDDTSIPSDIKVPILESFHRHIYDRDWHFSCGTKDYKVLMDKFHYVSTAFLELDKGYQEAIEE
ITKRMGAGMAKFICKEVETVDDYDEYCHYVAGLVGLGLSKLFHASGKEELPPDSLSNSMGLFLQKTNIIRD
YLEDINEIPKSRMFWPREIWSKYANKLEDFKYEENSTNALHCLNDMVTNALMHAEDCLQYMSALKNLSIF
RFCAIPQIMAIGTLALCYNNQELFRGVVKMRRGLVARVIDQTKTMSDTYGAFYEFSSLLKSKIDKKDPNAT
LTWERIEAIQKSCISSGLLNKRRCHMYESKPAYNSTMIMMAFLVISILFAILAIK*

C. nucifera SQS	100.00	65.16	66.00	64.25	69.63	68.98	72.03	70.10
S. cereale SQS	65.16	100.00	65.51	67.25	68.98	71.71	71.04	69.85
***S. polyrhiza* SQS**	**66.00**	**65.51**	**100.00**	**69.80**	**71.81**	**71.74**	**73.77**	**73.13**
A. americanus SQS	64.25	67.25	69.80	100.00	70.79	73.51	76.05	74.44
Z. officinalis SQS	69.63	68.98	71.81	70.79	100.00	74.94	77.21	74.63
D. alata SQS	68.98	71.71	71.74	73.51	74.94	100.00	78.68	78.36
P. polyphylla SQS	72.03	71.04	73.77	76.05	77.21	78.68	100.00	78.16
A. officinalis SQS	70.10	69.85	73.13	74.44	74.63	78.36	78.16	100.00

Squalene epoxidase

>*S._polyrhiza*_SQE
MEYVFGGVVASLLGFLLMFPFQGKSRKGGMTRNAEIKGRLSKSAECGSGEACEGSSDVIVVGAGVA
GSALACALGKDGRRVHVIERDLSEPDRIVGELLQPGGYLKLVELGLEDCVQEIDAQRVLGYALFKDG
KNTKLSYPLENFHGDVAGRSFHNGRFIQRMRGKAASIPNVRLEQGTVTSLIEENGVVRGVSYKTKIG
EELKVYAPLTIVCDGCFSNLRRSLCSPKVDVPSFFVGLVLENCQLPHPNHGHVILADPSPILFYPISSTE
VRCLVDVPGQKVPSIANGEMEIYLKTSVAPQLPEELREPFISAIDRGTIRTMPNKSMPAAPHPTPGALL
MGDAFNMRHPLTGGGMTVALSDIVVLRNLLKPLHNLHDAPALCKYLESFYTLRKPVASTINTLAGA
LYKVFSASPDRAMSEMRQACFDYLSLGGSFSAGPISLLSGLNPRPLSLVVHFFAVAIYGVGRLLIPFPSP
KRMWIGARLISGAANIILPIIKAEGVRQMFFPATVPAYYRAPPTR*

>*S._cereale*_SQE
MAAAAGVWQLVGAAAATLLAAALVAVALGRQRQRRRRRAPVEGIPAPVDGCAVADGEGSAAVDGPTD
VIIVGAGVAGSALAYTLGKDGRRVHVIERDLTEPDRIVGELLQPGGYLKLMELGLQDCVDEIDAQRVLGYA
LFKDGKNTKLSYPLEKFHSDVAGRSFHNGRFIQRMREKAASLPNVQLEQGTVTSLLEENGTVKGVQYKIKS
GEELKAYAPLTIVCDGCFSNLRRALCSPKVEVPSCFVGLVLENCELPHANHGHVILANPSPILFYPISSTEVR
CLVDVPGQKVPSIASGEMANYLKTVVAPQIPPQIYDSFIAAIDKGSIRTMPNRSMPAAPHPTPGALLMGDAF
NMRHPLTGGGMTVALSDIVVLRNLLKPLRNLHDASALCKYLESFYTLRKPVASTINTLAGALYKVFSSSPD
KARDEMRQACFDYLSLGGVCSNGPIALLSGLNPRPLSLVAHFFAVAIFGVGRLMLPLPSPKRMWTGARLIS
GACGIIFPIIKAEGVRQMFFPATVPAYYRAPPEAEF*

>*A._americanus*_SQE
MQVLYGAVAGSILLGFLGFCYALASSKREAKRASGGDARSEAVVDGSVDGRPRSVDDGATDVIVVGAGV
AGAAIAYILGKDGHRVRVIERDLSEPDRIVGELLQPGGYLKLIELGLQDCVEEIDAQRVLGYALFKDGRDTK
VSYPLENFSSDVSGRSFHNGRFIQRMREKAVSLPNVKLEQGTVTSLLEENGTVKGVVYRVKSGAEFKACAP
LTVVCDGCFSNLRKSLCHPNVDVPSYFVGLVLKNCELPYPNYGHVILANPSPILFYPISSTEIRCLVDVPGPK
LPSMANGEMANYLKTVVAPQVPPQLYDSFISAVESGSIRTMPNKSMPASPFPTPGALLMGDAFNMRHPLTG
GGMTVALSDIVVLRNLLKPLHDLNDSSSLCKYLESFYTLRKPVASTINTLAGALYKVFCASSDEARNEMRE
ACFDYLSLGGVFSNGPVALLSGLNPRPLSLVMHFFAVAIYGVGRLLLPFPSPKRLWIGARLISSASSIIFPIIKA
EGVRQMFFPATVPAYYRAPPTK*

>*D._alata*_SQE
MLAECLFGGIVAVLLWFLLSKKSKQRNAGSLAEIPDVLDASVDRGTDVIIVGAGVAGSALAYTLGKDGRR
VHVIERDLSEPDRIVGELLQPGGYLKLIELGLDDCVEEIDAQRVLGYALFKDGKNTKLSYPLEKYHSDVAG
RSFHNGRFIQRMREKAATLPNVRLEQGTVNALLEENGTVKGVAYKTRAGEELKAYAPLTIVCDGCFSNLR
RSLCSPKVDVPSCFVGLVLENCELPFPNHGHVVLADPSPILFYPISSTEVRCLVDVPGQKVPSIANGEMADYL
KTVVAPQIPKELYDSFIAAIDKGSMRTMPNKSMPANPHPTPGALLMGDAFNMRHPLTGGGMTVALADIVV
LRNLFKPLRNLHDASALCKYLEAFYTLRKPVASTINTLAGALYKVFCASPDQARNEMRQACFDYLSLGGV
CSTGPVALLSGLNPRPLSLVAHFFAVAIYGVGRLLVPFPSPKRLWIGARLISSASGIIFPIMRAEGFRQMFFPA
TIPAYYRAPPSD*

>*A._officinalis*_SQE
MLAIHPQLNPSPPPIPTRHRKYTPRNSRSSSSSSSSSSSYLSSEPIQKEGKGRICNLERERRGRSISTKERMLML
QCLFAGIVASLLGFLLLSKKKKQEKGSPRNGGSLEQVRGQCGGPDVIIVGAGVAGAALAYTLGKDGRQVH
VIERDLREPDRIVGELLQPGGYMKLIELGIADCVEKIDAQRILGYALFKDGKDAKLTYPLEKYHSDIAGRSF
HNGKFIQRMREKAATISNVRLEQGTVTTLLEDNGVVKGVSYKNEAGELRKTYAPLTIVCDGCFSNLRRSLC
TPKVDVPSCFVALILENCQLPYANHGHVVLANPSPILFYPISSAEVRCLVDVPGNKLPSITNGEMAKYLKTV
VAPQLPSQLYDAFILSVDRGNIKSMPNRSMPASPQPTPGALLLGDSFNMRHPLTGGGMTVALSDIVLLRDLI
NPLHDLHDAAAVCKYLESFYTLRKPVSSTINTLAGALYQVFSASSDQARIEMREACFDYLSLGGVFSNGPV
SLLSGLNPRPLSLVAHFFAVALYGVGRLLLPFPSFKQLWLGLRLISVASSIILPIIRAEGVRQMFFPATVPAYY
RSPPTHMNS*

>*C._nucifera*_SQE
MLAEYLFGGVISSFLGFLILLILRSAKEKNKRTSNAGTPRKTAEESRRQAAGDGGTDVIVVGAGVAGSSLAH
TLGKDGRQVHAIERDLTEPDRIVGEFLQPGGYLKLIELGLEDCVEEIDAQRVFGYALFKDGKNTRVSYPLK
KYHSDVSGRSFHNGRFIQRMREKAASLPNVKLEQGTVTALLEENGTVKGVLYKNKAGEEVQAYAPLTIVC
DGCFSNLRRTLCSPNVDIPSCFVGLILENCQLPYPNHGHVILADPSPILFYPISSTEIRCLVDVPGQKVPSVAN
GEMADYLKTVVAPQIPSELHDAFVSAVDQGSIRTMPNKSMPASPFPTPGALLMGDAFNMRHPLTGGGMTV
ALSDIVVLRDLLRPLRDLADASSLCKYLESFYTLRKPVASTINTLAGALYKVFSASPDRARNEMRQACFDY
LSLGGVFSSGPIALLSGLNPRPLSLVAHFFAVAIYGVGRLLLPFPSPRRLWIGARLISGASAIIFPIIRAEGFRQ
MFFPATVPACYRAPPTH*

>*Z._officinalis*_SQE
MADQYLLGTIMASILMFFLLVGFWGCKRKAHLAPGNRSSCGIRAEKGGTGTDVIVVGAGVAGSALAYTLG
KDGRRVHVIERDLSEPDRIVGELLQPGGYLKLLELGLEDCVGEIDAQRIVGYALFKDGKGTRLSYPLEKQH
ADVAGRSFHNGRFIQRMREKAASLANVQQEQGTVTSLIEENGTVKGVVYKTKSGEECKAFAPLTIVCDGC
FSNLRRRLCSPKVDVPSCFVGLVLENCLLPFPNHGHVILGDPSPILFYPISSTEVRCLVDIPGNKVPSVANGE
MANYLKTVVAPQVPIELHAAFIEAIDKGNIRSMPNRSMPAAPHPTPGALLLGDAFNMRHPLTGGGMTVALS
DIVVLRDLLRPLHDLHDASSLCKYLESFYTLRKPVASTINTLAGALYKVFSASPDRARIEMRQACFDYLSLG
GIYSSGPIALLSGLNPRPMSLVAHFFAVAVYGVGRLLLPFPSLKGLWIGARLISSASGIIFPIIKAEGVRQMFLP
ATVPAYYRVPPPS*

A. officinalis SQE	100.00	73.82	75.55	75.25	75.83	73.26	76.59
A. americanus SQE	73.82	100.00	76.22	75.92	78.64	77.44	78.22
Z. officinalis SQE	75.55	76.22	100.00	79.41	80.59	79.03	80.56
***S. polyrhiza* SQE**	**75.25**	**75.92**	**79.41**	**100.00**	**79.14**	**79.96**	**79.84**
C. nucifera SQE	75.83	78.64	80.59	79.14	100.00	78.27	82.87
S. cereale SQE	73.26	77.44	79.03	79.96	78.27	100.00	83.92
D. alata SQE	76.59	78.22	80.56	79.84	82.87	83.92	100.00

Cycloartenol synthase

>*S._polyrhiza*_CAS
MWRLRIAEGDGPWLRTVNGHVGRQVWTFDPEATPEELAEVERAREDFRIHRFERKHSADLLMRLQ FARENPLGAVLPRVEVAENEAVGEDAVATTLRRAMNFHSTIQARDGHWPGDYGGPNFLMPGLVITL YVSGALNTALSREHQAEIRRYLFNHQNEDGGWGLHIEGPSTMFCTTLTYVTLRLLGEGADGGEGAV AKGRQWILDHGGATACTSWGKLWLSVLGVYDWSGNNPLPPEIWLLPYFLPIHPGRMWCHCRMVY LPMSYLYAKRFVGPITPLVLALRKELYAVPYAELDWNKARNLCAKEDLYYPHPLIQDMLWTCLHKF VEPILMRWPGTLLREKALQTVMEHIHYEDENTRYICIGPVNKTLNMLCCWVEDPNSEAFKLHIDRVH DYLWVAEDGMKMQGYNGSQLWDTAFAVQAMVSTNLYEEYGESLKKAHDYIKATQVMNDCPGDLS AWYRHISKGAWPFSTADHGWPISDCTGEGLKVALLLSRLPAQLVGEQIDASRLYDAVNVILSLRNED GGFATYEQTRSYPWLEVINPAETFGDIVIDYTYVECTSASIQALEAFRKLYPGHRREEIGDCIAGAARF IEKIQKPDGSWYGSWAVCFTYGTWFGVKGLVAAGRTHESSAAVRRACDFLLSKQLPSGGWGESYLS CQDKVYTNLPGDRPHVVNTAWAMLTLIAAGQGERDPTPLHRAARALISAQMENGDFPQQEIMGVFN KNCMISYSAYRNIFPIWALGEYRARVLLARGG*

>*S._cereale*_CAS
MWRLRIGEAAGGEPWLRTTNAHAGRQVWEFDPTASAAAYNDVDAARRKFSSRRHELKHSADLLVRSQFA ELNPSKLDIPGIKLGLLGEHQDVTEETVLASLKRAIGRHVAIQAHDGHWPGDLAGPMFLLPGLVVALHSIG VLNTVLSSEHQKEIRRYLYNHQNNDGGWGLHIESPSTMFGSVLTYVTLRLLGEGPHDGDGTMEMARNWIL DHGGATFTATWGKFWLSVLGVFDWCGNNPLLPELWLLPYCLPFHPGRTWSHCRMVYLPMSYIYGKRFICP VTPVVLDLRNEIYTVPYEEIDWDKARNQCAKEDLYCPHPLGQDILWDTLHKFVEPVLSHWPGSKLRKKAL KNVMQHIHYEDENTQYICSGAVGKVLNMLCCWIEDPNSLKFKLHIPRIYDYLWIAEDGMKMQGYNGSQL WDTTFTVEALVATELIEEFGPTLKLAHNYIKNSQVLRDCHGDLSDWYRHISKGAWTFSTADQGWPVSDCT ALGLKTSLLLSKISPKIVGESLESERLYDAVNCLLSWMNRNGGFAPYELTRSYAWLEFLNPSEVFEDIMIDR PVVECTSEVLQALTSFKKHYPAHRSEEIDRCIHKAGDFIQSLQRSDGSWYGSWAVCFTHGTYYGVRGLVA AGRTFESSPAIRKACEFLLSKELPSGGWGESYLSCKDKVYTELEGRRPHVVNTSWAMLALIDAGQAERDPT PLHRAAKVLINLQLEDGEFPQQEIIGVFARNCTITYSNYRNIFPIWALGEYRGRVLGAQSPGERGNAS*

>*A._americanus*_CAS
MWKLKFGEGGGDPWLRTLNGHVGRQVWEFDPSLGSPEELAEVELARDGFRNLRFQKKHSSDLLMRLQFA KENPLRSIRPQVILGESEEEVTEEAVTTTLRRAIDFHSTIQAHDGHWTGDYGGPMFLMPGLVIALYITGALNT VLSAEHRREMCRYLYNHQNEDGGWGLHIEGHSTMFGTGLTYVTLRLLGEGANGGDGAMEKGRKWILDH GSITHITSWGKMWLSVLGVFDWSGNNPLPPETWLLPYFIPLHPGRMWCHCRMVYLPMSYLYGKRFVGPIT ETVISLRTELYTLPYDQVDWNEARSLCAKEDLYYPHPVIQDILWGTLHKVVEPVLMRWPGSKLREKALQT TMQHIHYEDENTRYICIGPVNKVLNMLCCWVEDPNSEAFKLHLPRIYDYLWVAEDGMKMQGYNGSQLW DTAFAVQAVISTNLSEEYGPVLEKAHSYIKKSQVLNDCPGDLSFWYRHISRGAWPFSTADHGWPISDCTSE GLKASLLLSQMSSESVGEPLSTENFYDAVNVILSLQNEDGGFATYELSRSYPWLELINPAETFGDIVIDYPCV ECTSAAVQGLAAFKKLYPKHRKDEMESCIAKAAKYIENIQLSDGSWYGSWAVCFTYAAWFGVKALVAAG KTYQSSSHIRKACDFLLSKQLASGGWGESYLSCQDKVYTNLKGDRSHVVNTGWAMLALIDADQAKRDPV PLHRAARVLINSQMENGDFPQQEIMGVFNRNCMISYSAYRNIFPIWALGEYRCRVLLAS*

>*D._alata*_CAS
MWRLKIAEGGNPWLRTVNNHVGRQVWEFDPNLGSPEELAEVEKAREAFHQHRFEMKHSSDLLMRFQFAK ENPLELTLPQVKVEDNEDVTEEAVTTALRKAISRHSTLQAHDGHWPGDYGGPMFLMPGLVIALYVTGALN AVLSPQHQQEICRYLYNHQNKDGGWGLHIEGHSTMFGSVLTYITLRLLGEEAEGGDGSMQRGRKWILDHG GATLITSWGKFWLSVLGVFDWSGNNPLPPEIWLLPYFLPIHPGRMWCHCRMVYLPMSYIYGKRFVGPITPL VLSLRKELYNLSYDRIDWNLARNQCAKEDLYYPHPLLQDILWASLHKFAEPVLLHWPGSKLREKALRTTM QHIHYEDENTRYICIGPVNKVLNMLCCWVEDPNSEAFKLHLPRIYDYLWVAEDGMKMQGYNGSQLWDTA

FTVQAIVATNLSEEFAHPLKKAHDYIRKTQVLDDCPGDLSFWYRHISKGAWPFSTADHGWPISDCTAEGLK
ASLLLSRFSPEIVGEPVDAKRLYDAVNVILSLMNEDGGFATYELTRSYAWMEIINPAETFGDIVIDYQYVEC
TSASIQALTAFKKLYPGHRREEIEACIQKAVNFIEKTQKPDGSWYGSWAVCFTYGIWFGTLGLIAGGRTYQ
NSSCVRKACDFLLSKELPSGGWGESYLSCQDRVYTNLEGNRPHAVNTGWAMLTLIEAGQAERDPKPLHRA
AKVLINMQLENGEFPQQEIMGVFNKNCMISYSAYRNIFPIWALGAYRCRVLPYLNH*

>*A._officinalis*_CAS
MWKLNIGEGGPCLKSRYDFIGRQVWEFDPKLGSLEEREAVERARQEFRRHRFQKKQASDLLMRMQFAKE
NNHDDTNLPQVKLKEDQEISEEAVRTTLRRAIGYFSTIQAHDGHWPADFPGPLFLTPTLVIALYVTGAVNSA
FSAEHQEELCRYIYNHQNEDGGWGFHTEGLSVMFSTALCYTALRLLGEEVDYNEDGAMVKGRNWIHDHG
GVTAIPTWGKMWLSVLGVFEWSGVNPIPPELFIFPTILPIHPGRFWCHFRLAYLPMSYLYGKKFVGPVTSTV
LSLREELHNHPYHDIDWNLARNLCAKEDLYYPHPLAQDVLWECLHRIGEPLLRRWPLCKLRERALQAIME
YIHYEDENSVYICVGAAQKVLCMLCCWVEDPSSDSFKLHLARIPDYLWTAEDGMKMQGCGSQLWDAVL
AVQAILSSMLVEEYGTTLMKAHEFIKLSQILENPSGDFRRRHRHISKGGWAFTIPDHGWPVSDCTAEALKA
ALLLSRISPNIVGQPMATEQLNNALNIILSLQNRNGGFSTWELTRTYQWIEFFNASEFFADIVLEYQYVECTS
SAIQALALFKEIYPGYRTEEVESCIQRGMKFIENKQEDDGSWYGSWGICYTYGTWFGVEGLVASGKTYDTS
SSIRRACHFLLSKQLASGGWGESYLSSKNKVYTNLDGNCSHLVNTAWAMLALVKSGQVARDPAPLHRAA
KFLINTQQENGDFPQQEMLGSLGKNGVLNYASYRNIFPIWALGEYRKHLLWNSNTSAVDNIRV*

>*C._nucifera*_CAS
MWRLKIAQGGSPWLRTTNNHVGRQLWEFEPNLGTAEEIAAVEKARELFREHRFEMKHGSDLLMRLQFAK
ENPLEINFPHIKLEDHEDVTEEVVLTSLRRAISCYSTLQAHDGHWPGDYGGPMFLMPGLIIALSVTGALNTV
LSPEHKQEIRRYLYNHQNKDGGWGLHIEGHSTMFGSALTYVTLRLLGEGPDDGDGAMQKARTWILDHGG
VTTITSWGKFWLSVLGVFEWSGNNPLPPEMWLLPYILPVHPGRMWCHCRMVYLPMSYIYGKRFVGPITPT
VLSLRKELFACPYQQIDWNQARNECAKEDLYYPHPLIQDIIWASLHKVVEPVLMHSPGSKLREKALHTAM
QHIHYEDENTRYICIGPVNKVLNMLSCWIEDPNSEAFKLHLPRIYDYLWLAEDGMKMQGYNGSQLWDTAF
TVQAIISTDLSEEYGPTLKRAHDYVKNSQVLEDCPGDLRFSYRHISKGAWPFSTADHGWPISDCTAEGLKAS
LLLSKISPEIVGEPLCAGRIYDAVNVILSLMNKDGGFATYELTRSYAWLEIINPAETFGDIIIDYPYVECTSAAI
QALTSFKKLYPGHRRDDIENCIRKAANFIEMIQQPDGSWYGSWAVCFTYGTWFGVKGLIATGRTYQNSPCI
RKACQFLLSKQLASGGWGESYLSCQNKVYSNLEGDRCHAVNTAWAMLALIDAGQGERDPEPLHRAVKVL
INMQMESGEFPQQEIMGVFNRNCMISYSQYRNIFPIWALGEYRCRVLLTKKN*

>*Z._officinalis*_CAS
MWRLKIAEGGSPWLRTTNNHVGRQVWEFDPNLGTPEEIEEVERVREAFRESRFEKKHSSDLLMRLQFAKE
NPLDMDDSIIRIQDDENVTEATLETVLRKAISRISTLQAHDGHWAGDYGGPMFLMPGLIITLHVTGALNTVL
TPEHQKEICRYLYNHQNRDGGWGLHIEGDSTMFGSVLTYVILRLLGQGPDDGDGAMEKGRKWILDHGAA
TCITSWGKMWLSVLGVFDWSGNNPLPPEMWLLPYILPVHPGRMWCHCRMVYLPMSYIYGKRFTGPITPIV
LSLRKEIFNCPYDQIDWNQARNECAKEDLYYPHPFIQDILWASLHKIVEPILMHWPGSLLREKAISTAIQHIH
YEDENTRYICIGPVNKVLNMLCCWIEDPNSEAFKLHLPRIYDYLWVAEDGMKMQGYNGSQLWDTAFTVQ
AIMSSNLSEEFGVTIKKAHEYIKFSQVLEDCPGDLDFWYRHISKGSWPFSTADHGWPISDCTSEALKAALLL
SKISPEVVGDPIEARKLYDAVNVILSLMNKDGGFATYELTRSYAWLEIINPAETFGDIVIDYPYVECTSAAIQ
ALTSFKKLYPGHRRGEIDNCINKSACFLEKIQKNDGSWYGSWGVCFTYATWFGVMGLISAGRTYERSSCIR
KACKFLLSKQLASGGWGESYLSCQDKVYTNLEGNRIHAVNTGWAMLALIAAGQGERDPKPLHKAAKTLI
NMQMENGEFPQQEIMGVFNKNCMISYSAYRNIFPIWALGEYRTRVLCPKDH*

A. officinalis CAS	100.00	56.75	61.32	64.06	62.70	62.43	61.90
S. cereale CAS	56.75	100.00	67.37	68.21	70.84	71.37	69.00
***S. polyrhiza* CAS**	**61.32**	**67.37**	**100.00**	**77.38**	**78.50**	**74.93**	**75.07**
A. americanus CAS	64.06	68.21	77.38	100.00	79.39	78.60	77.01
D. alata CAS	62.70	70.84	78.50	79.39	100.00	82.35	81.03
C. nucifera CAS	62.43	71.37	74.93	78.60	82.35	100.00	83.14
Z. officinalis CAS	61.90	69.00	75.07	77.01	81.03	83.14	100.00

Sterol methyltransferase

>*S._polyrhiza*_SMT
MSRAGALDLGSGVGGKIEKKDVQSAVEQYEKYHTCFGGDEEARKKNYTDMVNKYYDLATSFYEFG
WGESFHFAHRWNGESLRESIKRHEHFLALQLGLKRGMKVLDVGCGIGGPLREIARFSSTSVTGLNNN
DYQISRGEELNRLAGLEKSCNFVKADFMKMPFDEDTFDAIYAIEATCHAPDAVGCYKEIYRVLKPGQ
YFAAYEWCMTESFDPRNENHQKIKAEIELGNGLPDIRSTAQCLDALKRAGFEVIWEKDLAMDSPVP
WYLPLDTSRFSLTSFRLTAVGRFLTRTLVKTLEAVGFAPAGSQRVSSFLEKAAEGLVEGGRKEVFTP
MYFFLVQKPRQ*

>*S._cereale*_SMT
MSKSGALDLASGLGGKIDKEQVKSAVDEYEKYHGYYGGKEESRKSNYTDMVNKYYDLATSFYEYGWGE
SFHFAHRWNGESLRESIKRHEHFLALQLELKPGMKVLDVGCGIGGPLREIARFSSTSVTGLNNNDYQITRGK
ALNRSVGLGATCDFVKADFMKMPFSDNTFDAVYAIEATCHAPDPVGCYKEIYRVLKPGQCFAVYEWCITD
HYDPNNATHKRIKDEIELGNGLPDIRSTRQCLQAVKDAGFEVIWDKDLAEDSPLPWYLPLDPSRFSLSSFRL
TTVGRIITRNMVKVLEYVGLAPEGSQRVSSFLEKAAEGLVEGGKKEIFTPMYFFVVRKPLSE*

>*A._americanus*_SMT
MSGAIDLASGLGGKIEKKEVRSAVDQYEKYHGHFGGDEEARKANYTDMVNKYYDLSTSFYEFGWGESFH
FAHRWNGESLRESIKRHEHFLALQLGLKPGMKVLDVGCGIGGPLREIARFSSTSVTGLNNNDYQISRGKEL
NHLAGVHKTCDFLKADFMKMPIADNTFDAIYAIEATCHAPDALGCYKEIFRVLKPGQHFAAYEWCMTDAF
DPNNQDHQRIKAEIELGDGLPDIRTTRKCLEALKLAGFEVIWEADLASESPVPWYLPLDTSHFSLSSFRLTSV
GRFITKNLVRTLEFIGLAPEGSQRVSSFLEKAADGLVEGGRKEIFTPMYFFLARKPLSES*

>*D._alata*_SMT
MAKSGALDLATGVGGKINKKEVQSAVEQYEKYHVCFGGDEETRKTNYSDMVNKYYDLATSFYEFGWGE
SFHFAHRWVGESLRESIKRHEHFLALQLGLKRGMKVLDVGCGIGGPLREIARFSSTSITGLNNNEYQISRGIE
LNRVAGLENTCDFVKGDFMKMSFPDNTYDAVYAIEATCHAPDALGCYKEIYRVLKPGQCFAAYEWCMTD
HYDPNNETHKKIKAEIELGNGLPDVRSTAQCLEALKQAGFEVIWEKDLAADSPVTWYLPLDTSRFSITSFRL
TAFGRLITRTMVKTLEYVGLAPAGSERVSSFLEKAAEGLVEGGRKEIFTPMYFFLVRKPLSDS*

>*A._officinalis*_SMT
MGPRHFAAYEDSVFRHYVQFGPPGVDLPTFEHILSQWDKKSKKFVFTDRNDGEEIPCSFNLEWVMEHTWF
SNEGLHVDHNRDRKNRIWSRFFDKPEVGGSSSIAPPKEGPTRRNVEDLLLKLLWYYELTDIKKPALGCTGP
AAVDAGRARGPRDLLHKKKRRAEDDDIPIDALDIDEDAEQPNDAVEQMDWPQQVIYWKSIAAMNESLHR
ENENKFKDTPGTVGGINVQYEGDDNKSLLFDEANKESEAPPTGRASPQPLFSDIHKESEDAPPGELPPPEAS
AVDKALEAEINEGEKVSRPAKELLTCTSTQCEGTGREITPAVEVPSAGDGKTQTCIDRRGYVFLIFILRMGCL
ESNTLREDTGMEIKQSENAPAPAIETSTDDEDAPIVFSRALNKRKRGATGARGKVAAAAPPKRKRCKRSVK
PSKWVVTPYTEGKKKKEKDNIGDKALIEVAGEEEAQVQEKVAELAPARLQSDPHSIWEKHMSSPMSSEEE
MYRDKLNEGIPFPDGFIIKFLGDKDTFVHNYKLTMSKTGALDLASGLGGKINKKEVESAVEQYEKYHVYH
GGDEETRKTNYSDMVNKYYDLATSFYEFGWGESFHFAPRWKGESLRESIKRHEHFLALQLGLKSGMKVL
DVGCGIGGPLREIARFSSTSITGLNNNEYQISRGVELNKESGLAENCNFVKADFMKMPFTDSTFDAIYAIEAT
CHAPDALGCYKEIYRVLKPGQCFAAYEWCMTDHFNPENEVHQKIKAEIELGNGLPDVRSTRQCLEALKLA
GFEVIWEKDLAVDSPLPWYLPLDTSVISITSFRLTSFGRFLTKSMVKALEFVRLAPAGSDRVSSFLEKAAEGL
VEGGRKEIFTPMYFFLVRKPHSAS*

>*C._nucifera*_SMT
MSKSGALDLASGVGGKIDKKEVKSAVEQYEKYHIYFGGDEETRKANYSDMVNKYYDLATSFYEFGWGES
FHFAPRWKGESLKESIKRHEHFLALQLGLKRGMKVLDVGCGIGGPLREIARFSSASITGLNNNEYQISRGKE
LNQLVGLSESCNFVKADFMKMPFSDSTFDAIYAIEATCHAPDVLGCYKEIYRVLKPGQCFAAYEWCMTDH
FDPTNESHQKTKAEIELGNGLPDIRTTNQCLEALRLAGFEVVWEKDLASDSPVPWYLPLDTRHFSITSFRLT
AFGRLITRTMVKTLEFVGLAPKGSERVSSFLEKAAEGLVEGGRKEIFTPLYFFLARKPL*

>*Z._officinalis*_SMT
MSKSGALNLVSGVGGKINKEDVQSAVEQYEKYHTSYGGDEESRKANYSDMVNKYYDLATSFYEFGWGES
FHFAHRWKEESLRESIKRHEHFLGLQLGLKPGMKVLDVGCGIGGPLREIARFSSTSITGLNNNEYQISRGSAL
NRIAGLDKSCDFVKADFMKMPFSDDTFDAIYAIEATCHAPDAVGCYKEIYRVLKPGQYFAAYEWCITDHF
NPNNESHKKIKAEIELGNGLPDVRSTRECLDALKLAGFEVTWEKDLAADSPLPWYLPLDTSRFSITSFRLTA
FGRFITRTMVKTLEFLRIAPEGSERVSAFLEKAAEGLVAGGRMEIFTPMYFFLVRKPL*

S. cereale SMT	100.00	81.29	79.88	81.29	81.98	79.36	79.82
A. americanus SMT	81.29	100.00	82.70	79.41	81.05	81.05	82.35
***S. polyrhiza* SMT**	**79.88**	**82.70**	**100.00**	**84.21**	**85.13**	**83.09**	**83.63**
Z. officinalis SMT	81.29	79.41	84.21	100.00	85.67	84.80	84.21
D. alata SMT	81.98	81.05	85.13	85.67	100.00	86.38	86.84
A. officinalis SMT	79.36	81.05	83.09	84.80	86.38	100.00	87.13
C. nucifera SMT	79.82	82.35	83.63	84.21	86.84	87.13	100.00

C8,7 sterol isomerase

>*S._polyrhiza*_HYD1
**MAGGYGHPYVPRDLKLPEFVPNFLTTGDILGAFVTGSLLAVLLVWAFSGRYPKLSKTDRILMCWWA
FTGLIHCIIEGYFAFSPRFFQKKTPFYLAEIWKEYSKGDSRYAARDPTIVTVEGITAVLEGPASLLAVLA
IAWQRPYQHTIQLAVCLGQLYGCLVYFITAHLLGHGFSVNAVYFWAYFVGANSPWVVIPTLIAVRSW
RAISAATAAVSPAAATSKKSR***

>*S._cereale*_HYD1
MGAHPYVPASLDLPGYVPLRLTQLEILGAYLGTSLFVVVAVWLLSGRCRRLSGVDRLLMCWWAFTGLTHI
LIEGPFVFTPDFFTKTNPNFFDEVWKEYSKGDSRYVARDTATVTVEGITAVLKGPASLLAVYAIASRKSYSH
ILQFAVCLGQLYGCIVYFTTAYLDGFNFWASPFYFWAYFIGANSSWIVIPLLIATRSWKKICAAVHQSEKVK
TK*

>*A._americanus*_HYD1
MSSAMEMEYPHPYVPTDLRLPGYVPNFLTQTEIVVPYIACSVVVASLVWFFSGRISKISKTDRILMCWWVF
TGLTHIILEGYFVFSPEFYKKKVPCYLAEIWKEYSKGDSRYAGRDTAVVTVEGVTSVLEGPASLVAMYAIA
TGKPYSHTLQLTICIGQLYGCIIYFVASFLDGDNFAKSPFYYWIYYVFANSIWVVIPTVIVVRSWRKISKAFL
GENKKRKAH*

>*D._alata*_HYD1
MASFEHPYVPKDLELHGYVPCFLSQSEIVAPYLVVSVVVVALVWILSGRVSKISGIDRLLMCWWAFTGLTH
IILEGYFAFSPDFYKEKTPFFLAEVWKEYSKGDSRYAGRDSAIVTVEAVTAVIEGPACLLAIYAIASKKAYSY
TLQLAICLGQLYGCLIYFITAFLEGDNFSASPFYYWTYYIGANGWWVLIPSLIAIRSWKKINAAFQGRTKKT
KAN*

>*A._officinalis*_HYD1
MPAVVTVEGITAAIEGPACLLAVYAIATRKPYNYTLQLAISLGQLYGCLVYFITAFLLGDNFSTSPYYYWAY
YIGANSSWVVIPSLIVMRSWKKISSAVHAEKKMKTG*

>*C._nucifera*_HYD1
MATAFEHPYVPRDLHLPGYIPCFLSQKDIVVPYLGTSIVGVALIWLFSGRLSKISKTDRLLMCWWAFTGL
THIIVEGYFAFSPEFYKEKTPHFLAEVWKEYSKGDSRYVARDAGVVTVEGITAVLEGPASLVAVICCMES
AYLGASA*

>*Z._officinalis*_HYD1
MAAREGGEWAHPYVPKDLKLPGYVPCFLSQKDIVVPYLGTSLLLVSFIWIFSGRSKLTKSDRLLMCWWAF
TGLTHIILEGYFAFSPEFYKKKTPHYLAEVWKEYSKGDSRYVGRDAGIVTVEGITAVLEGPASLIAVYAIAT
RKPYSYILQFAVCLGQLYGCLVYFITAFLEGDNFATSPFYYWVYYYIGANGWWVVIPLLIANRSWKKITAAI
QAEKKIKTG*

***S. polyrhiza* HYD1**	**100.00**	**59.72**	**60.00**	**57.99**	**61.22**	**62.04**	**63.76**
S. cereale HYD1	59.72	100.00	57.87	62.50	58.33	66.67	67.44
A. americanus HYD1	60.00	57.87	100.00	68.04	65.31	62.04	66.22
D. alata HYD1	57.99	62.50	68.04	100.00	67.81	69.44	73.27
C. nucifera HYD1	61.22	58.33	65.31	67.81	100.00	44.44	74.66
A. officinalis HYD1	62.04	66.67	62.04	69.44	44.44	100.00	75.00
Z. officinalis HYD1	63.76	67.44	66.22	73.27	74.66	75.00	100.00

Δ^7 sterol-C5 desaturase

>*A._thaliana*_DWF7
MAADNAYLMQFVDETSFYNRIVLSHLLPANLWEPLPHFLQTWLRNYLAGTLLYFISGFLWCFYIYYLKIN
VYLPKDAIPTIKAMRLQMFVAMKAMPWYTLLPTVSESMIERGWTKCFASIGEFGWILYFVYIAIYLVFVE
FGIYWMHRELHDIKPLYKYLHATHHIYNKQNTLSPFAGLAFHPVDGILQAVPHVIALFIVPIHFTTHIGLLFM
EAIWTANIHDCIHGNIWPVMGAGYHTIHHTTYKHNYGHYTIWMDWMFGSLRDPLLEEDDNKDSFKKAE*

>*S._polyrhiza*_DWF7
MAGRGVEDYLQLFVDEGDWYDRIVLGTLLPGWAWESLPRPARSWLRNYIGGVLLYFGSSLLWCLY
FYHWKRNIYLPKDAIPSNKAMLLQIAVAMKAMPWYCCLPTLSEYMIQRGWTQCFSAISDVGWALYV
VYLSIYLVICEFGIYWMHRELHDIKPLYKYLHATHHIYNKQNTLSPFAGLAFHPLDGILQAVPHVIAL
FLVPTHFMTHVLLLFCEGVWTANIHDCVHGDVWPVMGAGYHTIHHTTYRHNYGHYTIWMDWMFG
TLRYPEEDMKKAD*

>*S._cereale*_DWF7
MAAAAAEQEDYWGLFREETDWYNEIFLSAVVPGGGGWWRALPHPLRSWLRNCIGGYLLYFATGFLWCF
VIYYWKRNAYIPKDAVPTVEAMKKQIIVASKAMPFYCALPSVSEHMIESGWTRCFFHISEVGWPMYIVYVS
LYLIFVEFGIYWMHRELHDIKPLYKHLHATHHIYNKENTLSPFAGLAFHPLDGILQAISHVIALFLLPMHFRT
HIALLFIEAVWTANIHDCIHGKVWPVMGAGYHTIHHTTYRHNYGHYTVWMDWLFGTLREPEDLLKKD*

>*A._americanus*_DWF7
MGGPSAEYLGLFEAETDLYNRIVLGTLLPGSVWEPLPRVLQSWLRNYLAGTLLYLVSGFLWCFFIYYLRRN
AYLPKDCIPSKQAMSLQIMVAMKAMPWYCALPTVTEFVVEKGWTKCFSSIHEVGWTLYLIYLTIYMALVE
FGIYWMHRELHDIKPLYKYLHTTHHIYNKQNTLSPFAGLAFHPLDGILQAIPHTISLFIVPTHFMTHMLLLFC
EGVWTANIHDCIHGKIWPVMGAGYHTIHHTTYKHNYGHYTIWMDWMFGTLRDPEEDLKKA*

>*D._alata*_DWF7
MGSSDEYLQRFVAETDWYNSIVLGNLLPDSVWKPLPRPFQIWLRDYIGGTALYFISGFLWCFYIYYLKRNV
YLPKDAIPSNKAMLLQIIVAMKAMPWYCALPTLSEYMVEKGWTRCFSSISEVGWPAYIAYTAAYLVFCEF
GIYWAHRELHDIKPLYKHLHATHHIYNKQNTLSPFAGLAFHPLDGILQAISHVIALFIIPTQFTTHIALLFLEA
VWTANIHDCIHAKLWPVMGAGYHTIHHTTYRHNYGHYTIWMDWMFGTLRDPEEDLKKDE*

>*A._officinalis*_DWF7
MKTLDSLANSALSRPPSLPQNHSAIPSPPGELTAMERADYLHNFVEETDWYNDIVLTALLPDWAWQPLPRP
IRAWLRNYIGGTLLYFVSGFLWCFYIYYLKRNVYVPKDAIPSNHAMLLQIKVAMKAMPWYCALPTLSEFM
VESGWTRCYSSVSEVGWPLYIIYLVIYLVTCEFGIYWMHRELHDIKPLYKHLHATHHIYNKQNTLSPFAGL
AFHPLDGILQAVPHVICLFLVPTHFMTHIALLFIEAVWTANIHDCIHGKIWPVMGAGYHTIHHTTYRHNYGH
YTIWMDWMFGTLRDPEEDVNKVK*

>*C._nucifera*_DWF7
MEGADYLHRFVEEADWYNGIVLDALLPGGSWKRLPRPLQSWLRNYLGGTILYFVSGFLWCFYIYHWKRN
VYVPKDAIPSNKAMLLQILVAMKAMPWYCALPTLSEYMVERGWTRCFSSITEVGWPAYIAYATLYMVFV
EFGIYWMHRKLHDIKPLYKYLHATHHIYNKQNTLSPFAGLAFHPLDGILQAVPHVIALFLIPTQLTTHVLLL
FVEAVWTANIHDCIHGKVWPVMGAGYHTIHHTTYRHNYGHYTIWMDWMFGTLRDPEEDPKKAE*

>*Z._officinalis*_DWF7
MTNASSPEYLRQFVEETDWYNEILLGLIIPGDAWKRLPRPIQSWLRNYIGGTAVYFISGFLWCFYIYYWK
RHVYLPKDAIPSNKAMFLQIIVTMKAMPLYCGLPTISEYMVERGWTRCYSSIGEVGWLAHTVYLMIYLAL
VEFGIYWMHRELHDIKPLYKWFHTVHHIYNKQNTLSPFAGLAFHPLDGILQAVPHVIALFLVPTHFLTHM
ALLFCEAVWTANIHDCIHGKVWPIMGAGYHTIHHTTYRHNYGHYTIWMDWMFGTLRDPEEEFIKAK*

S. cereale DWF7	100.00	68.25	70.18	70.18	73.65	73.26	73.90
A. americanus DWF7	68.25	100.00	74.09	74.82	77.01	77.29	76.84
Z. officinalis DWF7	70.18	74.09	100.00	74.64	77.90	78.10	78.39
***S. polyrhiza* DWF7**	**70.18**	**74.82**	**74.64**	**100.00**	**77.90**	**75.55**	**78.75**
A. officinalis DWF7	73.65	77.01	77.90	77.90	100.00	82.12	82.78
D. alata DWF7	73.26	77.29	78.10	75.55	82.12	100.00	83.15
C. nucifera DWF7	73.90	76.84	78.39	78.75	82.78	83.15	100.00

Δ^7reductase

>*A._thaliana*_DWF5
MAETVHSPIVTYASMLSLLAFCPPFVILLWYTMVHQDGSVTQTFGFFWENGVQGLINIWPRPTLIAWKIIFC
YGAFEAILQLLLPGKRVEGPISPAGNRPVYKANGLAAYFVTLATYLGLWWFGIFNPAIVYDHLGEIFSALIF
GSFIFCVLLYIKGHVAPSSSDSGSCGNLIIDFYWGMELYPRIGKSFDIKVFTNCRFGMMSWAVLAVTYCIKQ
YEINGKVSDSMLVNTILMLVYVTKFFWWEAGYWNTMDIAHDRAGFYICWGCLVWVPSVYTSPGMYLVN
HPVELGTQLAIYILVAGILCIYINYDCDRQRQEFRRTNGKCLVWGRAPSKIVASYTTTSGETKTSLLLTSGW
WGLARHFHYVPEILSAFFWTVPALFDNFLAYFYVIFLTLLLFDRAKRDDDRCRSKYGKYWKLYCEKVKYR
IIPGIY*

>*S._polyrhiza*_DWF5
**MGTAEKSTVHSMLITLASMISLLALCPPFVILLWYTMVHADGSIALSWEYFKQHGFQGLKNIWPSPS
LTAWKIIFSYGFFEAVLQLALPGKRVEGPVSPNGNIPVYKANGVQAYVVTLITYLSLWWFGIFNPAIV
YDHLGEIYSALIVGSLVFCVLLHIKVRGYLAPSSTDSGSSGNLIIDFYWGMELYPRIGKSFDIKVFTNCR
FGMMSWAVLSLTYCIKQYEEIGHVSDSMLVNTILMLVYVSKFFWWESGYWSTMDIAHDRAGFYICW
GCLVWVPSVYTSPGMYLVNHPVNLGLSLALSILAAGILCIYINYDCDWQRQHFRKTNGKCSIWGRTP
SKIVASYRTTTGETKTSLLLTSGWWGLARHFHYVPEILASFFWTAPALFSHCLPYFYVVYLTILLADR
AKRDDDRCSTKYGKYWKMYCQRVPYRIVPGVY***

>*S._cereale*_DWF5
MAKQKQQQQPKPAPAAPPARPSKTAHSPWFTYASMVTLFSLCPPFVILLWYTMVHADGSVLRTYEHLRDH
GILDGLKAIWPVPTLVAGKIILGFALFEAALQLLLPGKRFEGPISPSGNVPVYKANGLLAYAVTLLTYLSLW
WFGIFNPAIVYDHLGEIYSALVFGSLVFCLLLYIKGHVAPSSSDSGSTGNVIIDFYWGMELYPRIGKHFDIKV
FTNCRFGMMSWAVLAVTYCIKQYEMNGQVADSMLVNTALMLIYITKFFWWESGYWCTMDIAHDRAGFY
ICWGCLVWVPSTYTSPGMYLVNHPVNLGPQLALSILLAGILCIYINYDCDRQRQEFRRTNGKASVWGKAPS
KIVASYQTTKGETKTSLLLTSGWWGFSRHFHYVPEILAAFFWTVPALFNHFMPYFYVIHLTILLLDRAKRDD
DRCSTKYGKYWKMYCNRVPYRVVPGIY*

>*A._americanus*_DWF5
MGGADEEKKKTVHSPMVTYASMLSLLTLCPPFVILLWYTMVHADGSVVQTWEYFQQQGLVQGFRSIWPK
PTAVAWKIIACFAAFEAALQLILPGERVEGPISPAGNRPVYKANGLAAYAVTLITYLGLWWFGIFNPAIVYD
HLGEIYSALIVGSLIFCVFLYIKGHLAPSSTDSGSTGNIIIDFYWGMELYPRIGKSFDIKVFTNCRFGMMSWAV
LTLTYCIKQYEENGKVADSMLVNTALTLVYVTKFFWWEAGYWNTMDIAHDRAGFYICWGCLVWVPSIYT
SPGMYLVSHPVNLGTQLALFILVAGVLCVYVNYDCDRQRQEFRRTNGKCLIWGKAPSKIEATYRTMKGET
KSSLLLTSGWWGLARHFHYVPEILAAFFWTVPALFNHFLPYFYVIFLTILLVDRAKRDDDRCQSKYQKYW
KQYCAKVPYRIIPGIY*

>*D._alata*_DWF5
MISLLLSLCPPFVILLWYTMVHADGSVMQTFEYLKQNGLEGLKTIWPSPSLIAWKIIAVFGVFEAFLQLALPG
KRFEGPVSPTGHVPVYKANGLQAYAVTLITYLGLWWFGIFNPAIVYDHLGEIYSALVTGSLVFCVFLYIKG
HLAPSSSDSGSSGNVIIDFYWGMELYPRIGKHFDIKVFTNCRFGMMSWAVLALTYCIKQYEQNGRVADSM
LVNTALMLVYITKFFWWESGYWCTMDIAHDRAGFYICWGCLVWVPSIYTSPGMYLVNHPVNLGTQLALSI
LAAGLLCIYINYDCDRQRQEFRRTNGKCKIWGKAPSKIVASYKTTKGETKTSLLLTSGWWGLARHFHYVP
EISAAFFWTVPALFSHFLPYFYVIFLTILLFDRAKRDDDRCSSK*

>*A._officinalis*_DWF5
MRSTPSMLRDPRPIGSPSCAAGVVRMIRSTVMGALTEEEAISFETVRGWPSSAYFVEGSFANIYIPLHFGMIE
GGKADRRLMQITGKALDFASILVFSDSLNICDKWYTMVHADGSILQTLGYLKQHGLQGFVDIWPTPSPTA
WKLIAAFGAFEAFLQLALPGKRVEGPISPTGQIPVYKANGLQAYAVTLITYISLWWFGIFDPAIVYDHLGEIL
AALTVGSLFFCVFLYLKGHMAPSSTDSGSSGNLIIDFYWGMELYPRIGKNFDIKVLTNCRFGMMSWAVLAV
TYCIKQYEMNGRVADSMLVNTVLMLVYITKFFWWEAGYWCTMDIAHDRAGFYICWGCLVWVPSVYTSP
GMYLVNHPVHLGNELALSILVAGLLCIYINYDCDRQRQEFRRTNGKCLIWGKAPSKIVASYTTTKGETKTS
LLLTSGWWGLARHFHYAPEIAAAFCWSVPALFNHFIPYFYVMFLTILLVDRAKRDDDRCSSKYKKYWKM
YCDKVPYRILPGIY*

>*C._nucifera*_DWF5
MGESKTVHSALVTYISMISLLTLCPPFVILLWYTMVHADGSVLQTYEYLKQHGLQGFKNIWPLPSAVACK
IIGCFGAFEAFLQLALPGKRVEGPVSPSGNKPVYKANGLQAYVVTLITYVSIWWFGVFDPAVVYDHLGEI
YAALIVGSFVFCIFLYIKGHLAPSSADSGTLGNVVIDFYWGMELYPRIGKSFDIKVFTNCRFGMMSWAVL
AVTYCIKQYEENGLVADSMLVNTVLMLVYITKFFWWEAGYWCTMDIAHDRAGFYICWGCLVWVPSVYT
SPGVYLVTHPVNLGTQLALSILVAGLLCVYINYDCDRQRQEFRRTNGKCLVWGKAPSKIIASYWTTNGETK
SSLLLTSGWWGLSRHFHYVPEILAAFYWTVPALFNHFLPYFYLLFLTILLFDRAKRDDDRCSSKYGKYWK
MYCEKVPYRIIPGIY*

>*Z._officinalis*_DWF5
MGETKTVHSPIVTYTSIFSLLTLCPPFVILLWYTMVHADGSVLQTFEYFRKHGLQGLKNIWPAPSLIAWKIIA
CFGAFEAILQLALPGKRVEGPISPNGNIPVYKANGLLAYAVTLVTYLGLWWFGIFNPAVVYDHLGEIYSALI
VGSLVFCILLYLKGHLAPSSSDSGSLGNFIIDFYWGMELYPRIGKNFDIKVFTNCRFGMMSWAVLAVTYCIK
QYEMNGGVSDSMLVNTVLMLVYITKFFWWEAGYWSTMDIAHDRAGFYICWGCLVWVPSIYTSPGMYLV
NHPVHLGTQLAVLILISGLLCIYVNYDCDRQRQVFRRTNGKCLIWGKAPSKIVASYKTEQGETKTSLLLISG
WWGVSRHFHYVPEILAAFFWSVPALFNHFLPYFYVIFLIILLLDRAKRDDDRCSSKYGKYWKMYCDKVRY
RVVPGIY*

A. officinalis DWF5	100.00	75.12	77.35	79.63	73.78	83.54	79.17
***S. polyrhiza* DWF5**	**75.12**	**100.00**	**78.90**	**79.49**	**78.21**	**84.38**	**79.49**
A. americanus DWF5	77.35	78.90	100.00	83.41	79.32	85.39	82.72
C. nucifera DWF5	79.63	79.49	83.41	100.00	81.11	85.64	83.87
S. cereale DWF5	73.78	78.21	79.32	81.11	100.00	86.65	82.49
D. alata DWF5	83.54	84.38	85.39	85.64	86.65	100.00	86.15
Z. officinalis DWF5	79.17	79.49	82.72	83.87	82.49	86.15	100.00

C24 reductase

>*A._thaliana*_DWF1
MSDLQTPLVRPKRKKTWVDYFVKFRWIIVIFIVLPFSATFYFLIYLGDMWSESKSFEKRQKEHDENVKKV
IKRLKGRDASKDGLVCTARKPWIAVGMRNVDYKRARHFEVDLGEFRNILEINKEKMTARVEPLVNMGQIS
RATVPMNLSLAVVAELDDLTVGGLINGYGIEGSSHIYGLFADTVEAYEIVLAGGELVRATRDNEYSDLYY
AIPWSQGTLGLLVAAEIRLIKVKEYMRLTYIPVKGDLQALAQGYIDSFAPKDGDKSKIPDFVEGMVYNPT
EGVMMVGTYASKEEAKKKGNKINNVGWWFKPWFYQHAQTALKKGQFVEYIPTREYYHRHTRCLYWEG
KLILPFGDQFWFRYLLGWLMPPKVSLLKATQGEAIRNYYHDMHVIQDMLVPLYKVGDALEWVHREMEV
YPIWLCPHKLFKQPIKGQIYPEPGFEYENRQGDTEDAQMYTDVGVYYAPGCVLRGEEFDGSEAVRRMEKW
LIENHGFQPQYAVSELDEKSFWRMFNGELYEECRKKYRAIGTFMSVYYKSKKGRKTEKEVREAEQAHLET
AYAEAD*

>*S._polyrhiza*_DWF1
MPDLNTPLRPKRKKVWVDYFVQFRWIIVIFVVLPISATMYFLTYLGDLRSESKSFKRRQKEHDENVK
KIVNRLKQRDPKRDGLVCTARKPYIAVGMRNVDYKRARHFEVDLSEFRNILDIDEERMIAKVEPLVN
MGQITRVTVPMNLSLAVVAELDDLTVGGLINGYGIEGSSHIYGLFSDTVVALEVVLADGRVVRATKD
NEYSDLFYGIPWSQGTLGFLVSAEIKLIPVKEYMRLTYTPARGSLQDLAQAYADSFAPRDKDPGKVPD
FVEGMIYNPNEGVMMTGRYASKEEAKRKGNVINSVGWWFKPWFYQHAQTALKKGEFVEYIPTREY
YHRHTRCLYWEGKLILPFGDQWWFRWTLGWLMPPKVALLKATQGEAIRNYYHDMHVIQDMLVPL
HKVGDALEWVHEEMEVYPIWLCPHRLFKLPVKTMIYPEPGFELHDRQGDTKFAQMFTDIGVYYAP
GAVLRGEEFDGAEAVRRLEEWLIQNHGFQPQYAVSELTEKNFWRMFDAAHYEHCRRKYNAIGTFM
SVYYKSKKGRKTEKEVQEAEQAIAEAAYAEEA*

>*S._cereale*_DWF1
MADLQTPLVRPKRKKVLVDYLVQFRWILVIFVVLPASALIYFNIYLGDMWSAMKSEKKRQKEHDENVQKVVKR
LKQRNPKKDGLVCTARKPWIAVGMRNVDYKRVRHFEVDLSAFRNILEIDAERMVAKVEPLVNMGQISRATCPM
NLSLAVVAELDDLTVGGLINGYGIEGSSHIYGLFSDTVVALEIVLADGRVVRATKDNEYSDLFYGVPWSQGTLGF
LVSAEIKLIPIKEYMRLTYTPVKGPLKEVAQAYADAVAPRDGDPAKVPDFVEGMVYSATEGVMMTGVYASKEE
AKKKGNKINSVGWWFKPWFYQHAQTALKKGEFVEYIPTREYYHRHTRCLYWEGKLILPFGDQFWFRFLFGWL
MPPKVSLLKATQGDAIRNYYHDNHVIQDMLVPLYKVGDALEFVHHEMEVYPLWLCPHRLFKLPVKTMIYPEPG
FEHHQRQGDTSYAQMFTDVGVYYTPACIFRGEEFDGAESVKRLEQWLIENHSYQPQYAVTELNEKDFWRMFDA
SHYEHCRQKYGAVGTFMSVYYKSKKGRKSEKEVQEAEAAILEPAYADEA*

>*A._americanus*_DWF1
MLMFLLFCFFFPVRSSIMAGDLEAPLRPKRKKVWVEYLVQFRWIIVIFVVLPISFTIYFLTYLGDIRSESKSFK
RRQKEHDENVKKVVNRLKQRNPSKDGLVCTARKPYIAVGMRNVDYKRARHFEVDLSAFRNILEIDKERMI
ARCEPLVNMGQISRVTVPMNLSLAVVAELDDLTVGGLINGYGIEGSSHLYGLFYDTVVALEIVLADGRLVR
ATRDNEYSDLFYAVPWSQGTLGLLVAAEITLIPVKEYMKVTYEPVVGSLSDIAQAYADSFAPRDGDPRKIP
DFVETMIYTPTEAVCMTGVYASKEEAKKKGNVINCVGWWFKPWFYQHAQKALKKGKFVEYIPTREYYHR
HTRCLYWEGKLILPFADQWWFRWTLGWLMPPKVSFLKATQGEAIRKYYHDMHVIQDMLVPLYKVGPAL
EFVHREMEVYPVWLCPHLLFKAPMKTMINPEAGYEQEGRQGDTKDGQMFTDVGVYYAPGPVLRGEEFDG
SEAVRRMEEWLIENHGFQPQYAVSELTEKNFWRMFDADLYEYCRKKYGAVGTFMSVYYKSKKGRKTEK
EVREAEQAHLETAYAEAD*

>*D._alata*_DWF1
MSDLHTPLRPKRKKVLVDYLVQFRWIVVIFVVLPISFTVYFLLYLGDMRSEMKSFKRRQKEHDENVKKVV
NRLKQRDPKKDGLVCTARKPYIAVGMRNVDYKRARHFEVDLAAFRNILEIDKERMIAKVEPLVNMGQISR
ATVPMNLSLAVVAELDDLTVGGLINGYGIEGSSHIYGLFADTVVALELVLADGRVVRATKDNEFSDLFYA
VPWSQGTIGLLVSAEIKLIPVKEYMKLTYKPVRGNLKELAQAYADSFAPRDGDPAKIPDFVEAMIYTPTEG
VMMTGKYASKEEAKQKGNVINEVGWWFKTWFYQHCQTALTRGEFVEYIPTRQYYHRHTRSMYWEGKLI
LPFGDQWWFRWTLGWLMPPKVSLLKATQGEGIRNYYHDMHVIQDLLVPLYKVGDSLEFVHREMEVYPIW
LCPHRLFKLPVKTMIYPEPGFELHRRQGDTNNAQMFTDIGVYYAPGPVLRGEEFNGSEAVQRLEEFLIQNH
GFQPQYAVSELTEKNFWRMFDGELYEKCRHKYGAVGTFMSVYYKSKKGKKTEKEVQEAEQAIVEQAYA
EDN*

>*A._officinalis*_DWF1
MDKVWNAEHRLLGEVTSLRVEKGVGSKQAAEASAPGAPEVDKLKQELEENQKKAKRMEEVLQERDRAE
VRANMEKDVAVHARIQVTTMSDLQTPLRPKRKKDLVDYLVNFRWILVIFVVLPVSFTIYFLIYLGDVKSAM
KSEKRRQKEHDENVKKVVNRLKQRNPKKDGLVCTARKPYIAVGMRNVDYKRARHFEVDLSAFRHILEID
KERMVAKVEPLVNMGQITRATVPMNLALAVVAELDDLTVGGLINGYGIEGSSHIYGLFSDTVVALELVLA
DGRVVRATKDNEYSDLFYAVPWSQGTIGFLVSAEIKLIPVKEYMKLTYTPARGNLRDLAQAYADSFAPRD
GDSAKVPDFVEGMIYNPNEGVMMTGRYASKEEAKQKGNVINSVGWWFKPWFYQHAHKALTKGEFVEYI
PTRDYYHRHTRCLYWEGKLILPFGDQWWFRWCFGWLMPPKVSLLKATQGEAIRNYYHDRHVIQDLLVPL
YKVGEALEFVHREMEVYPIWLCPHRIFKLPIRTMIYPEPGFEHHQRQGDTSYAQMYTDIGVYYAPGPVLRG
EEFDGVEAVRRLEDWLIKNHSFQPQYAVSELNEKNFWRMFDGGLYEQCRKKYGAVGTFMSVYYKSKKG
RKTEKEVQEAEQAIAEPAYAEES*

>*C._nucifera*_DWF1
MSDLQTPLRPKRKKDWVDYLVHFRWMVVIFVVLPISFTIYFLIYLGDVKSAMKSEKRRQKEHEENVKKVV
NRLKQRDPKKDGLVCTARKPYIAVGMRNVDYKRARHFEVDLSAFRNILEIDKERMIAKVEPLVNMGQITR
ATVPMNLALAVVAELDDLTVGGLINGYGIEGSSHIYGLFSDTVVAFEVVLGDGRVVRATKDNEYSDLFYG
IPWSQGTLGLLVSAEIKLIPIKEYMRLTYTPVRGNLKELSQAYADSFAPRDGDPSKVPDFVEGMIYTPTE
GVMMTGRYASKEEAKKKGNVINSVGWWFKPWFYQHAQKALKKGEFVEYIPTRDYYHRHTRCLYWEGK
LILPFGDQWWFRWFFGWSMPPKVSLLKATQGEAIRNYYHDKHVIQDLLVPLYKVGDALEYVHQEMEVYP
IWLCPHRLFKLPVKTMVYPEPGFELHYRQGDTSYAQMFTDIGVYYAPAAVLRVEEFNGAEAVHQLEEWLI
HNHGFQPQYAVSELTEKNFWRMFDGAHYEHCRRKYRAIGTFMSVYYKSKKGKKTEKEVQEAEAAILEPA
YAEEA*

>*Z._officinalis*_DWF1
MSDLQQPLVRPKRKKVLVDYLVQLRWIVVIFVVLPISFLIYFGLYLGDVKSAMKSEKRRQKEHDENVKKV
VNRLKQRDPKKDGLVCTARKPYIAVGMRNVDYKRARHFEVDLSAFRNILEIDKERMVAKVEPLVNMGQIT
RYTVPMNLALAVVAELDDLTVGGLINGYGIEGSSHIYGLFSDTIVAMEVVLADGKVVRCTKDNEYSDLFY
GVPWSQGTLGFLVSAEIKLIPIKEYMRLTYAPFRGNLQEIAQAYADSFAPRDGDPAKVPDFVEGMIYTPT
ESVLMTGKYASTEEAKRKGNVINSVGWWFKPWFYQHAQTALKRGEFVEYIPTREYYHRHTRCLYWEGKL
ILPFADQWWFRWLMGWMMPPKVSLLKATQGEAIRNYYHDNHVIQDLLVPLYKVGDALEFVHREMEVYPI
WLCPHRLFKLPVRTMVYPEAGFDHHHRQGDTSYAQMFTDIGVYYAPGPVLRGEVFNGAEAVRNLEEWLI
QNHSFQPQYAVSELTEKNFWRMFDGSHYEHCRRKYGAVGTFMSVYYKSKKGKKTEKEVQEAESEILEPA
YADEA*

A. americanus DWF1	100.00	80.18	84.82	84.82	80.94	81.96	81.61
S. cereale DWF1	80.18	100.00	82.32	83.93	85.36	85.36	85.56
D. alata DWF1	84.82	82.32	100.00	86.79	87.68	87.14	86.25
***S. polyrhiza* DWF1**	**84.82**	**83.93**	**86.79**	**100.00**	**86.96**	**88.57**	**85.89**
A. officinalis DWF1	80.94	85.36	87.68	86.96	100.00	88.75	87.50
C. nucifera DWF1	81.96	85.36	87.14	88.57	88.75	100.00	89.29
Z. officinalis DWF1	81.61	85.56	86.25	85.89	87.50	89.29	100.00

Phospholipid:sterol acyl transferases

>*A._thaliana*_PSAT
MGANSKSVTASFTVIAVFFLICGGRTAVEDETEFHGDYSKLSGIIIPGFASTQLRAWSILDCPYTPLDFNPLDL
VWLDTTKLLSAVNCWFKCMVLDPYNQTDHPECKSRPDSGLSAITELDPGYITGPLSTVWKEWLKWCVEFG
IEANAIVAVPYDWRLSPTKLEERDLYFHKLKLTFETALKLRGGPSIVFAHSMGNNVFRYFLEWLRLEIAPKH
YLKWLDQHIHAYFAVGAPLLGSVEAIKSTLSGVTFGLPVSEGTARLLSNSFASSLWLMPFSKNCKGDNTFW
THFSGGAAKKDKRVYHCDEEEYQSKYSGWPTNIINIEIPSTSVTETALVNMTSMECGLPTLLSFTARELADG
TLFKAIEDYDPDSKRMLHQLKKLYHDDPVFNPLTPWERPPIKNVFCIYGAHLKTEVGYYFAPSGKPYPDNW
IITDIIYETEGSLVSRSGTVVDGNAGPITGDETVPYHSLSWCKNWLGPKVNITMAPQPEHDGSDVHVELNVD
HEHGSDIIANMTKAPRVKYITFYEDSESIPGKRTAVWELDKTNHRNIVRSPVLMRELWLQMWHDIQPGAKS
KFVTKAKRGPLRDADCYWDYGKACCAWQEYCEYRYSFGDVHLGQSCRLRNTSANMLLQYI*

>*S._polyrhiza*_PSAT
MRATARRGRRIIIVSVLLLLFCSTRGISATDDLIGGKLSGIIIPGFASTQLRAWSVLDCPYSPLDFNPLDL
VWLDTTKLLSAVNCWLKCMVLDPYNQTDHPECRSRPDGGLSAITELDPGYITGPLSSVWKEWVKW
CIEFGIDANAIIAVPYDWRLSASMLEERDLYFHKLKLTFETVLKLRGGPSLVFAHSMGNNVFRYFLE
WLKLEIAPKHYIQWLDDHIHAYFAVGAPLLGSSESVKGILSGVTFGLPVSEGTARLMYNSFGSSLWLL
PFSKYCRDDNSYWKHFFDGKDGHHHTPYCDGKEFQSNYSGWPVDIVNIEVPFVRGTEAFPSISEVAQ
EVTSSMECSLPVQMAFSAREIADGSFFKAIEVYDLDSKRLLHQLHKYYQGDPVLNPLTPWDRPPLKNI
FCIYGIGSKTEVGYYFAPSGKPYPDNWIITDVIYEQEGTLFSRSGNAVSGNQGVTSGDDTVPYNSLSWC
KTWLGPKVNITRAPQSEHDGSDVQTILNADHRKGEDLVPNMNRAPQVKYITYYEDAESIKGKRTAV
WELDKGKHRNIVRSPVLMRELWLQMWHDVHPGARAKFVTKARRGPLRDVDCYWDYGKARCAWP
EHCEYRYVFGDVHLGQSCRLKNASHELLLHY*

>*S._cereale*_PSAT
MPPWRFRSGGLACAMAIAVATAAAASVGVAAGVADGEAEFDYRKLSGIIIPGFASTQLRAWSVLDCPYSPF
DFNPLDSVWLDSTKLFSAVNCWLKCMLLDPYNQTDHPECKSRPDSGLSAITELDPGYITGPLSSIWKEWVK
WCVEFGIEANAIIAVPYDWRLPPSLLEERDLYFHKLKLTFEIALKLRGGPSLVFAHSMGNNVFRYFLEWLKL
EIAPKHYIQWLDKHIHAYFAVGAPLLGSTESVRATLSGTTSGLPVTEGTARLMFNSFAASLWLLPFSKYCKA
DNVYWKHFFEGKAHMNRQQCDAMEYSSDNSGWPTTLVSIEVPTTRGTDAYPSIMDITENITSNMECGNPT
LLSFSAREVSDGTLFKTMLDYDPQSKALIHQLEKYYQGDPVLNPLTPWERPPIKNVFCIYGIDTKTEVGYYF
APSGKPYPDNWIITDVIYEFEQSLLSRSGHSFSGKPNNSSGDGTVSYNSLSWCKQWLGPKVNITRAPQAEHD
GSDLQTSMNAEHYHGEDLFPNMTRAPHVKYITYYEDAESIPGWRTAVWELDKANHRNIVRMPVVMREL
WLEMWHDMHPHSKSKFVTKAFRGPLRNEDCHWDYAKARCGFPEFCEYRYTFGDVHLGMSCRLKNSSTM
LLRQYL*

>*A._americanus*_PSAT
MNFARTNKTHVGTRSRKRVNTDTSPMLAPKSTSQASPNPKTPTSSASANHRVSPSSTMDPRRPSLLLLLVLL
RSSSSSVEAAEGGGGDLSKLSGVIIPGFASTQLRAWSVLDCPYSPLDFNPLDSVWLDTAKLLSAVNCWLKC
MVLDPYNQTDHPECKSRPDCGLSAITELDPGYITGPLSSVWKEWLKWCIEFGIEANAILAAPYDWRLSPSM
LEERDLYFHKLKFTFESALKLRGGPSLVFAHSLGNNVFRYFLEWLKLEVAPKTYIQWLDEHIHAYFAVGPP
LLGSTESVKATLSGATFGLPVAEGTARLMYNSFGSSLWMMPFSKYCRADSTYWKNFFDGKNREQYTHHC
EDMEFQSNYSGWPTDIINIEVPSIRGTDAYPSISEVAQTVPSDMECGLPTQLSFSAREVSDGTFFKAIEEYDPD
SKRLIYQLHKLYQGDPVLNPLTPWDRPPLKNVFCIYGIDKKTEVGYYFAPSGKPYPDNWIITDIIYEHEGTLT
SRSGNSVDGNPGASSGDETVPYHSLSWCKTWLGTKVNITRAPQAEHDGSDVQINLNVEHHPDEDIIPNMTR
TPRGRYITYYEDSESIPGRRTAVWEVDKANHRNIVRSPVLMRELWLQMWHDIHSDTKEKFVTKGKRGPLR
DVDCYWDYGKARCAWPEYCEYRYVFGDVHLGQSCRLRSSSADLLLNY*

>*D._alata*_PSAT
MAERRGGDEWHRSLRLLVLLVALSLMFREADGSSSEPFYKKLSGIIIPGFASTQLRAWSVLDCPFSPLDFNP
LDSVWLDTTKLLSAVNCWLKCMVLDPYNQTDHPECKSRPDSGLSAITELDPGYITGPLSSVWKEWLRWCI
EFGIDANAIIAVPYDWRLSASMLEERDLYFHKLKVTFETALKLRGGPSLVLAHSLGNNVFRHFLEWLKLEIA
PKHYVQWLDEHIHAYFAVGAPLLGATESVKATLSGVTFGLPIAEGTARLMYNSFGSSLWLMPFSKHCKSD
NIYWKQFFEGRGSRHHKPHCDDTEFRSNYSGWPTDIINIEVPSMRGMDAYPSVAEVAEDLASNMECGRPV
QMSFSAREVSDGTFFKAIEDFDPDSKRLLHQLQKYYQGDPVLNPHTPWERPPLKNIFCIYGIDSKTEVGYYF
APSGKPYPDNWIITDIIYELEGTLLSRSGNTVNGNPSAVSGDETVPYHSLSWCKTWLGSKVNITRAPQAEHD
GSDVHVRLNVDHHPGEDILANMTRAPRVKYITYFEDAESIPGRRTAVWELDKG*

>*A._officinalis*_PSAT
MGQRYAAPRSILLLLLLLLSFAGATSDLYYSKLSGIIIPGFASTQLRAWSVLDCPFSPLDFNPLDSVWLDTTK
VLSAVNCWLKCMLLDPYNQTDHPECKSRPDSGLSAITELDPGYITGPLSSVWKEWLKWCIEFGIEANAIVA
VPYDWRLSPSMLEERDLYFHKLKLTFETTLKLRGGPSLVFAHSLGNNVFRYFLEWLKLEIAPKHYIQWLDE
HIHAYFAVGAPLLGSTESVKATLFGFTFGLPVAEVGYYFAPSGKPYPDNWIITDVVYEYEGTLFTRSGNSIS
GNPGAASGDTTVAYNSLSWCKTWLGSKVNITRAPQAEHDGSDVQVHLDVEHIQGEDIVPNMTRAPRVKY
MTYYEDSESIPGRRTAVWELDKANHRNIVRSPVLMRELWLQMWHDIHPDASSKFVTKGK*

>*C._nucifera*_PSAT
MAVGGGAQRLRTRRLSLLLVPVLLVGGVVLGEDPSSSSGGGERDLYASKLSGIIIPGFASTQLRAWSVLDCP
YSPLDFNPLDSVWLDTTRVLSAVNCWLKCMMLDPYNQTDHPECKSRPDSGLSAITELDPGYITGPLSSVWK
EWLKWCIEFGIEPNAIIAVPYDWRLSASKLEERDLYFHKLKLTFETALKLRGGPSLVFAHSMGNNVFRYFLE
WLKLEIAPKQYIQWLDEHIHAYFAVGAPLLGSTESVKATLFGFTFGLPVAEGTARLLYNSFGSSLWLMPFS
KHCKADNLYWKHFFAEGEGRRHAYHCDEIEFQTNYSGWPTEIINIELPSIRGSDAYPSISDVAPDMTSSMEC
GRPIQLSFSAREVSDGTFFKAIEDYDPDSKRLLYQLRKYYQDDPVLNPLTPWERPPLKNIFCIYGIDLRTEVG
YYFAPSGKPYPDNWIITDVIYEHEGALFSRSGNSVSGNPGAVTGDGTVPYNSLSWCKTWLGSKVNITRAPQ
AEHDGSDVQISLNVEHHQGEDILPNMTRTPRVKYITYYEDSESIPGRRTAVWELDKANHRNLVRSQVLMRE
LWLEMWHDIHPDATSKFVTKARRGPLRNADCYWDYGKARCAWPEYCEYRYAFGDVHLGQSCRLKNSST
ELLLSYL*

>*Z._officinalis*_PSAT
MSVLLRPLILRRQEEERRRRRSPSARIIVVFLFLIFVGGPSAVAGEEAHDSNGADGGGETLSYSKLSGIIIPGFA
SAQLRAWSVLDCPYSPLDFNPLDLVWLDSTKLLSAVNCWLKCMLLDPYNQTDHRECKSRPDSGLSAITEL
CPGYITGPLSSVWKEWLTWCIEFGIEANAIIAVPYDWRLSASMLEERDLYFHKLKLTFETTLKLRGGPSIVFA
HSMGNNVFRYFLEWLKFEIAPRQYIQWLDEHVHAYFAVGAPHLGSTEALKASLIGVTFGLPIAEGTARLMQ
NSFGSSLWLIPFSKYCKADNIYWKHFYEGKKGHYHTHHCDEVEFKTMYSGWPTDIVNIELPSAPEFEAYPSI
SKVAQETSMECGRPDQLSFNAREVSDGTLFKAIEDYDPDSKRLLYQLHNFYQGDPVLNPLTPWERPPLKNI
FCIYGIDSKTEVGYYFAPSGKPYPDNWIMTDVVYEYEGTLLSRSGNSVSGNPGPVSGDGTVSYNSLAWCKT
WLGSKVNITRAPQSEHDGSDVQVELNVDHHQGNDLIPNMTRAPRSKYVTYYEDSESIPGRRMAVWELDK
ANHRNIVRSPVLMRELWLQMWHDIHPDAKSKFVTKAKRGPLRDVDCYWDYAKARCAWSEYCEYRYVFG
DVHLGQSCRLKSSSEDLLALYL*

S. cereale PSAT	100.00	74.08	73.62	72.66	73.46	79.23	75.27
D. alata PSAT	74.08	100.00	78.64	77.61	79.42	83.11	81.27
***S. polyrhiza* PSAT**	**73.62**	**78.64**	**100.00**	**77.73**	**78.32**	**82.28**	**79.02**
Z. officinalis PSAT	72.66	77.61	77.73	100.00	76.34	83.86	80.25
A. americanus PSAT	73.46	79.42	78.32	76.34	100.00	84.26	80.81
A. officinalis PSAT	79.23	83.11	82.28	83.86	84.26	100.00	87.71
C. nucifera PSAT	75.27	81.27	79.02	80.25	80.81	87.71	100.00

acyl CoA:sterol acyltransferase

>*A._thaliana*_ASAT
MASFIKAWGLVIISLCYTFFIAKLVPKGIKRLILFFPVFLIFFIVPFLIYSLHLLGITAFFIAWLANFKLLLFALGR
GPLSSNHKPLSLPIFLAVSCLPIKIQLSPKPTKTHSHEGSTEGPLIYTIKAVFVVLIIKAYEYSTKLPEKVVLTLY
AIHIYFALEIILAATAAAVRAMSDLELEPQFNKPYLATSLQDFWGRRWNLMVTGILRPTVYEPSLQLFSVLG
PNYSQILAAFGTFVVSGIMHELIFFYMGRLRPDWKMMWFFLINGFCTTVEIAIKKTINGRWRFPKAISQVLT
LTFVMVTALWLFLPEFNRCNIVEKALDEYAAIGAFAVEVRRKLTAYLF*

>*S._polyrhiza*_ASAT
MEGVRGFIRVWLVAVPVALAYCYSLPSLLRPGKWRLLAILPVVVLFALLPFSFSTVHLRAISALFLCW
LGVFKLLLFAFDRGPLCDAGATKRFSLPSFFAIASLPIMIRRRPPSSTCWGRADPVFTLLGFAAKGLVL
AFVVSLYPHRPRFYRAFVLALYSLHSYLSLDLVLACAAAVGLALLPAGTALEPQSAPPFLCSSLQDFW
GGRWNLMVSSILRSAVYAPIRHIWGPAAGVMAAFLVSGLMHEVMFFYLTLQPPTGEVGSFFVLHGF
CTVAEVAAKGWWTRRGMTPLHPALAAMLTIGFVLATAWWLFLPQILRSPAEELILKEAEAVGLLFM
EAARRVLW*

>*S._cereale*_ASAT
MELLLQDSIPRVYLAAAAAALYARALSSLLWPGLPRLAALLPVLAFLAAAPLAFTSSTNVRGTAAFFLAWL
GTFKVALLAAGRGPLDPALPVLTFLFTALLPVKLRGAPAPAKASKPVSLVSCAVKVAVIAVLLWLYQFSDR
MHLYMRLALYGVHMYCFFDLLLPCIAAAGGALGMEMEPQFDRPYLATSLRDFWGRRWNLMVSAILRPSV
YDPVRARAGSATGVLASFAVSGLMHEAMVCYLSLRWPPSGGMAAFFVLHGACCVAEGWWARRWATRG
WPPLPRPVATALVGLFVTATSFWIFFPALLPGVEEKLMEEWAAVGAFFLDAGGKVPSYGQRSES*

>*A._americanus*_ASAT
MESEIQNLMKVTIWVLLSLTYTHHFTPKTPPGLPRLLLLLPVLLLFILLPLSFSSLHLRGISAFFLSWLANFKL
LLLSFNSGPLSTNLSLLSFISSTALPIKIHKSQTPPPPKPPLFLSIKTLLLILIITLYRYKDSIPNWVLLSLYSLHVY
LALEIVLASAAWAARVLIRAEIEPQTDEPYLASSLREFWGRRWNLTVTRVLRPAVYDPLRRLGWGAAAAG
GVAFLVSGLMHEVMFWYLGGGRPTWVVTWFFVAHGVCVAVEGEVGRVLEGWGVRVHRVVKVVLTVG
FVLVSAGWWFWPPLLGTGVDGVVIEEYGRFGKWVMGGWKDFVGFSWKGFFVLGNE*

>*A._officinalis*_ASAT
MTYARFASSSIRPGKLRLLSLLPVILLLPCLPWRFTSVNLRGTTAFFLAWLGVFKLLLLSFGVGPLSPHLPLPT
FIAISSLPVKIQTSCHPKSDTDPSLIPFCIKLALLVLLTPIYRHKSQIHPWAVLALYSLYTYLILDLILSITKFSVG
TLLGLTLEPQANDPFKSDSLQDFWGRRWNLMVTGILRPSVYDPVRSRSGAGAGVVAAFIVSGANA*

>*D._alata*_ASAT
MADVPVLDGELQKLMKITLTIIACMSYARFISSKTSPGYLRLTLLLPILSLLPLLPFSFSSVHLRGISAFFLSWL
SIFKLLLLSFSLPPLHPSLPLPIFIATASLPVKLRSSSTSIATTSPLSLFSSIFKLLLLLLLFSLYPYKHLFPSYLLLA
LYCFHIYLALELVLSSARFLAGTLLGLDLEPQFNAPYFSTSLRDFWGRRWNLMVTSILRPSVYHPIRSRFGTA
AGVLAVFFVSGVMHELMFYYLTLSSPTGEVTCFFILHGFCMVVEGLFARRWKWGGVHPAVAPPLVLGFV
AVTGFWLFFPPILRTGTDEKTLEECAAMVAFLEKGARALLDRIGLLFNRV*

>*C._nucifera*_ASAT
MEGDLGSLAKVSLLVAASMTYVRFASSKIRPGKIRLLTLLPVLPLLLYLPWSFSSIHLRGICAFFLAWLGLFK
LLLLAFGLGPLSPSLPFLHFLSSASLPVKLLPSSKSKPSFSLSGHRLLSSAVKALLFAALIKLYRYRDDFHQYL
LLSLCCCHIYLALDLVLASAAAPAAILGLELEPQFDAPFRAVSLQDFWGHRWNLMVSAILRPSVYHPVHAR
CGTAAGILATFLVSGLMHEVMAYYITSAAPTGEMTAFFVLHGLCTVAEVWARKAGLWRPNPVLSVPITIGF
VFVTAFWLFFPPLLRRGEFELVLAECVAAMGFLDDAGRALVGWIGLIRNRTEPAVW*

>*Z._officinalis*_ASAT
MEELKAAGKVVLWVIAAMAGARFVASRTKPGRLRFLFLLPVVCLLPFLPMHFSTIHLRSISAFFLAWLALF
KLFLLSAGSGPLSADLPFFTFLASATLPVKLVHQKSPQKPKNPTTTSLFLPSAAKAALLSVLISCYRFKDTIHP
YLLLSIYSIHVYLALDLVLATAAVVASLLLPRGLQLEPQFDAPYLSTSLTDFWGRRWNIMVSAILRPSVYLP
VRNRCGRPAGVLATFFVSGLMHEFMFWYLTLAPPTGEVTAFFAIHAVCVVAEAVARRLGWRLPAPVATPL
TLGFVAATGFWLFFPPILRSRTDEAVLTECTAVMSFPEAAVTKILEWGRLT*

*A._thaliana*_ASAT	100.00	34.04	36.53	41.16	42.59	38.23	38.84	35.67
*S._cereale*_ASAT	34.04	100.00	43.37	36.83	48.11	45.90	49.70	47.42
***S._polyrhiza*_ASAT**	**36.53**	**43.37**	**100.00**	**41.21**	**47.22**	**46.22**	**47.13**	**44.14**
*A._americanus*_ASAT	41.16	36.83	41.21	100.00	47.87	47.01	46.87	44.55
*A._officinalis*_ASAT	42.59	48.11	47.22	47.87	100.00	57.87	53.74	53.24
*D._alata*_ASAT	38.23	45.90	46.22	47.01	57.87	100.00	56.68	54.65
*C._nucifera*_ASAT	38.84	49.70	47.13	46.87	53.74	56.68	100.00	55.69
*Z._officinalis*_ASAT	35.67	47.42	44.14	44.55	53.24	54.65	55.69	100.00

Sterol glycosyltransferase (UGT80A2)

>*A._thaliana*_UGT80A2
MPLEGSSSSDKAESSSTNQPRLDKSKTERQQKVTHILAEDAAKIFDDKISAGKKLKLLNRIATVKHDGTVEF
EVPADAIPQPIVVDRGESKNGVCADESIDGVDLQYIPPMQIVMLIVGTRGDVQPFVAIAKRLQDYGHRVRL
ATHANFKEFVLTAGLEFYPLGGDPKVLAGYMVKNKGFLPSGPSEIPIQRNQMKDIIYSLLPACKEPDPDSGIS
FKADAIIANPPAYGHTHVAEALKIPIHVFFTMPWTPTSEFPHPLSRVKQPAGYRLSYQIVDSLIWLGIRDMVN
DLRKKKLKLRPVTYLSGTQGSGSNIPHGYMWSPHLVPKPKDWGPQIDVVGFCYLDLASNYEPPAELVEWL
EAGDKPIYIGFGSLPVQEPEKMTEIIVEALQRTKQRGIINKGWGGLGNLKEPKDFVYLLDNVPHDWLFPRCK
AVVHHGGAGTTAAGLKASCPTTIVPFFGDQPFWGERVHARGVGPSPIPVDEFSLHKLEDAINFMLDDKVKS
SAETLAKAMKDEDGVAGAVKAFFKHLPSAKQNISDPIPEPSGFLSFRKCFGCS*

>*S._polyrhiza*_UGT80A2
MLNRIATVKGDGTVVFEVPESVKAGSLDLGSEDISGETADDETLEVTDIPPLQIVMLIVGTRGDVQPFI
AIGKRLQDYGHRVRLATHANFKEFVLTAGLEFYPLGGDPKVLAEYMVKNKGFLPSAPSEIPIQRKQM
KNIIFSLLAACKDPDESGIPFKPDAIIANPPAYGHTHVAEALKIPIHIFFTMPWTPTAQFPHPLSRVKQQ
AGYRLSYQVVDSMIWLGIRDMINDFRKKKLKLRPVTYLSGSQGSTSDIPTGYIWSPHLVPKPKDWGP
KIDVVGFCFLDLASNYEPPESLVKWLEAGEKPIYIGFGSLPVQEPQKMTNIIVEALEKTQQRGIINKG
WGGLGDLAEPKDFVYLLDNCPHDWLFSHCKAVVHHGGAGTTAAGLKAACPTTIVPFFGDQPFWGD
QVHARGVGPAPIPVDQFSLPKLIGAINYMLDPKVKENAVELAEAMASEDGVEGAVRAFFKHLPRRDP
DPKPSSPPSDFFKPCFGSIGRCFGCS*

>*S._cereale*_UGT80A2
MAAAADPTGGGEGVDQIKEAGDGAAAAAAATHNGDRPTHDAPGPSAPAPSTSSASDNGSLHRSSTMPGVI
KDTEITTETTGPSNLERSKTERRKQNNQADDPTKQLLDDKISIRKKLKMLNRIATVKDDGTVVVNVPSTLE
AAPIDVGSVDGYEDVVVEESLDGSDIPYKPPIQIVILIVGTRGDVQPFVAIGKRLQDYGHRVRLATHANYKE
FILTAGLEFFPLGGDPKVLAEYMVKNKGFLPSGPSEIPIQRKQMKEIIFSLYPACKDPDPDTGIPFKVDAIIANP
PAYGHTHVAEALKVPIHIFFTMPWTPTSEFPHPLSRVKTSAGYRLSYQIVDSMIWLGIRDMINEFRKKKLKL
RPVTYLSGAQGSGSDIPHGYIWSPHLVPKPKDWGPKIDVVGFCFLDLASDYVPPEELVKWLEAGDKPIYVG
FGSLPVQDPAKMTETIVKALEMTGQRGIINKGWGGLGTLAEPKDSIYVLDNCPHDWLFLQCKAVVHHGGA
GTTAAGLKAACPTTIVPFFGDQPFWGERVHARGVGPSPIPVDQFNLQKLVDAINFMLDPEVKEKAVELAKA
MESEDGVTGAVRAFLKHLPCKTDENSPPPTHGFLEFLGPVSKCLGCS*

>*A._americanus*_UGT80A2
MEEAKGESSNSSPIEHMDKILSRASSMPGEMKNPEKTVAPASPPNLERSRTERRRQSHLRTDPTAQIFDNKIS
DRKKLKMLNRIATVKDGTVVLDVPSSAEPEDFDVGSGDACNDTGDDEPVDLADIQYLPPMQIVILIVGTRG
DVQPFVAIGKRLQDYGHRVRLATHANFKEFVLTAGLEFFPLGGDPKILAGYMVKNKGFLPSSPSEIPIQRKQ
MKDIIFSLLAACKDPDDSGIQFKADAIIANPPAYGHTHVAEALKVPIHIFFTMPWTPTSEFPHPLSRVKQPAG
YRLSYQIVDSMIWLGIRDMINEFRKKKLKLRPVTYLSGAQGSANDIPTGYIWSPHLVPKPKDWGPKVDVVG
FCFLDLASNYEPPESLVKWLESGPKPIYIGFGSLPVQEPEEMTKIIVQALETTGQRGIINKGWGGLGNLDEPK
DSIYLLDNCPHDWLFLRCTAVVHHGGAGTTAAGLKAACPTTVVPFFGDQPFWGERVHARGLGPPPIPIDQF
SLQKLVSAINFMLDPKVKENAVELAKAMETEDGVTGAVKAFFKHLPPKMPEPETPQDPGLLGHCFGPVGK
CLGFS*

>*D._alata*_UGT80A2
MTNTMVLCLIRAINNHTERRSLGYLSCGTGNPIDDCWRCDSEWQKNRKRLADCGIGFGRNAIGGRDGRFY
MVTDSSDDDAVNPRPGTLRYAVIQEEPLWIVFKRDMVITLKEELIMNSFKTIDGRGVNVHIANGACITIQFV
TNIIIHGLHIHDCKPTGNAMVRSSPSHYGWRTMADGDAVSIFGSSHIWVDHCSLSNCADGLVDAIMGSTAIT
ISNNYFTHHNEVVVDPTPPIATCPYTLSNLLALHLSLCVSLSHAVEMATNGAAAGGSPPTVISGRNLPRANT
LPGGTKNGESSGTSTDEVNLERSRTERRRQNIPISDPTAQLFDDKIPVKEKIKMLNRIATVKDDGTVEVEVPS
NIDTTAFDHDTGNGDLVDEEPFDVADIQYVPPLQIVILIVGTRGDVQPFIAIGKKLQDYGHRVRLATHANFK
EFVLTAGLEFYPLGGDPKVLAEYMVKNKGFLPSAPSEITVQRKQLKDIIYSLYSACKDADVDTGVPFKADAI
IANPPAYGHTHVAEALKVPIHIFFTMPWTPTSEFPHPLSRVKQSAGYRLSYQIVDSMIWLGIRDMINDFRKR
KLKLRPVTYLSGSHTSGSDAPHAYIWSPHLVPKPKDWGPKIDVVGFCFLDLASNYVPPEPLVKWLEAGDKP
IYIGFGSLPVQDPQKMTKIIVEALQITGQRGIINKGWGGLGNLAEPKEFVYQLDNVPHDWLFLQCKAVVHH
GGAGTTAAGLKAACPTAIVPFFGDQPFWGERVHARGVGPAPIPVDQFSLQKLVDAINFMLDSKVKERAVE
LAKAMESEDGVTGAVKAFLKHLPKTQPETSPDQSPNAIDSILRPVKKCFGFA*

>*C._nucifera*_UGT80A2
MAESSSDMEASNGNVSSLSPSATSDRNLPRANTMPGGTNNTEKSETSSGQPNLGRSQTERRRQSNSREDPA
AQLFDDKISVKKKLKMLNRIATVKDDGTVVVEVPSNLEPASLDLRSEDVAAEPVDEEILDSMDFQYRPPMN
IVILIVGTRGDVQPFVAIGKRLQDYGHRVRLATHANFKEFVLTAGLEFYPLGGDPKVLAEYMVKNKGFLPS
APSEIPIQRKQIKEIIFSLLPACKDPDVDTGIPFKADAIIANPPAYGHTHVAEALKVPIHIFFTMPWTPTSEFPHP
LSRVKQPAGYRLSYQIVDSMIWLGIRDMINDFRKRKLKLRPVTYLSGAQGSASDIPHGYIWSPHLVPKPKD
WGPKIDVVGFCFLDLAKNYEPPESLVKWLESGERPIYIGFGSLPVQEPEKMTRIIVEALEITGQRGIINKGWG
GLGNLAEPKEFVYLLDNVPHDWLFLQCKAVVHHGGAGTTAAGLKAACPTTIVPFFGDQPFWGERVHVRG
LGPPPIPVDQFTLQKLVDAIKFMMDPEVKLRALELSKAMESEDGVTGAVKAFLKHLPQELPPQTPPTSGFID
PLLGPVRRCFGCS*

>*Z._officinalis*_UGT80A2
MAENGTSVEESKGNGKSVRFTASPVVDRGLPRSNTMPGGRTECAESSEAHLGKENLKRSRTEKHIQSSIEDP
TVKLFSDKVPNKEKIRMLNRIATVKDDGTVVVDVPSNLEIAPSELGSEDGNGEAADDEPFDSMDSQYRPPM
QIVILIVGTRGDVQPFVAIGKCLQDYGHRVRLATHANFKEFVLSAGLEFYPLGGDPKILAEYMVKNKGFLPS
APSEIPIQRKQMREIIFSLLPACKEPDVDTSIPFKADAIIANPPAYGHTHVAEALKVPIHIFFTMPWTPTSEFQH
PLSRVKQPAGYRLSYQIVDSMIWLGIRDMINEFRKKKLKLRPVTYLSGAQASTSDIPHGYIWSPHLVPKPKD
WGSKIDVVGFCFLDLASNYEPPKELVEWLEAGEKPIYIGFGSLPVQEPQKMTEIIVEALRITGQRGIINKGWG
GLGNLAEPKDYVYLLDNVPHDWLFLQCKAVVHHGGAGTTAAGLKAACPTTIVPFFGDQPFWGDRVHAR
GLGPQPIPVDQFSLQKLVDSIKFMMDPKVKDNAVELAKAMETEDGVSGAVRAFLKHLPIKKSTQAAAQPS
GIIGFLLGFLRCFSCS*

*A.*_officinalis_UGT80A2	100.00	52.19	50.68	49.74	51.21	51.83	56.51	52.78
*A._thaliana*_UGT80A2	52.19	100.00	72.99	74.13	73.22	75.36	78.54	75.23
*D._alata*_UGT80A2	50.68	72.99	100.00	72.80	75.13	75.52	79.03	79.21
*S._cereale*_UGT80A2	49.74	74.13	72.80	100.00	74.62	77.68	79.68	77.30
*Z._officinalis*_UGT80A2	51.21	73.22	75.13	74.62	100.00	75.74	80.89	80.85
*A._americanus*_UGT80A2	51.83	75.36	75.52	77.68	75.74	100.00	81.09	79.07
***S._polyrhiza*_UGT80A2**	**56.51**	**78.54**	**79.03**	**79.68**	**80.89**	**81.09**	**100.00**	**81.89**
*C._nucifera*_UGT80A2	52.78	75.23	79.21	77.30	80.85	79.07	81.89	100.00

Sterol glycosyltransferase (UGT80B1)

>*A._thaliana*_UGT80B1
MASNVFDHPLQELEGEDNGVKSEKASLLETSGSVDTTPEDSGHRSSDGHRGLDHCETAPVGLYGDMLIND
SEIQYSRSLTEKGSPAIHNLKLDRLSEQEKQKLIVELVRIQNDGTVEVIDNGTPVSELWEFEPTKGQSTITYEK
SLTESFRSIPRLKIAILVVGTRGDVQPFLAMAKRLQEFGHRVRLATHANFRSFVRAAGVEFYPLGGDPRELA
AYMARNKGLIPSGPSEISKQRKQLKAIIESLLPACIEPDLETATSFRAQAIIANPPAYGHVHVAEALGVPIHIFF
TMPWTPTNEFPHPLARVPQSAAYWLSYIVVDLMVWWSIRTYINDFRKRKLNLAPIAYFSTYHGSISHLPTG
YMWSPHVVPKPSDWGPLVDVVGYCFLNLGSKYQPREEFLHWIERGSPPVYIGFGSMPLDDPKQTMDIILET
LKDTEQRGIVDRGWGGLGNLATEVPENVFLVEDCPHDWLFPQCSAVVHHGGAGTTATGLKAGCPTTIVPF
FGDQFFWGDRIYEKGLGPAPIPIAQLSVENLSSSIRFMLQPEVKSQVMELAKVLENEDGVAAAVDAFHRHL
PPELPLPESSSEKKDEDDRPDLLQWFFIQIGKKCCLPCGGV*

>*S._cereale*_UGT80B1
MGCNSETSVAGEGSGSGDLRRRRGGRDGAGSSSSSFAEGTREFVLSSMDERFSGSVDADGFPSSRREGFGH
SKSTTATSSRFRGQDHAFVRSYSDRLLKCDLTLDMLSENEKMKIVEKLVKIQKDGTLEVDVTRSALVASEL
SEIDAFGSVHRDVEEVKSGFSKSVPKLKIAILVVGTRGDVQPFIALAKRLQEFGHHVRLASHVNFRTFVKSA
GVDFYPLGGDPRIMAQYMTKNKGFLMAAPTEISVQRKQVKEIIFSLLPACTEPDLDTGIPFRAQAIIANPPAL
GHLHIAEALGVPLHIFFTFPWTPTNEFPHPLARTPQSATYRLSYLIVDLIIWWGTRGFINDFRKKLNLPPIAYF
STYHGSISHLPTGYMWSPHLMPKPNDWGSLVDVVGYCFLNLGTKYQPPPELSQWLEQGSKPIYIGFGSMPL
DDEKKVTTIILDALRETGQRGIISRGWGDLGSFSEVPVDVFILEDCPHDWLFPRCTAVVHHGGAGTTAAGLI
AGCPTTVVPFFGDQFFWGEIVHARGVGPAPIRVTELTTEALSDAIRFMLDPEVKSRSMELAIAIGNEDGVAA
AVDSFHRHLPSELPLITPPPAPVEEERLDLLQLLSRYLEKCCLPFNS*

>*A._americanus*_UGT80B1
MAVSENEIVGFQEVKDSSIVVAPEAETAVEDRREGFSRHSSLMEISQFRESEDVGTSSQRVLEYSKTAPVGF
DAYVEHLIESHEVNYQRSKTEKKETPKHDLKLDRLSEREKRKMIENLVKIQNDGTVEVDVAHSASVASELL
ELDAVDLTPVDIESTAVELNKSIPRLKIAMLVVGTRGDVQPFLAFAKRLQEFGHHVRLATHSNFCTFVKSA
GIDFYPLGGDPRILAGYMARNKGFLPSGPAEISIQRKQLKAIIDSLLSACTEPDMDSGVPFKAQAIIANPPAYG
HAHVAEALGVPLHIFFTMPWTPTDEFPHPLAHVPQSAGYRLSYLIVDLIIWWGIRGFINDFRKKKLKLSPIAY
FSTYHGSISHLPTGYMWSPHLVPKPRDWGSLVDVVGYCFLNLGMKYQPKEELVHWIQRGTKPIYIGFGSM
PLEGAAKTTDIILEALRETGQRGIIDRGWGDLGNILEVPDNIFLLEDCPHDWLFPLCSAVVHHGGAGTTATG
LRAGCPSAIIPFFGDQFFWGERIHQVGVGPAPIPISELTVEVLSNSIKFMLDPEVKLRAMELAKKIENEDGVA
AAVNAFHRHLPTELPLPAPSSDESPNPFEWLFLFIEKWCCFPCTP*

>*A._officinalis*_UGT80B1
MTSSDFKGEGRLDEDEGFGGGCSRSSSLDDSRVLESEDSGFWESPLPGLEYCNSAPVRSNSSNLLYKDHDVI
FSRSKTLKKDFPKYDFKLDRLSESEKKKIIENLVKIQKDGTLEVDVSHDAPVPSELLELDSVDSVPCDSGENI
FKFSKIVPTLKIAILVVGTRGDVQPFIAIAKRLQVFGHRVRLATHVNFRNFVQSHGIEFYPLGGDPRIMAGY
MARNKGFLLSGPAEISIQLKQLKEIIHSLLPACTEPDLETGACFKAQAIIANPPAYGHAHVAEALGVPLHIFFT
MPWTPTNEFPHPMARVPQSAAYKLSYILFDLIVWLGIRGFINDFRKRKLNLPPVAYYSTYYGSISHLPTGYM
WSPHLVPKPKDWGGLVDVVGFCFLNLGTKYQPQKAFAEWIQQGPKPIYVGFGSMPIEDGKKIVNIILVSLK
ETGQRGIIGRGWGDLGISTDIPGDVFLIDDCPHDWLFPQCAAVVHHGGAGTTATGLRSGCPTTIIPFFGDQFF
WGDRVHKRGVGPAPIPIYELSVERLSNAIRCMLEPEVKLRAMELAREIENENGVKAAVDAFHKHLPPELPIP
PPLSDDPPCPLQWLFQAIERWCCLPFTS*

>*D._alata*_UGT80B1
MLDLMARESAGVEGLVKKEQVFSPEAVRAEEGSNVESLDLRISALEEPGLRDSPRPGFDHCHTAPIQHDSSS
LLHKDHEIALSRLKTDKRCNKKHDMLWDRLSESEKKKLIEDLVKIQHDGTVEVDVTRNVPVASELLELDA
LDASSGHTEDAISEACKPIPRLKIAMLVVGTRGDVQVFLAFAKRLQEFGHHVRLATHINFRTFVKSAGVEFY
PLGGDPRIMAGYMAKNKGFLLSGPTEISIQRKQLKEVIDSLLPACIEPDLDSGAPFKAEAIIANPPAYGHMHV
AEALGVPLHIFFTMPWTPTYEFPHPLARVPQSAAYRLSYLVVDLIVWWGIRAFINEFRKRKLKLSPIAYFST
YHGSISHLPTGYMWSPHLVPKPSDWGALVDVVGFCFLDLGINYQPQHEFADWIKLGPKPIYIGFGSMPLED
AEKTTAIILEALKETGQRGIIDRGWGHLGVISEFPADVFLLEDSPHDWLFPQCAAVVHHGGAGTTATGLRA
GCPTTVVPFFGDQFFWGDRIHERGVGPAPIPINDLNVERLSNAIKFMLDPEVKIRAMELAKLIEEEDGTANA
VNAFHRHLPSELPLPPSLEEPPNPLQWLLLTIEKWCCLPCAA

>*C._nucifera*_UGT80B1
MASNGVAVGLDDCEVVKGEGLAKKGMVVNLETAREVGVKEGTGGGSRLDEVGYYSANSLHSSVSGSRV
SASFNYLDFDVSGLKEPGSKSLPDSGLEHCSFAPVRSNSSSILVKGPELSFPRSKMGKKENPKYDLKLDRLSE
KEKKKIIENLVKIQNDGTVEVDVARSAPVASELLELDSVDATPTDVENTVFEFSKSVPKLKIAMLVVGTRG
DVQPFIAFAKRLQEFGHRVRLATHVNFRNFVKLAGVEFYPLGGDPRIMAGYMARNKGFLLAGPAEISIQRK
QLKEIINSLLPACTEPDLDSGAPFKAQAIIANPPAYGHAHVAEALGVPLHIFFTMPWTPTNEFPHPLARVPQS
AAYKLSYIIVDLIVWWGIRGFINDFRKRKLKLPPIAYFSTYHGSISHLPTGYMWSPSIVPKPSDWGILVDVVG
FCFLNLGTKYQPPKEFTQWIEQGPKPIYIGFGSMPLEDSKKITSIILEALKETGQRGIIDRGWGDLGSIFEVPV
DVFLVEDCPHDWLFPRCAAVVHHGGAGTTATGLRAGCPTTIVPFFGDQFFWGDRIYAKEVGPAPIPISELN
VERLANAIRFMLEPEVKLRAMELAKQIENEDGVVAAVNAFHRHLPPEMPVPPATTDEPPNPLEWLLQLIEK
WCCLPCTS

A._thaliana_UGT80B1	100.00	60.85	66.95	65.71	64.47	66.28
S._cereale_UGT80B1	60.85	100.00	63.83	65.38	66.78	67.64
A._americanus_UGT80B1	66.95	63.83	100.00	71.93	69.39	72.21
D._alata_UGT80B1	65.71	65.38	71.93	100.00	69.75	73.17
A._officinalis_UGT80B1	64.47	66.78	69.39	69.75	100.00	76.11
C._nucifera_UGT80B1	66.28	67.64	72.21	73.17	76.11	100.00
