# Supplementary material for: Duckweeds: Model organisms for research on plant sterols and steroids
Source: Plant Biol (Stuttg). 2025 Aug 25;28(1):18–30. doi: 10.1111/plb.70095 (PMC12710849; doi:10.1111/plb.70095)
Supplement: Supplementary file 3 — Data S3. Brassinosteroid biosynthesis. [file PLB-28-18-s001.pdf]

CYP90B1

>*A._thaliana*_DWF4
MFETEHHTLLPLLLLPSLLSLLLFLILLKRRNRKTRFNLPPGKSGWPFLGETIGYLKPYTATTLGDFMQQHVS
KYGKIYRSNLFGEPTIVSADAGLNRFILQNEGRLFECSYPRSIGGILGKWSMLVLVGDMHRDMRSISLNFLS
HARLRTILLKDVERHTLFVLDSWQQNSIFSAQDEAKKFTFNLMAKHIMSMDPGEEETEQLKKEYVTFMKG
VVSAPLNLPGTAYHKALQSRATILKFIERKMEERKLDIKEEDQEEEEVKTEDEAEMSKSDHVRKQRTDDDL
LGWVLKHSNLSTEQILDLILSLLFAGHETSSVAIALAIFFLQACPKAVEELREEHLEIARAKKELGESELNWD
DYKKMDFTQCVINETLRLGNVVRFLHRKALKDVRYKGYDIPSGWKVLPVISAVHLDNSRYDQPNLFNPW
RWQQVNKKFLSLTIEN*

>*S._polyrhiza*_DWF4
**MAGAGELPVLLVLRLLVLAVASVAGFVYLRRIRWAGSPRRPGNLPPGRRGCPFIGETLGYLKPHPAI
SMGEFMEDHISRFGKIYRSNLFGGPTIVSADAGLNRFILQNEGRLFECSYPRSIGEILGKWSMLVLVG
DMHKNMRTISLNFMCSARLQTQLLREVEQHALFILRSWREGSTFLAQDEAKKFTFNLMAKHIMSME
PGEPETESLKKEYITFMKGVVSAPLNFPGTAYWKAKKSRSTILKFIERKIEERRRRKADGRDGSEQDD
LLSSVLKQSNLSKEQILDLILSLLFAGHETSSVSIALAIYFLEACPEAVEELKAEHQRIILNKAHRGDDS
LLDWEDYKQMHFTQCVINETLRLGNVVRFVHRKALRDVRYKGYDIPAGWKVLPVFAAVHLDPSLY
GDPQQFNPWRWQRRNCGGAATAGANFMPFGGGPRLCAGSELAKLEMAVFLHHLVLSYRWELAEP
DQPLVFPFVDFPKGLPIRVHRTT***

>*S._cereale*_DWF4
MGCFPCFDSGSDGELLYPKQGGGGGGNGTGGRTAAAASSSGVGAREERPMVPPRVEKLPAGAEKARARGNAG
MKELSDLRDANGNVLSAQTFTFRQLTAATRNFREECFIGEGGFGRVYKGRLDGGQVVAIKQLNRDGNQGNKEF
LVEVLMLSLLHHQNLVNLVGYCADGEQRLLVYEYMPLGSLEDHLHDLPPDKEPLDWNTRMKIAAGAAKGLEY
LHDKAQPPVIYRDFKSSNILLGDDFHPKLSDFGLAKLGPVGDKSHVSTRVMGTYGYCAPEYAMTGQLTVKSDVY
SFGVVLLELITGRKAIDSTRPHGEQNLVSWARPLFNDRRKLPKMADPGLQGRYPMRGLYQALAVASMCIQSEAA
SRPLIADVVTALSYLASQIYDPNAIHASKKAGGDQRSRVSDSGRTLLKNDEAGSSGHKSDRDDSPREPPPGILNDR
ERMVAEAKMWGANLREKTRAAASAQGSLDSPTETG*

>*A._americanus*_DWF4
MDSLLHFFLFASAVLMALILCIKSTKYQNKNKKRFKLPPGRKGWPFVGETFPYLKPHHATTMGDFMENHIS
RFGKIYRSNLFGEPTIVSADAGLNRYILQNEGKLFECSYPKSIGGILGKWSMLVLVGDMHRQMRMISLNFM
SHAKLRSHLLRDVELHTLSVLDSWSVGRTFSAQEEAKKFTFNLMAKHIMSMGPGEVETEQLKREYITFMK
GVVSAPINLPWTAYWKALRSRSSILRVIERKMKERIKVMNCEGMNGDGDEDDLLYWVLKRSNLSLEQILD
LILSLLFAGHETSSVAIALSIYFLGGCPEAIRQLRDEHLEIASNKKQKGDVGLNWDDYKQMEFTHCVINETL
RLGNVVRFIHRKALRDIQFKGYDIPCGWKVLPVFTAVHLDDSIYKDPQQFNPWRWQKNSSSAAAMSGNFV
PFGGGPRLCAGSELAKLEMAVFVHHLVLKYDWELAVPDHPFAFPFVDFPKGLPIRVHVIT*

>*D._alata*_DWF4
MSSHMELLLLLSLALISLFLFFTLIKCRRRRVKLPPGTSGYPFIGETFAYLKPHKATSIGHFMKQHMSRYGKI
YRSNLFGEPTIVSADPGLNRFILQNEGRLFECSYPKSIGGILGKWSMLVLVGDMHRDMRMISLNFMSSLRLR
LFLLPEVERHTLLVLSSWKEGSSFSAQEEAKKFTFNLMAKNIMSMDPGEAETEKLRLEYITFMKGVVSAPL
NFPGTPYWKALKSRSSILSVIERKMDERIKFKASTDDDGEDKPEEDDLLSWALNNSNLSKEQILDLLLSLLF
AGHETSSMALTLAIFFLEGCPKAVEELRVEHLEINRKKKERGEIGLNWDDYKQMEFSQCVINETLRLGNVV
NFVHRKALKDVQYKGFDIPSGWKVLPVFAAVHLDPSLYSDPQEFNPWRWQVSLLIK*

>*A._officinalis*_DWF4
MEEDQKAPPPTGGPWLAVRRXXXTFAYLKPHLATSVGQFMNHHTTRYGEIYRSNLFGDPTIVSADPGLNR
YILQNEGRLFECSYPKSIGGILGKWSMLVLVGEMHREMRMISLNFMSSARLRGLLLPEVERHTLLVLGSWK
VGLRFSAQNEAKKFTFNLMAKNIMSMDPGEPETERLRLEYITFMKGVVSAPLNFPGTPYWKALKSRAIILSII
EQKMQERIQRISRKEERIEEDDLLGWALKQSNLSKEQILDLLLSLLFAGHETSSMALALAIYFLESSPKAVQK
LRKEHSEIAANKKLRGECGLNWDDYKKMEFSQCVINETLRLGNVVKFVHRKAIKDVRYKGYDIPCGWKV
LPVFAAVHLNSELYVNPHEFNPWRWQDPASIANMNNFMPYGGGPRLCAGSELAKLEMAVFLHHLVLNYS
WELAEPDQAFAYPYLDFPKGLPIKIWPIT*

>*C._nucifera*_DWF4
MSHSSLMSIIKELLFLLPPALLALFLYTNLIKIRKRKKPNLPPGISGWPFLGETFSYLKPHPATSIGQFMEQHIS
RYGKIYRSSLFGEPTIVSADADLNRFILQNEGKYFECSYPRSIGGILGKWSMLVLVGEMHREMRMISLNFMS
NVRLRSHLLPEVERHALLVLSSWRENSIFSAQEEAKKYTFNLMAKNIMSMDPGEPQTEKLRREYITFMKGV
VSAPLNFPGTPYWKALKSRSTILGVIELKMDERFQKMSQGQEICEEDDLLGWALKQSNLSKEQILDLLLSLL
FAGHETSSMALALTIFFLEDCPKAVQELQEEHLEIASRKKLKGESGLNWEDYKQMEFTQCVGSRNTTEETK
LGMD*

>*Z._officinalis*_DWF4
MLNFSVQFLLLPTLAVLLSVKLIKWKRKQLNLPPGRRGWPFVGETFGYLKPHPATSVGQFMEQHISRYGKI
YRSNLFGEPTIVSADAALNRFILQNEGKLFECSYPSSIGGILGKWSMLVLVGDMHREMRMISLNFMNNVRL
RSRLLPEVERHSLLVLRSWIHGSPFSALEEAKKFTFNLMAKNIMSMEPYEAKTEKLRLEYISFMKGVISAPL
KFPGTPYWKALKSRSNILNVIEQKMQERSQEMREQEGDEEVDDLLGWCLKHSNLSKEQTLDLLLSLLFAG
HETSSVALASAIFFLESCPKAVQELREEHSEIERKKLQRAESSLTWEDYKEMEFTQCVINETLRLSNVVRFV
HRKVLRDVEYKGYKIPRGWKILPVFASVHLDSSLYDDPHRFTPWRWQSNKQRRPTLCVQKNSVTSTTTNN
FMPYGGGPRLCAGSELAKLELAVFLHHLVLSYRWELAEPDHPLAFPFIEFPKGLPIKVYPI*

S. cereale DWF4	100.00	19.43	21.90	21.32	21.14	20.24	18.80
***S. polyrhiza* DWF4**	**19.43**	**100.00**	**69.38**	**70.49**	**64.01**	**68.51**	**68.55**
A. americanus DWF4	21.90	69.38	100.00	70.15	66.11	69.93	67.65
D. alata DWF4	21.32	70.49	70.15	100.00	75.42	77.95	72.48
C. nucifera DWF4	21.14	64.01	66.11	75.42	100.00	72.21	74.37
A. officinalis DWF4	20.24	68.51	69.93	77.95	72.21	100.00	73.11
Z. officinalis DWF4	18.80	68.55	67.65	72.48	74.37	73.11	100.00

CYP90A1

>*A._thaliana_*CPD
MAFTAFLLLLSSIAAGFLLLLRRTRYRRMGLPPGSLGLPLIGETFQLIGAYKTENPEPFIDERVARYGSVFMT
HLFGEPTIFSADPETNRFVLQNEGKLFECSYPASICNLLGKHSLLLMKGSLHKRMHSLTMSFANSSIIKDHLM
LDIDRLVRFNLDSWSSRVLLMEEAKKITFELTVKQLMSFDPGEWSESLRKEYLLVIEGFFSLPLPLFSTTYRK
AIQARRKVAEALTVVVMKRREEEEEGAERKKDMLAALLAADDGFSDEEIVDFLVALLVAGYETTSTIMTL
AVKFLTETPLALAQLKEEHEKIRAMKSDSYSLEWSDYKSMPFTQCVVNETLRVANIIGGVFRRAMTDVEIK
GYKIPKGWKVFSSFRAVHLDPNHFKDARTFNPWRWQSNSVTTGPSNVFTPFGGGPRLCPGYELARVALSV
FLHRLVTGFSWVPAEQDKLVFFPTTRTQKRYPIFVKRRDFAT*

>*S._polyrhiza_*CPD
**MAGERGLPPGSLGLPLLGETVRLISAYRSEHPEPFVDERLRRLRLGQRVFTTHLFGEPTVFSADPDVN
KQVLQGEGRLFQSSYPSSLTTLLGRRSLLVMRGAFHRRMHALLSATIASPTVIRDHLLPDIDHLIRRT
LDSWEEKADGGESIAGRRVLLQDQAKKITFELSVKQLMSMDPGEWTEGLRKEYLHLIDGFFSIPLPF
SFTTYGRALQAKRKREMVQRRAEDGKQVEEEAELKHGMSKKRRSSDLLGALLEAESEDGVEFTEEE
AVDFLLALLVAGYETTSTIMTLAVKYLADHPRALALLREEQEDIRRRKEEAGGHPDDALDWADYKS
MAFTQCVINETMRVGNIISGVFRRATADVPIKGGYTIPKGCKVFASFRAVHMDPAYYTDARSFDPWR
WWSAGGGNGRQQGGGASVFCPFGGGPRFCPGYELARVEISVFLHYLVTRFNWEPAEDDKAATS***

>*S._cereale_*CPD
MDAGALQLAAAAVVIVVAALVCRWFLVACAVRGRKQRPRLPPGSTGLPLIGETLRLISAYKTPDPEPFIDERVAR
HGGVFTTHIFGERTVFSADPAFNRLLLAAEGRAVSCSYPSSITTLLGARSLLLTRGTAHKRLHSLTLTRLGRPASQP
LLAHIERLVLATMRQWEPTATVRLLDEAKKITFNLTVKQLVSIEPGPWTESLRREYVKLIDGFFSIPFPFASFLPFTT
YGQALKSRKKVAGALREVIRKRMEERREENGVKEEMDGKREKKDMVEELLEAEGGSFSEEEMVDFCLSLLVAG
YETTSVLMTVAVKFLTETPAALAQLKEEHENMTKMKGENQPLEWADYKSMTFTQCVINETLRVANIIGGVFRRA
NTDIHFKGYTIPKGCKIFASFRAVHLNNEHYENARTFDPWRWQSNNKLQNEVGANLFTPFGGGPRLCPGYELAR
VVISVFLHHLVMRFSWEAAEEDRLVFFPTTRTLKGYPINLRRRSKSV*

>*A._americanus_*CPD
MESLLTWAGVVLSLSSLLLFLVIERIRQWQSERKAGVPPGRRGFPVVGETLRLISAYKTENPEPFVDERVAR
HGRVFTTHVFGERTVFSADPEANKYVLQNEGKLFESSYPSSISNLLGSHSLVLMKGSIHKHFHSLTVSFISPS
VIRDSLLPDIDRLIRHNLDSWAGRTVLLLSETKKITFELTVKQLMSVDPGEWTEKLRKEYLLLIEGFFSLPLPF
FFTTYGRALRARTKVVEILKTIVKERKERRSKKRSTSTSWEEVEEEKRDMLGALLEAEEGLTEEEGVDFMV
ALLVGGYETTSTIMTLAVKFLSDSPRALALLKEEHEGIRAKKKEGEALEWSDYKSMPFTQCVINETLRVANI
ISGVFRRAVADVRIKGYTIPKGWRVFASLRAVHLDSDHYKDARTFNPWRWQESVTTPTSNQGSGTFMPFG
GGPRLCPGYELARVELSVFLHYLVTCFSWTAPEDDRVVFFPTTRTLKQYPIDVRRRK*

>*D._alata_*CPD
MALSSSFAFLFLFFTHDQHSSSPTAPVGSILLLLILLSIIIIVLSSLIAVARKDKGQALPPGFLCLPLVGETLKLV
AAYKTEDPEPFIDERVSRHGRVFTTHVFGERTIFSADPEFNRQVLTGEGRSFECSYPSSISTLLGRHSLLLMK
GPLHKRMHSLILCRFSSPSALRESLLPDIDRLIRLTLDSWVPDGQEAITVRLLEQAKKITFDLTVKQLLSFDPG
VWTESLRREYLLVIEGFFSIPLALPCFLSSSTTYGRALRARNKVAEALREVLRKRREEKQMRDRDEYESEQQ
RQKKRDMVEELLEAEAGGLSEDEMVDFILALLVAGYETTSTIMTLAVKFLTENPPALALLLEEHDSIRAKK
LHESEPLDWTDYKSMPFTQCVINETLRVANIISGVFRRAVTDVHFKGYTIPKGCMVFSSFRAVHLDQNHYE
DARTFNPWRWQDKDVPLQTSAGACIFTPFGGGPRLCPGYELARVEISVFLHHLLTRFRWEAAEKDRLVFFP
TTRTLKGYPINVRPRRPVI*

>*A._officinalis*_CPD
MDDSLIIYVVASMVTVMAISCWRRRCQKSLAPVPSGYGKVFKSHLFGKPTIVSTDPEVSKFILQSVSEDFVP
FFPGSLTELVGKSSILVINGSLQRRVHGLIGSFFKSPILKAHITADMQRDLQEIMASWRDDQIVYIQEEAKYM
VFQIMVKTLIGLGPGEHLQFLRHQYQKFVAGIISIPVKIPGTRFYRSLQAKKRMMKLVKYIIQEKKKNNTGCI
SRDIIDAFLNDGSGQLTDDLILDNVIDLMIPAEDSVPVLITLAIKYLSDCPLVLQKLKEENTKLKHKSLEGED
LQWMDYMSLSFTQNVISETLRMGNIIPAVMRKAIKDVEVKDHFIPEGWCVLPYFRSVHLDKEIYDDAYKFN
PWRWKDKDTAASSFTPFGGGQRLCPGIDLSRLIASIFLHHLVTGFTWVAEEDHMVNFPTVRMQRNMPIKV
KRKNIDILLS*

>*C._nucifera*_CPD
MAPAAAAAAAAYSRYLLLLSTSDHQFLLLPVLMSVVAVLVLQLLRRSSWMRKMNNSVGGGKGLPPPGSV
GLPLVGETLRLIAAYKTEDPEPFIDERVRRHGRLFTTHVFGERTVFSADPEFNRVVLGGEGRSVECSYPSSIS
TLLGRHSLLLMRGALHKRMHSLTLTRLASPAAIRDSPLLFHIDRLVQTTLDSWAPQSRVLLLDQAKKITFEL
TVKQLVSYDPGDWTEALRCEYLLLIEGFFSIPFPSFLSFTTYGRAIKARRKVAEALREVIRKRKEEKMRNDD
DDDDDDDKEGQGRRKKDMVAELLEAEEGFSEEEMVDFLLALLVAGYETTPTIMTLAVKFVTDNPPALSLL
AEEHEKIRARKKDEMEPLDWSDYKSMPFTQCVINETLRVANIVGGIFRRAVTDLHFKGYTIPKGSKVFASFR
AVHLDPEYYEEARTFNPWRWQTNDAVQQASGAGIFTPFGGGARLCPGYELARVVISVFLHHLVTRFRCAL
HLI*

>*Z._officinalis*_CPD
MDSWSLQLLDVFLIVIASVVFWELGRWRRKRTTRGGLPPGSVGLPVVGETLRLIAAYKTEDPEPFIDERVRR
HGRLFTTHVFGERTVFSADPEFNRMVLAAEGRTVECSYPSSISTLLGAHSLLLMRGVRHKRMHSLTLTRLA
SPAAIRDAALLRHIDRLVRRTLDAWCQSPGPARVLLLDQAKKITFELTVKQLVSVDPGEWTESLRREYLLLI
EGFFSIPFPSFLSFTTYGRALKARKKVESALKEVIRKRKREREAESRGSDVAKRKADMLDELMDGAVEEEES
MTEEEMVDFLLALLVAGYETTPTIMTLAVKFLTDHPDALALLRAEQEEIRAHKDEEDPLTWTDYKSMPFT
QCVINETLRVANIISGVFRRAMADIHFQGYTIPKGCKVFISFRAVHLDPLYFEDARTFNPWRWHQNKESAAT
MQPYYTPFGGGSRLCPGYELARVVISVFLHYLVTRFSWEEAEKDRLVFFPTTRTLKSYPINVRYRNINSNGN
NNGKPVNNQSAPSSSSSMC*

A. officinalis CPD	100.00	37.76	38.89	34.35	37.65	34.16	34.11
S. polyrhiza **CPD**	**37.76**	**100.00**	**62.61**	**57.83**	**60.14**	**61.98**	**63.16**
A. americanus CPD	38.89	62.61	100.00	58.00	64.18	63.77	64.98
S. cereale CPD	34.35	57.83	58.00	100.00	64.48	65.08	67.01
D. alata CPD	37.65	60.14	64.18	64.48	100.00	68.75	67.83
C. nucifera CPD	34.16	61.98	63.77	65.08	68.75	100.00	73.76
Z. officinalis CPD	34.11	63.16	64.98	67.01	67.83	73.76	100.00

Steroid 5α-Reductase

>*A._thaliana*_DET2
MEEIADKTFFRYCLLTLIFAGPPTAVLLKFLQAPYGKHNRTGWGPTVSPPIAWFVMESPTLWLTLLLFPFGR
HALNPKSLLLFSPYLIHYFHRTIIYPLRLFRSSFPAGKNGFPITIAALAFTFNLLNGYIQARWVSHYKDDYEDG
NWFWWRFVIGMVVFITGMYINITSDRTLVRLKKENRGGYVIPRGGWFELVSCPNYFGEAIEWLGWAVMT
WSWAGIGFFLYTCSNLFPRARASHKWYIAKFKEEYPKTRKAVIPFVY*

>*S._polyrhiza*_DET2
MASDEALFYAAVGILYAISPLTFLPLQSFTAPFGRHARPGWGPFLPPAAAWFLMESPTIWLTLLLLPH
GRHRSHPLSLAIISLYLLHYLNRTVVYPLRLLRSGTKTGFPLCTAAAGFCFNLLNAYVQTRSASHYAD
YPPAGDVGWWVWARVAAGLAVFFWGMTVNVSSDLALLRLKKTAGGGYKVPEGGWFELVACPNY
MGEAAEWLGWAVAASTPAALGFFLYTCANLVPRASSHRRWYLQKFGPRYPPSRKCIVPFVF*

>*S._cereale*_DET2
MSDGGGGAAGGDALFSRCLLALYVISPVTVFLLRYVSAPYGKLSRPGWGPAIPAALAWCAMESPTLWLPL
LVFPPPLLAAAAASPLAALPPALYALHYVNRTVLHPLRIFRLRRAAAPVPVLVAACAFGFNLLNAYVQARS
WALHAARPASAFALARSLVGLALFAWGMRVNLAADKELLRLKEAGGGYKIPRGGWFDAVTCPNYFGEIV
EWLGYCLVAWSPAAWAFFLYTCANLMPRARDHRQWYLSKFGGEYPASRKAVIPCIY*

>*A._americanus*_DET2
MDDFYRFTLLTLYLLSPLTILPLQTITAPYGRHFRRGWGPSIPPSLAWLLMESPTVWFSALLLPLGRHRLHPK
SLLLCSAFLLHYTHRTLIYPLRLRRKCRPLRRPFPLSVAAFGFSFNLLNTYVQIRWVSHFVDYDRLPWWLIL
VGSAVFAAGMAVNVWSDLVLVGLKGEEGEYKVPKGGLFEWVCCPNYLGEMVEWLGWAVMTWSWAGF
AFFLYTCANLGPRARAHRKWYLEKFGEEFPASRMAVVPFVY*

>*D._alata*_DET2
MAWDDEKIFSKALISLYIMCPLTILSLHFLTAPYGKHARSGWGPTLPPCLAWFLMESPTLWLTVLLYPLGRY
SSHPLSLIVISLYLLHYTHRTLIYPLRLLSTSTSKSNLGFPLSIAFFAFSFNLLNAYLQARSASHYAGSAMAED
GWRLWFRVAAGMAVFFLGMRVNIRADLALVKLKKEGGGYKIPRGGWFEFVSCPNYMGEALEWLGWAIV
AWSPASWAFFLYTCSNLGPRARAHHKWYCEKFGSDYPSSRKAIVPFFY*

>*A._officinalis*_DET2
MTIVSFTSLAYTGLSEIRGKHLHYSKFWNVGNSSENKKIENEIKISSRVGMLLFYGPAAAAAAASFFVPGLV
VGLRAQLLSWALLVQFFKRVLEVLFIHQYSGQILLSSAVPITSSYLSGTVSMIYTQYLSQNMTQPSIDLKYPG
LVLFLIGIAGNFYHHVLLSKLRQKNEKGYKIPKGGLFGLIICPHYLFEIIAFYGITLIAQTMYALSFALGTTVY
LIGRSFATRKWYLSKFERFPREVKALIPYIF*

>*C._nucifera*_DET2
MESSLSDETLFRRALVSLYVMCPLTVLFLRFLVAPYGRHARSGWGPALPPALAWVLMESPTLWLTLLLLPR
GRHRSHPFALTILALYLLHYIHRTLIYPLHLSINSRSKSGFPLVIALSAFSFNLLNAYLQARSASHYADYPAAA
AFGLWVWLRVLVGMGIFFWGMSVNIRSDLALLRLKAEGGGYKIPRGGWFEWVTCPNYMGEAAEWLGW
AVVAFSPAALAFFLYTCSNLVPRARAHHQWYLQKFGEDYPRSRKAIVPLVY*

>*Z._officinalis*_DET2
MEGFEDSDLFSIALLSLYVMCPLTVASLQFQTAPYGKHVQPGWGPSLPAPLAWFLMESPTIWLTLLLYPHG
RNRSHPVALAVLSLYLLHYAHRTLIYPLRLHFSSRKATGFPLLIAFFAFAFNLLNAYLQSRSISHYADYTSQR
WAWPRAAVGAAIFFWGMAINISSDLALVRLKAKGEGYKIPRGGWFELVSCPNYTGEMMEWLGWAVMA
WSPAALAFFLYTCSNLGPRARASLQWYRAQFGDAYPKSRKAFVPFIY*

A. officinalis DET2	100.00	29.91	27.62	29.91	29.38	26.05	28.04
S. cereale DET2	29.91	100.00	49.40	52.94	53.10	51.36	55.60
A. americanus DET2	27.62	49.40	100.00	57.60	56.97	58.33	57.77
***S. polyrhiza* DET2**	**29.91**	**52.94**	**57.60**	**100.00**	**63.14**	**63.18**	**67.95**
Z. officinalis DET2	29.38	53.10	56.97	63.14	100.00	68.09	68.99
D. alata DET2	26.05	51.36	58.33	63.18	68.09	100.00	71.15
C. nucifera DET2	28.04	55.60	57.77	67.95	68.99	71.15	100.00

CYP90C1

>*A._thaliana*_ROT3
MQPPASAGLFRSPENLPWPYNYMDYLVAGFLVLTAGILLRPWLWLRLRNSKTKDGDEEEDNEEKKKGMIP
NGSLGWPVIGETLNFIACGYSSRPVTFMDKRKSLYGKVFKTNIIGTPIIISTDAEVNKVVLQNHGNTFVPAYP
KSITELLGENSILSINGPHQKRLHTLIGAFLRSPHLKDRITRDIEASVVLTLASWAQLPLVHVQDEIKKMTFEI
LVKVLMSTSPGEDMNILKLEFEEFIKGLICIPIKFPGTRLYKSLKAKERLIKMVKKVVEERQVAMTTTSPAND
VVDVLLRDGGDSEKQSQPSDFVSGKIVEMMIPGEETMPTAMTLAVKFLSDNPVALAKLVEENMEMKRRK
LELGEEYKWTDYMSLSFTQNVINETLRMANIINGVWRKALKDVEIKGYLIPKGWCVLASFISVHMDEDIYD
NPYQFDPWRWDRINGSANSSICFTPFGGGQRLCPGLELSKLEISIFLHHLVTRYSWTAEEDEIVSFPTVKMKR
RLPIRVATVDDSASPISLEDH*

>*A._americanus*_ROT3
MAKFHIYSLSLRIYLYLYLNQVSFVEGVSKNHHELLRMMMSWAWVGFWLLVAIVGGWYWVWWLRKKE
RERKRGVPDGSLGWPFIGETLDFISSGQSSKPVPMRFMDKKRSLYGKVFKSHILGLPMIVSMDGEVNKAVL
QNDGRSFVPYYPKTIMELFGELSIITMEGNLHRHMHGLVGGFLKSPALKTRFAREFEGRIRAVMAEWEVVG
GGGRVVHFQDVAREITFQMLVRVLLGIEPGERYEHLKYEFHEFIKGLICLPVKFPGTRLYKSLKAKARMVK
LVKGIIEDKMKRVDNESNTDVLDVLLNEMNGGVTGQKISIDFVCNNIIEMMVPAQDSVPTLLTLAVKYLSD
HPLALQHLLKENMEVKKEKMRSGEPYAWSDYMSLRFTQNVISETLRMGNIINAVWRKALKDVNIKGYLIP
KGWGILTSFSSIHLDEENYENPYKFNPWRWEKKEGNPLNFTPFGGGQRLCPGGDFSRLEVAIFLHHLVTRYS
WVAEEDTIISFPTVRMAKRMPIKLSPISTDQIK*

CYP90D1

>*A._thaliana*_CYP90D1
MDTSSSLLFFSFFFFIIIVIFNKINGLRSSPASKKKLNDHHVTSQSHGPKFPHGSLGWPVIGETIEFVSSAYSDR
PESFMDKRRLMYGRVFKSHIFGTATIVSTDAEVNRAVLQSDSTAFVPFYPKTVRELMGKSSILLINGSLHRR
FHGLVGSFLKSPLLKAQIVRDMHKFLSESMDLWSEDQPVLLQDVSKTVAFKVLAKALISVEKGEDLEELKR
EFENFISGLMSLPINFPGTQLHRSLQAKKNMVKQVERIIEGKIRKTKNKEEDDVIAKDVVDVLLKDSSEHLT
HNLIANNMIDMMIPGHDSVPVLITLAVKFLSDSPAALNLLTEENMKLKSLKELTGEPLYWNDYLSLPFTQK
VITETLRMGNVIIGVMRKAMKDVEIKGYVIPKGWCFLAYLRSVHLDKLYYESPYKFNPWRWQERDMNTSS
FSPFGGGQRLCPGLDLARLETSVFLHHLVTRFR*

***>S._polyrhiza*_CYP90D1**
**MGVVIGCYCEAVRMDLSFSLAIFHFASEGNALKAIELTEELAPNLLEQNKDLHFDLLSLRFVELVRSR
KCTEALEFAQSMLTPFGKVQKYVEKLEDFMALLAYEEPEKSPMFHLLSSEHQQNVADNLNQAILGD
EGLAAWSLSDASSVNLLPKEGCRLSPRYGRVFRTHILGKAIAVSTDAEVNRAVLSSDGFVPCYPRSVA
ELLGKDSILKAEGALHRRVHGLIGGFLKSPAAKAAACAEVERCILRSLADWPGKGLVLVQEETQKIT
FPPLVKVLMGMGPGEELDIMKEQFHEFIQGLICLPIKLPGTRLSKSLKAKEKVQNIIDKIVEDKIQAG
EPNVVKDVVDVLINEIVTNKFPEMPISLISSIITEMMVPGEHSVPMVMTLAVKYLTDTPLALKQLREE
NLTLRRQKVKSGEPYTWADYMSLTFTHHVINETLRMGNIVNGVWRKAIRDIEVKGYLIPKDWCILAS
FSSVHFDEENYSNPYKFNPWRWEANGICVSKLTPFGGGQRLCPGQEISRLEISIFLHHLVTKYSWVAE
EDTIVSFPIVKMKRKMPIRVSPVS***

>*S._cereale*_CYP90D1
MDYLAIVVALVVAASSIAVHLLSRAKKARPGNLPPGSLGLPVIGQSLGLLRAMRGGDGGSRWVQDRIDRY
GPVSKLSLFGTPTVLLAGPAANKFLFFSSALSTRQPRSVQRILGENSILGLHGADHRRVRGALLEFLRPDML
KMYVGAIDGEVRRHVEENWAGRGTVTVLPLMKRLTFDIISALLFGLERGAVRDALAGDFAHMMEGMWAI
PANLPFTAFSRSLKASGRARRVLAGITQEKKASRRQPEHGKASRSNDLISCLLGLTDSHGERLLSDEEIVDN
AMVALIAGHDTSSILMTFMVRHLANDDATLAAMVQEHEEIAKNKGDGEALTWEDLTKMKYTWRVAQEI
LRIVPPIFGNFRRALEDVEFDGFLIPKGWQVFWTANVTHMDASIFREPARFDPSRFESQAASAAPPCSFVAFG
GGPRICPGIEFSRIETLVTMHHLVRQFRWKLCCKENTFVRDPMPSPLRGLPIQIELRTSPPP*

>*A._americanus*_CYP90D1
MYGLYIISVFLLTMLLYNFTRSQRLSMLFEMDYKLMIGLLAVMISSITAMLYESWKRVTRGRDHGLPPGDM
GWPLIGETLEFVSCAVSPRPESFMDRRRAMYGKVFKSHIFGSATIVSTDEEVNRYVFQSDAKTFVPSYPRSI
MELMGKSSILLINGSLQRKIHGLVGAFFKSSQHKSQIPVYMEKYIKEAMDSWKNGQLIHIQEVTKHIAFQVL
VKSLINLDPGEDMQFLKKQFQEFNAGLMSLPIKLPGTRLYRSLQAREKMIKLIRKIIEERRRISEATYFTTPRD
VLDVLLNDNGDQLTDDLILDNMIDMMIPGEDSVPVLMTLSVKYLSDCPLALKQLEEENMELHRQKARLGE
QLDWNDYMSLSFTQNVITETLRMGNVIIGAMRKAMKRVEIKGYTIPKGWCVFMYFRSVHLDGNNYADPY
KFNPWRWQDKDIGTCGFTPFGGGKRLCPGMDLARLETSIFLHQFVDRFRWVAEDDSIINFPTVRMKKRMP
VRVHKKEEKNLNNLVETINKQSYKQ*

>*D._alata*_CYP90D1
MDFNNKVCMTLFFSIIILLTFMSTRFNKRKRSSRKNLSRSAKLPSGSLGWPFFGETLEFISCAYSPHPESFMN
KRRLLYGKVFKSHIFGSPTIVSTDAEVSRVVLQSDAKAFVPSYPKSLTELMGKSSILLINGGLHRRVHGLIGA
FFKSPLLKSQITTDMHKYVQHLMSGWQDNQLIFIQDETKHIVFQILVRVLIGLEPGEEMQFLKHNFQEFIAGL
MSLPVKLPGCRLYKSLQAKKRMVMLVEKIIREKKKKRIMMMMMNSNTSDHQNTPRDVIDVLLNDNSNQL
NDDLISDNMIDLMIPGEDSVPILVTLAIKYLSDCPPALKQLEDENMQLKQQKSSLSWTDYLSLTFTQDVITET
LRLGNIISGIIRMAMKDMEIKGHFIPKGWCVFTYFRSVHLDETIHENPHKFNPWRWKDKDMNSCGFTAFGG
GQRLCPGLDLARLEASIFLHHLVTNFTWVAEEDQVVNFPTVRMKRRMPIRVKRKVET*

>*A._officinalis*_CYP90D1
MERALSISLAILLIPLTLVTLSKISKIKRWWRTRSKLPRGSSGWPFIGETLDFIACAFSPCPESFMSKRRLMHG
KVFRSHLFGSPTIVSIDAEFSRAVLQSDAKAFVPWYPKSLTELMGKSSILLINGSLQKKVHGLIGAFFKAHHL
KAQITSDMQSYVQQSMAKWEDGQLVHIQDESKQIVFQILVKVLIGLEPGEEMHLLKVQFREFIAGLMSLPV
KFPGSRLYRSLKAKKNMVKLVEKIINEKRERNNGKFARDVIDVLINDASGQLTDDLISDNMIDLMIPGEDSV
PVLITLAIKYLSDCPLALQRLEEENMQLKKKKSQLGESLDWTDYMSLSFTQDVITETLRLGSIISGIMRTAVK
DTEVKGHFIPKGWCVFMYIRSVHLDESHYDDPHQFNPWRWKNKDLSTCSFTPFGGGQRLCPGLDLARLEA
SVFLHHLVTNFTWVAEKDYVVNFPTVRMKYRMPVKITRRRDV*

>*C._nucifera*_CYP90D1
MDNLFITWLATTAIVLATILLYTSWNKLIRSRRPRRAQLPRGTFGWPLLGETLDFVSCAYSPRPESFMDKRR
LLYGKVFKSHIFGSPTIVSTDAEVSRFVLQSDAKTFVPWYPKSLTELMGKSSILLINGSLQKKVHGLIGAFFK
SPHLKAQITRDMQSYVLEAMSHWKDDQLIHIQDESKRIVFQILVKGLIGLEPGEEMNFLKQQFREFIAGLMS
LPLKLPGSRLYKSLQAKKRMVMLIEKIIQEKRKIKNGCTQRDVVDVLINDASDQLTDDLISDNMIDLMIPAE
DSVPVLVTLAIKYLSECPPALQYLEEENMNLKKQKSVTGENLQWTDYMSLSFTQDVITETLRMGNIISGIMR
KAVKDVEIKGHFIPKGWCVLTYFRSVHLDESLYKEAYKFDPWRWKDKDMGTCSFTPFGGGQRLCPGLDL
ARLEASIFLHHLVTGFTWVAEEDRIINFPTVRMKGGMPIRVRRKTEESPFPG*

>*Z._officinalis*_CYP90D1
MEELTNCLIIISFFTFIILLLHQRRRGSSSSSSRGKISGKLPAGTDGWPFVGETLDFISCAYSPRPETFIDKRRSR
HGKVFRSHLFGSPMIVSADADVTKCVLQSDSRSFVPSYPRSLTELMGKSSILVINGSLQRRVHGLIGAFLKSP
AVKVRLAGDMQRYVRQSMAGWEDGQVVRIQDEAKNIIFRILVKGLIGLEAGKEMELLKQEFKEFIAGLMS
LPMKIPGSQLYKSLRAKKKMVRVIKSIIDEKRRRNLQTSAGASTPTDVVEVLINDSGDSDRWITDDLISDNV
VDLMIPAEDSVPVLVTLAVKFLGDSPIALQQLQEENMFLKEQKKLAGEDLQWNDYMSLSFTQHVITETLRV
GNIISGIMRKAVRDVEIKGHLIPKGWCVFTYFRSVHLDEAHFDEPYKFNPWRWKDKEYMSSCNFTPFGGGQ
RLCPGMDLARLEASIFLHHLVTNFTWVAEEDQIVNFPTVRMKKKMPIRVRRRSDIK*

S. cereale CYP90D1	100.00	26.86	29.42	27.25	29.09	27.55	28.39
S. polyrhiza CYP90D1	26.86	100.00	47.31	46.22	46.74	44.57	47.19
Z. officinalis CYP90D1	29.42	47.31	100.00	58.61	64.93	66.60	68.99
A. americanus CYP90D1	27.25	46.22	58.61	100.00	64.06	62.34	67.51
D. alata CYP90D1	29.09	46.74	64.93	64.06	100.00	71.22	74.52
A. officinalis CYP90D1	27.55	44.57	66.60	62.34	71.22	100.00	74.68
C. nucifera CYP90D1	28.39	47.19	68.99	67.51	74.52	74.68	100.00

CYP985A

>*A._thaliana*_BR6ox
MGIMMMILGLLVIIVCLCTALLRWNQMRYSKKGLPPGTMGWPIFGETTEFLKQGPDFMKNQRLRYGSFFK
SHILGCPTIVSMDAELNRYILMNESKGLVAGYPQSMLDILGTCNIAAVHGPSHRLMRGSLLSLISPTMMKDH
LLPKIDDFMRNYLCGWDDLETVDIQEKTKHMAFLSSLLQIAETLKKPEVEEYRTEFFKLVVGTLSVPIDIPGT
NYRSGVQARNNIDRLLTELMQERKESGETFTDMLGYLMKKEDNRYLLTDKEIRDQVVTILYSGYETVSTTS
MMALKYLHDHPKALEELRREHLAIRERKRPDEPLTLDDIKSMKFTRAVIFETSRLATIVNGVLRKTTHDLEL
NGYLIPKGWRIYVYTREINYDTSLYEDPMIFNPWRWMEKSLESKSYFLLFGGGVRLCPGKELGISEVSSFLH
YFVTKYRWEENGEDKLMVFPRVSAPKGYHLKCSPY*

>*S._polyrhiza*_BR6ox
**MQEGPRNPTNTGMWAYKPANARLLKRGVPDKSMSSPRCRQTLFMRWNELRHWRKGLPPGTMGW
PVVGETIKFLRGGPDFLQKQTGRYGSLFTSHLLGSPTVMSTDQEVNRWILMNEGKGIVPGYPRAMA
EILGEWNITAVHGAVHRVVRGAMLSAVGPTAVRERLFPTLDKFIGSRLCNWGDGGATVDVQAKARE
MMFLSALKLITGIETGKLSHLLPLPLSSSSSSSGHSDGFTLQGRKRITSLLRELIEERRRSPSAPKGGGD
ILNLLLQEEEEGEEEQEVHRKRPKLTDDQMIDLLVSIMYTGFETVSSAATMTVKYLHDHPSALQELR
KEHSEIRRRKSSPDEALAWGDYTSMGFTRAVILETMRLATIVNGVMRKTIEDIPIKGFVVPKGWKINV
SIRDSNYDSLRYPAPLCFDPWRWKAMVEYSYPSASGRGRSASSLL***

>*S._cereale*_BR6ox
MALLLLLLAGVVAGVVLASSLLLRWNELRYGNGRRKEGDGCLPPGTMGWPLFGETTEFLKQGPAFMKQR
RLRYGRLFRTHILGCPTVVCMDPELNRRMLLQGEAGGLVPGYPQSMLDILGRNNIAAVHGPLHRVMRGA
MLGLVRPAMLRQSLLPKIDAFVRDHLHGWAGSVVDVQAKTKEMALLSALRQIAGITAGPLSDALKTELCT
LVLGTISLPINLPGTSYYQGFQARTKLVSMLEQMITERRSSGDAHDDMLDALLRSGDDGTREKLTDEQIIDL
LIALIYSGYETMSTTSMMAVKYLSDHPRALDELRREHLDIRKGKSPEEAISYDDFKSMAFTRAVIFETLRLA
TVVNGLLRKTTKDVEMNGYVIPEGWRIYVYTREINYDPFMYPDPMTFNPWRWLEKNMESHPHFMLFGGG
GRMCPGKEVGTAEIATFLHYFVTRYRWEEEGKNTILKFPRVEAPNGLHIRVQDY*

>*A._americanus*_BR6ox
MGVLVAVIGVVLVLSVCSFLLKWNEVRYRKKGLPPGTMGWPLFGETTEFLKQGPNFMKSQRTRYGNLYK
THILGCPTVICMDPELNRYILMNEGKGLVPGYPQSMLDILGKYNIAAVHGSLHKTMRGAMLALIGPTMIRE
QLMPKIDEFMRSHLHNLNGEIIDIQDKTKEMAFMVSLKLIGGIESGLLSETFKPEFYRLVEGTLSLPIDLPGTN
YHHGFQARKKITSMLNEIINERRNTSTACGDMLDALLKTDEVARAKLTDEQIVDLILTIIYSGYETVSTTSM
MAVKYLHDNPKVLEELREEHFAIRKGKSPEDPIDWNDYKSMNFTRAVIYETLRMATIVNGLLRKTTQDME
MKGYVIPKGWKIYVYMRESNYDPFLYPEPLTFNPWRWLDKDLESHQCFLLFGGGGRLCPGKELGTTEISVF
LHYFVTRYRWEEVGNDKLVKFPRVEAPNGIRVRISDY*

>*D._alata*_BR6ox
MLPIYSQANTLLLLTATRRRRRRKEEVELWNSVMVVFGGVLLLILGLVFSFLVISSAVLRWNELRYRKKGL
PPGTMGWPLFGETTEFLKQGPNFLKNQRARYGSLFKSHILGCPTVVSMDPELNRFILMNEGKGLVPGYPQS
MLDILGKCNIAAVDGPLHKAMRGAMLGLINPPMIRDQLLHKIDQFMRSHLTDWSNKIIDIQQKTKEMALLS
ALKQIASIETGSFAESLKTEIFKLVVGTLSLPINLPGTNYRRGLQARKKAVGMLRDLIQQRRESNCSQNDML
DCLLNNDGNTRSMLSDDQIIDLIIALVYSGYETVSTTSMMAVKYLLDHPKALEELRKEHFGIRNGKASEEGI
DWNDYKSMRFTRAVILETLRVAAVVNGVLRKTTHDMEMKGFLIPKGWRIYVYTREINYDQYLYPEPATFN
PWRWLDKNLDSHPYFLLFGGGSRLCPGKELGIAEIATFLHYFVTRYRWEEVGGNHIVKFPRVEAPNGLRIR
VFNY*

>*A._officinalis*_BR6ox
MVILGAFFGLLVAFLIICSALLKWNELRYRRKGLPPGTMGWPLFGETTEFLKQGPSFMKNQRARYGNLFKS
HILGCPTVMWLFSALKLVAGIESGQFSKAMGSEINKLIIGTLSLPINLPGTSYHQGLQARKKIVSMLERIIEER
RASKRTSNDMLDALLFSNDDSRANLDDEQLMDLIITFIYSGFETVSTTSMMAVKYLHQHPKVLKELVKEHV
EIRRRKLPDEAIDWNDYKSMTLTRAVIIETSRLATVVNGVLRRTTQDMEIKGYTIPKDWKIYVYIREINYDA
FQYPDPFTFDPWRWLGKNQESHPYFMAFGGGTRLCPGKELGIVEIAVFLHYFVTRYRWEEVGGDKLVTFP
RVEAPNGLHIRVLER*

>*C._nucifera*_BR6ox
MLGNSCLSAAMVLLGWVLGLALGFLVVCSGLLRWNEVRYRKKGLPPGTMGWPLFGETTEFLKQGPSFMR
NQRLRYGSLFKSHILGCPTVVCMDPELNRFILMNEGKGFVPGYPQSMLDILGKWNIAAIHGSLHKAMRSA
MLGLINPSVIRDQLLPKIDEFMRSHLCSWNGKVIDIQEKTKEMALLSALKQIASIENGPLSEALKTVIFQLVL
GTLSLPINLPSTNYRRAFRARKKLVGMLREIIEERRACQCSYNDMLDSLLKNDDSTKVKLTDEQIVDLIIALV
YSGYETVSTTSMMAVKYLHDHPRALEELRNEHLKIRKGKSAEDAIDWNDYKSMSFTRAVIFETLRIATVVN
GVLRKTTQDVEMKGFVIPKGWRIYVVYTREINYDPFLYPEPLTFNPWRWLDKHLESHQHFMMFGGGGRLCP
GKELGTAEIATFLHYFITRYRWEEVGGDKILKFPRVEAPNGLHIRVWDY*

>*Z._officinalis*_BR6ox
MVVLVMLPLLCFLFICGALLRWNEVRYRKKGLPPGTMGWPLFGETTEFLKQGPSFMKNQRVRYGSVFKSH
ILGCPTMVCMDAELNRFILMNEGKGFVPGYPQSMLDILGRSNIASVHGELHKTMRSAMLGLVSPPMIRDQL
LPKIDEFMRSYIHNWGGRVIDIQEKTKEMALLSSLKQIASIETGPVSEALKSEIFKLVLGTLSLPINIPGTNYH
QGFKARKRLVGMLRCLIEERRTSSCSYNDMLDSLLRMDGSSKVKLDDDQIVDLIIALVYSGYETVSTTSMM
AVKYLHDHPRVLEELRNEHFEIRKGKSREDAIDWNDYKSMKFTRAVILETLRMATVVNGVLRKTTRDVSL
KGFTIPKGWRIYVVYTREINYDPDVYQEPLAFNPWRWLDKNLESNHHFMLFGGGGRMCPGKELGTAEIATF
LHYFVTRYRWEEVGGDEIVKFPRVEAPNGLRIHVWDD*

***S. polyrhiza* BR6ox**	**100.00**	**48.05**	**49.68**	**49.88**	**46.62**	**48.09**	**48.52**
S. cereale BR6ox	48.05	100.00	64.61	66.59	68.24	71.89	68.47
A. officinalis BR6ox	49.68	64.61	100.00	68.28	68.90	69.17	67.92
A. americanus BR6ox	49.88	66.59	68.28	100.00	70.47	74.57	71.21
D. alata BR6ox	46.62	68.24	68.90	70.47	100.00	77.26	76.03
C. nucifera BR6ox	48.09	71.89	69.17	74.57	77.26	100.00	81.86
Z. officinalis BR6ox	48.52	68.47	67.92	71.21	76.03	81.86	100.00
