# Supplementary material for: Duckweeds: Model organisms for research on plant sterols and steroids
Source: Plant Biol (Stuttg). 2025 Aug 25;28(1):18–30. doi: 10.1111/plb.70095 (PMC12710849; doi:10.1111/plb.70095)
Supplement: Supplementary file 4 — Data S4. Brassinosteroid signaling. [file PLB-28-18-s003.pdf]

BRI1

>*A._thaliana*_BRI1
MKTFSSFFLSVTTLFFFSFFSLSFQASPSQSLYREIHQLISFKDVLPDKNLLPDWSSNKNPCTFDGVTCRDDKV
TSIDLSSKPLNVGFSAVSSSLLSLTGLESLFLSNSHINGSVSGFKCSASLTSLDLSRNSLSGPVTTLTSLGSCSG
LKFLNVSSNTLDFPGKVSGGLKLNSLEVLDLSANSISGANVVGWVLSDGCGELKHLAISGNKISGDVDVSR
CVNLEFLDVSSNNFSTGIPFLGDCSALQHLDISGNKLSGDFSRAISTCTELKLLNISSNQFVGPIPPLPLKSLQY
LSLAENKFTGEIPDFLSGACDTLTGLDLSGNHFYGAVPPFFGSCSLLESLALSSNNFSGELPMDTLLKMRGL
KVLDLSFNEFSGELPESLTNLSASLLTLDLSSNNFSGPILPNLCQNPKNTLQELYLQNNGFTGKIPPTLSNCSE
LVSLHLSFNYLSGTIPSSLGSLSKLRDLKLWLNMLEGEIPQELMYVKTLETLILDFNDLTGEIPSGLSNCTNL
NWISLSNNRLTGEIPKWIGRLENLAILKLSNNSFSGNIPAELGDCRSLIWLDLNTNLFNGTIPAAMFKQSGKI
AANFIAGKRYVYIKNDGMKKECHGAGNLLEFQGIRSEQLNRLSTRNPCNITSRVYGGHTSPTFDNNGSMMF
LDMSYNMLSGYIPKEIGSMPYLFILNLGHNDISGSIPDEVGDLRGLNILDLSSNKLDGRIPQAMSALTMLTEI
DLSNNNLSGPIPEMGQFETFPPAKFLNNPGLCGYPLPRC
DPSNADGYAHHQRSHGRRPASLAGSVAMGLLFSFVCIFGLILVGREMRKRRRKKEAELEMYAEGHGNSGD
RTANNTNWKLTGVKEALSINLAAFEKPLRKLTFADLLQATNGFHNDSLIGSGGFGDVYKAILKDGSAVAIK
KLIHVSGQGDREFMAEMETIGKIKHRNLVPLLGYCKVGDERLLVYEFMKYGSLEDVLHDPKKAGVKLNW
STRRKIAIGSARGLAFLHHNCSPHIIHRDMKSSNVLLDENLEARVSDFGMARLMSAMDTHLSVSTLAGTPG
YVPPEYYQSFRCSTKGDVYSYGVVLLELLTGKRPTDSPDFGDNNLVGWVKQHAKLRISDVFDPELMKEDP
ALEIELLQHLKVAVACLDDRAWRRPTMVQVMAMFKEIQAGSGIDSQSTIRSIEDGGFSTIEMVDMSIKEVPE
GKL*

>*S._polyrhiza*_BRI1
MRQPPTDLQQLLSFKSALPDPARSLPSWQPGGDPCASFAGVSCRDSRVSALRLDGVAINGDFFHVSSSLLSL
EFLETLSLKGTNLSGTLLPPPPPGPRCGGRLIELDLARNVLRGSVTEIPSLTSWCPSLRTLNLSGNALVFSPSG
AGKVAGLGLNSVDLSFNRIAGEDALDWLLLSGSDLRALNLSGNNITGSVPTLRSCPELQQLDISSNSLSAIAP
AAFAACSSLSVLNLYNNNFAGELPGSLFGDCRSLLSLDLSSNNLSGTVPSEFASCVSLQSINLSNNNFSGYLP
AETLTAMAGLRKLELSFNYFSGRLPDILSKLPELEVLDLSSNSLSGSIPAGLCRRPGLRLKELYLQSNQLTGA
VPPAISNCSMLVSIDLSYNYLRGPIPSSIGSLYHLRDLIMWQNQLQGEVPEEISHLKQLENLILDDNDLTGPLP
EGLSNCTNLNWISLSSNRFSGKIPPWIGGFPNLAILNLGNNSFAGEIPPEIGDCRSLIWLDLHSNELNGSVPPE
LAKQSGKIAVGLVTGKRYVYLRSDGGSDCRGAGNLLEFAGIRPEQLSRVSTRRSCNYTRAYVGNTQYTFN
NNGSILFLDLSYNQLGGQLPPELGAMFYVMVLNLGHNAFSGIIPPELGKLGRVCILDLSHNALEGPIPSSFSG
LAMLSEIDLSNNRLNGTIPVGGQLATFSAAVYLNNSGLCGLPLPPCGGDGAASGEGQRRRSHRKQASLAAS
VAMGLLFSLFCVFGLILVAVERKKRQEKKKKKKTMNKDGTNGGGRDPYMDSGSHSGTCNSNWKLTAT
KEALSINLATFEKPLRMLTFADLLEATNGFHDDTLVGSGGFGDVYRAQLKDGSVVAIKKLIHLSGQGDREF
TAEMETIGKIKHRNLVPLLGYCKVGDERLLVYEYMKHGSLEDVLHSRKKKSGGFKLDWPARRKIAVGAA
RGLAFLHHNCIPHIIHRDMKSSNVLLDENLEARVSDFGMARLMSAMDTHLSVSTLAGTPGYVPPEYYQSFR
CSTKGDVYSYGVVLLELLTGKQPTDSPEFGDGNLVGWVRQHTKLRLSDVFDQELLREDPTLELELLKHLKI
ACACLDDRPMRRPTMLKVMAMFKEIQVGLAVNSLDAGYDMALKEGREDKDQ*

>*S._cereale*_BRI1
MDSLRLAIAAALFLAALAAAADDVQLLDDFRAAVPNREALEGWSARDGACRFPGAVCRGGRLTSLSLAA
VALNADFRAVAATLLQLSAVERLSLRGANVSGALAAAAGARCGTKLQELDLSGNAALRGSVADVAALAA
SCGGLRTLNLSGGAVGAAKAAGGGGGGQGFAALDALDLSSNKIAGDADLRWMVGAGLGSVRWLDLAW
NKISGGLSDFTNCSGLQYLDLSGNLIAGDVAAGALSGCRSLRALNLSSNHLAGAFPPNIAGLTSLTALNLSN
NNFSGEVPADAFTGLQQLQSLSLSFNHFSGSIPDSVAALPDLEVLDLSSNDFSGTIPSSLCQDPNSRLRVLYL
QNNYLSGTIPEAVSNCTDLVSLDLSLNYINGSIPESLGELGRLQDLIMWQNLLEGEIPASLSSIPGLEHLILDY
NGLTGSIPSELAKCKQLNWISLASNRLSGPIPSWLGKLSNLAILKLSNNSFTGQIPAELGDCKSLVWLDLNSN
QLNGSIPPELAEQSGKMTVGLIIGRPYVYLRNDELSSQCRGKGSLLEFSSIRSEDLGRMPSKKLCNFTRMYM
GSTEYTFNKNGSMIFLDLSFNQLDSEIPKELGNMYYLMIMNLGHNLLSGAIPTELAGAKKLAVLDLSHNRL
EGPIPSSFSSLSLSEINLSSNQLNGTIPELGSLATFPKSQYENNTGLCGFPLPPCESHTGQGSSNGGQSNRKKAS
LAGSVAMGLLFSLFCIFGLVIIAIESKKRRQKNDEASTSRDIYIDSRSHSGTMNSNWRLSGTNALSINLAAFE

KPLQKLTLGDLVEATNGFHNDSLIGSGGFGDVYKAQLKDGRVVAIKKLIHVSGQGDREFTAEMETIGKIKH
RNLVPLLGYCKIGEERLLMYDFMKFGSLEDVLHDRKKIGIRLNWAARRKIAIGAARGLAFLHHNCIPHIIHR
DMKSSNVLVDENLEARVSDFGMARMMSVVDTHLSVSTLAGTPGYVPPEYYQSFRCTTKGDVYSYGVVLL
ELLTGKPPTDSTDFGEDHNLVGWVKMHTKLKITDVFDPELLKDDPTLELELLEHLKIACACLDDRPSRRPT
MLKVMTMFKEIQAGSTVDSKTSSVATGLSDDPGFAVMDMTLKEAKEEKD*

>*A._americanu*s_BRI1
MALPFLFLLLTLTTTSAADSHPLISFKSSLPNPNVLSDWLPGRSICSYSGVLCGDSGSLSVDLHSLPLDADLK
SVSPLLAIDGLERLSLRSSNLSGDIDVLSGSRCTGSLHELDLSGNSVSGFATAIEGIGCSSLVTLNLSGNSLAFP
ANAGAGGRLGVEVVDLSVNKISGGGVLKWALSAGGVRRLNLSRNAISGELPAIDLPAMEDLDLSFNNISGG
LPHSVNCSALLRLDLSSNSLSGDVPSAAFASCFRLTSLNLSSNHLSGELPSVPGTLSSLLLAENEFSGRLSDCH
GLVDLDVSSNHLQGPIDFSDCPSLVTINLSNNNFSGEIPFETLNTLQNLKSLILSYNSFSGSLPDSISDLYRLEL
LDLSSNNLTGQIPQTLCGNPNFVLKELYLQDNLFTGTVPASLANCSGLISLDLSFNFLKGPIPSTFGSLSRLRD
LIMWLNLLDGEIPQELSMIQTLENLILDNNGLVGTIPSGLQNCTNLNWLSLSSNELSGPIPPWIGRMSNLAIL
KLGNNSFSGPIPPEIGDCRSLVWLDLNSNQLNGSIPITIAKQSGKIAVGLVTGKRYVYLKNDGSAECRGAGN
LLEFAGIRPEQLNRVPTRRSCNFTRVYMGSTKYTFNNNGSMIFLDLSYNHLDQPIPEELGSMYYLMVLNLG
HNDLSGPIPVNLGSLKSVAVLDLSYNALEGPIPGSFSGMSMLSEIDLSHNRLNGSIPELGQLATFPAYRYENN
SGLCGFPLPPCEANANSTGMGRQKSHRRQASLAGSIAMGLLFSLFCIFGLIIIAVESRKRQRRKSKEVDGGG
GDHHSESRSYSASAWKFTGTREAMSINLATFEKPLRKLTFADLLEATNGFHSESLIGSGGFGDVYKAQLKD
GNTVAIKKLINVSRQGDKEFMAEMETIGKIKHRNLVPLLGYCKVGEERLLVYEYMRFGSLDDVLHDHKKL
GIKLDWPARRKIAVGAARGLAFLHHNCTPHIIHRDMKSSNVLLDNNLEARVSDFGMARMMSTADTHLSVS
TLAGTPGYVPPEYYQSFRCSTKGDVYSYGVVLLELLTGRQPTDSVDFGDNNLVGWVKQHARLKITDVFDR
ELMKGDPTLEMELLQHLKIACACLDDRPWRRPTMLQVMAMFKEIQVGSGVSAVSGSDGSLAEVVEGNEH
EKN*

>*D._alata*_BRI1
MDVLLILLVLLPLQLSFSAPQADLKHLISFKSSLPNPQTLPTWSSDGDPCASFSGVTCISGRVAVVDLTGVAL
TSDFHAVTSSLLALDSLQSLYLKSTNLTGTLAGGRCSGGQLAVLDLSENGLKSSLADVLSFAASCSSLTLLN
LSTNFVTGGGKGIAGAGAGAGTSFETLDLSFNQISDEEDLHQLLSLPGILRLNLTGNGLSGQIPVITNGSSLQ
HLDLSSNNLYGEIGAGVFAGCRSLLSLNLSSNHFTGTLPADLSSCFSLNSLSLSNNNFSGEIPVETLTSLPDLIT
LEFAFNNLSGKLPDTLSKLTKLELLDLSSNGFFGSIPSGLCQSPATSLRELYLQNNLFTGLVPPSLSNCSMLVS
LDLSFNYLHGAIPASLGSLSRLRDLIMWQNLLSGEIPSEFTLAQSLENLILDNNGLQGTIPEGLVNCSNLNWIS
LSSNKLSGPIPSWLGRLSNLAILKLGNNSFSGPIPPELGDCKSLIWLDLNSNSLNGTIPATLSKQSGNIAVGLV
TGKRYVYLKNDGSTECHGAGNLLEFAGIRPDQLSRLPSRRFCNFTRVYMGNTRYTFKNNGSMIFLDLSYNQ
LSGEIPRPLGSMYYLMILNLGHNLLSGSIPPALGGLRYVAVLDLSHNLLEGPIPSTFSGLAMLSEIDLSNNQL
NGSIPQLGQLATFPRYRYENNSGLCGFPLPSCDDGRSSGDNAQRRKSHRWQASLAGSIAMGLLFSLFCILGL
IIIAVESKKRRQRRKDISNVSTRDVDSRSHSGTATTAWKLTATKDAMNINLSTFDKPLRKLTFADLLEATNG
FHNDSLIGSGGFGDVYKAQLKDGSVVAIKKLIHISGQGDREFIAEMETIGKIKHRNLVPLLGFCRVGEERLL
VYEYMKYGSLEDVLRDRRRAGIKLNWAARRKIAIGAARGLAFLHHNCFPHIIHRDMKSSNVLLDDNLEAR
VSDFGMARMMNAVDTHLSVSTLAGTPGYVPPEYYQSFRCTTKGDVYSYGVVLLELLTGKQPTDSSDFGD
NNLVGWVKQHSKLRIIDVFDPELLKEDPSLELELLEHLKIACSCLDDRPLRRPTMLKVMAMFKEIQAGSTV
DSTPSATTTEGTTAFTVVDMKMNGGLKV*

>*A._officinalis*_BRI1
MDSLLLFFLMFIRVGFSSATSDLRLLLSFKESLPDPTVLSTWNPTRDPCSFSGVTCAPNSAVTSVAVDNVAL
NTDFNSVSTSLLLLPDLESLSIRSGNLTGGIVAAPRCSGQLTALDLSANLLTGSDVSALAASCSALTYLNLSY
NSVGEGGPVTAGELNLKLTALDLSYNRVSSGDLRLILSSSGGIGNLSLAGNKIAGYIPAITNCSGLRRLDLSF
NDFAGEIADGVFGDCYGLEFLNLSSNHLSGKIPFVSSLNSLYLSNNNFSGELPIETLASSPNLKNLDLAFNNIS
GRLLNSVSKLASLVLLDLSSNGLSGSIPDKLGPSLKELYLQNNKFTGPVPASISNCSNLVSLDLSFNYLTGKIP
ASLGELSQLRDLLMWQNLLVGEIPRELSKIRALENLILDNNGLTGEIPDGLQNCSELNWISLSSNHLTGEIPS
WIGKLNNLAILKLGNNSFYGSIPAELGDSRSLIWLDLNSNKLSGKIPGELAKQSGNIAAGLVTGKRYVYLKN
DGSSECRGAGNLLEFAGIKPEDLNRLPSRRTCNFTRVYMGSTQYTFNNNGSMIFLDLSYNELNGPIPKELGK
MYYLMILNLGHNLLSGAIPPDLGSLRYVAVLDLSHNELEGPIPGSFSRLMLSEIDLSNNQLNGTIPESGSLVT
FPRARYENNSGLCGFPLPSCDELQPKALDEHQKLRRGSLAGSVAMGLLLAFFCIFALVIVVVESRKRERRR
MKENENSNNLRDIYIDNSNSDSGYTTNKNWKLTGTKEAMSISLATFEKPLQKLTFQDLLDATNGFHDDSLI
GSGGFGDVYKAQLKDTSIVAIKKLIHISGQGDREFTAEMETIGKVKHRNLVPLLGYCRVGDERLLVYEYMK
YGSLEDVLHDRKKSGMKLNWAARRKIAVGAARGLAFLHHSCIPHIIHRDMKSSNVLLDENLEARVSDFGM
ARMMSAMDTHLSVSTLAGTPGYVPPEYYQSFRCTTKGDVYSYGVVLLELLTGKQPTDSPDFGDNNLVGW
VKQHCKLRIRDVFDPQLLKEDPTLELELLEHLKIACACLDDRPMRRPTMVKVMAMFKEIQAGNSGLGDDG
SISGWSYGGVDMSLKEGKRIKTEEKIFG*

>*C._nucifera*_BRI1
MSGRGGDLDGPTMDILPFLLLLLLLFLGLGLSESGDLELLISFKSSLPSPQVLRSWDTSQSPCSFAGVSCKAG
RVAGVVLQSLALSADFRSVSSSLLSLGSLELLSLRSANLTGSLSAAGSPCGGQLTVLDLADNALKGSVADV
YNLAAACTGLRSLNLSGNSIGIPPTGKNPSSGGGLSLETLDLSFNKISGEADLRLLLSSPALLRRLDLTGNQL
TGGIPSISNCSGLQHLDLSANEFAGDIMAGVFGGCRSLSYLNLSANHFTGTLPADLSSCSALTSLSLSNNNFS
GEFPFETLSSSLRNLEILDLSFNNFSGPLPNSVAKLPMLELLDLSSNGFSGSIPSALCQSHETSLEELYLQNNQF
TGRVPESLSNCSELVSLDLSFNYLTGAIPATLGSLSSLRDLIMWQNLLEGGIPARLSNIRSLENLILDNNGLTG
SIPAGLSSCTNLNWISLSSNHLSGPIPSWIGRLGNLAILKLGNNSFSGTIPPELGDCKSLIWLDLNNNQLNGTIP
PALARQSGNIAVGLVTGRRFVYLKNDGSSECRGSGSLLEFAGIRPEDLNRLPSRRFCNFTRMYMGSTQYTF
NDNGSMIFLDLSYNQLVGEIPRELGSMYYLMILNLGHNMLSGPIPSDLGNLHYVGVLDLSHNALQGPIPSSF
SGLSMLSDIDLSNNGLNGTIPELGQLATFPRYRYENNSGLCGFPLPSCEGSANANSSGQHRKSRRRQASLAG
SVAMGLLFSLFCIGLIIVAVETRKRKRRKKESNNDSRDFYVDNSRSHSGTANSNWKLTATKEALSINLATFE
KAPRKLTFADLLEATNGFHNDSLIGSGGFGDVYKAQLKDDSVVAVKKLIHVSGQGEREFMAEMETIGKIK
HRNLVSLLGYCKVGEERLLVYEYMKYGSLEDVLHDRKKAGIKLNWAARRKIAVGAARGLAFLHHNCIPHI
IHRDMKSSNVLLDENLEARVSDFGMARLMSATDTHLSVSTLAGTPGYVPPEYYQSFRCTTKGDVYSYGVV
LLELLTGRQPTDSSDFGDNNLVGWVKQHTKLRISDVFDTALLKEGPGLELELLEHLKIACACLDDRPLRRPT
MLKVMAMFKEIQAGTASAPLPSVDGSFGVVDMSLKEGKEEKD*

>*Z._officinalis*_BRI1
MCVFLTKPVLPGYTPCDPTLLPSCSCSSSSISSSSSLLQLSFFTSSSHSFPFRGLHCLPRSTALTEAEATAAMRR
RRLRVSSLFLLVFCFVMAPAAADDLELLIAFKASIPDPQILRTWSAGQSSPCSFAGVSCDSRGRVAALDLRG
VSLDAEFRAVASSLLPLAGLRRLSLRAVNLTGTLADGGAARCGRLLAEIDLADNQLRGSLADARGLAALCS
GLKSLDLSRNSIGGVLPPSAAAPAAGFGMLKSLDLSFNKVSGPDDLGWLFSSLAGLRRLDLVGNRLNAGIP
AIGNCSSLQHLDLSSTGLIGELGEGVFAGCQSLVYLNLSSNHLAGTLPSNISSSSSLTSVSLSNNNFSGEIPMA
TFASMPDLKSLELAFNYFNGGLGESIEEMPQLRLLDLSSNNLTGAIPLGLCANPSFALEELYLQNNRLSGSIP
ESLSNCTNLVSLDLSLNYISGTIPARLGSLYSLRDLILWQNSLEGEIPAELSSIPTLENLILDNNGLTGTIPAGL
ADCANLNWISLSSNRLSGPIPSWIGQLRNLAILKLDNNSFTGPIPPELGECKSLIWLDLNSNQLNGSIPPALAK
QSGKIAVGLVTGERFVYLRNDGISDRCRGTGSLLEFAGIRQDDLNRLPSHRFCNFTRVYIGSMGYTFNNNGS
MIFLDLSFNQLSGEIPSEIGQMYYLIILNLGHNSLSGLIPSELGNLRFVAVLDLSYNALEGPIPWSFSHLAMLS
EINLSNNKLNGTIPELGPLPTFSPNGYENNSGLCGLPLPPCQSHASVDTGAQNQSTHRRRAYVAGSVTMGVF
VSLFCIFGLVIVVVENRKRQRKDRGFDDNSRDIYIDSHSFSATGGISNWKLAALTKETVVINLTASEKPLMK
LTLADLVDATNEFHNDCLIGSGGFGDVYKAQLKDGNIVAVKKLIHVSGQGDREFTAEMETIGKVKHRNLV
PLLGYCKVEEERLLVYQYMKHGSLDDVLHKRNKGCIKLSWAARRKIAVGAARGLAFLHHNCIPHIIHRDM
KSSNVLLDDDSEARVSDFGMARMMNVVDTHLSVSTLAGTPGYVPPEYYQSFRCTTRGDVYSYGVVLLEL
LTGRPPTDSPEFGDNNNLVGWVKQHPRHRIRDVFDPELLAEQPSIEPELCEHLKIAYACLDERQMRRPTML
KVMAMFKEIQASGSTVETPATLKEEGTLVEEPHSSFKEEEDKFF*

A. americanus BRI1	100.00	66.45	62.30	66.00	61.91	67.78	68.36
A. officinalis BRI1	66.45	100.00	63.76	65.83	63.47	69.83	72.61
S. cereale BRI1	62.30	63.76	100.00	61.99	62.34	63.20	67.09
***S. polyrhiza* BRI1**	**66.00**	**65.83**	**61.99**	**100.00**	**61.33**	**66.98**	**68.42**
Z. officinalis BRI1	61.91	63.47	62.34	61.33	100.00	65.04	66.91
D. alata BRI1	67.78	69.83	63.20	66.98	65.04	100.00	74.84
C. nucifera BRI1	68.36	72.61	67.09	68.42	66.91	74.84	100.00

BAK1

>*A._thaliana*_BAK1
MERRLMIPCFFWLILVLDLVLRVSGNAEGDALSALKNSLADPNKVLQSWDATLVTPCTWFHVTCNSDNSV
TRVDLGNANLSGQLVMQLGQLPNLQYLELYSNNITGTIPEQLGNLTELVSLDLYLNNLSGPIPSTLGRLKKL
RFLRLNNNSLSGEIPRSLTAVLTLQVLDLSNNPLTGDIPVNGSFSLFTPISFANTKLTPLPASPPPPISPTPPSPA
GSNRITGAIAGGVAAGAALLFAVPAIALAWWRRKKPQDHFFDVPAEEDPEVHLGQLKRFSLRELQVASDN
FSNKNILGRGGFGKVYKGRLADGTLVAVKRLKEERTQGGELQFQTEVEMISMAVHRNLLRLRGFCMTPTE
RLLVYPYMANGSVASCLRERPESQPPLDWPKRQRIALGSARGLAYLHDHCDPKIIHRDVKAANILLDEEFE
AVVGDFGLAKLMDYKDTHVTTAVRGTIGHIAPEYLSTGKSSEKTDVFGYGVMLLELITGQRAFDLARLAN
DDDVMLLDWVKGLLKEKKLEALVDVDLQGNYKDEEVEQLIQVALLCTQSSPMERPKMSEVVRMLEGDG
LAERWEEWQKEEMFRQDFNYPTHHPAVSGWIIGDSTSQIENEYPSGPR*

>*S._polyrhiza*_BAK1
MEATWQQIIASGLVWLIVLFHPLTRVFANVEGDALHSLRTNLLDPNKVLQSWDPTLVNPCTWFHVT
CNNENSVTRVDLGNAALSGTLVPQLGQLTKLEYLELYSNNISGPIPSELGNLTNLVSLDLYLNNFSGPI
PETLGKLTKLRFLRLNNNSLSGPIPKSLTNISTLQVLDLSNNNLSGEVPSNGSFSLFTPISFNNNPLLCGP
GTAKPCPGAPPFSPPPPFVPQTTPPSPGSSASSTGAIAGGVAAGAALLFAAPAIGFAWWRRRKPQEHF
FDVPAEEDPEVHLGQLKRFSLRELQVATDGFSNKNILGRGGFGKVYKGRLADGSLVAVKRLKEERT
PGGELQFQTEVEMISMAVHRNLLRLRGFCMTPTERLLVYPYMANGSVASRLRERPPNEPPLDWATR
KRIALGSARGLSYLHDHCDPKIIHRDVKAANILLDEEFEAVVGDFGLAKLMDYKDTHVTTAVRGTIG
HIAPEYLSTGKSSEKTDVFGYGIMLLELITGQRAFDLARLANDDDVMLLDWVKGLLKEKKLDMLVD
PDLQNYVEAEVEQLIQVALLCTQSSPMERPKMSEVVRMLEGDGLAERWEEWQKVEVVRQEVDLAP
NRNSEWIVDSTDNLHAVELSGPR*

>*S._cereale*_BAK1
MAAAVLGRGRWRPVVVAVLMVVAGVGHVVANTEGDALYNLRQSLKDSNNVLQSWDPTLVNPCTWFHVTCN
NDNSVIRVDLGNAQLSGVLVSQLGQLKNLQYLELYSNNISGPIPAELGNLTSLVSLDLYLNKFTGVIPDSLGNLLK
LRFLRLNNNSMSGQIPKSLTDITTLQVLDLSNNNLSGAVPSTGSFSLFTPISFANNPLLCGPGTTKPCPGDPPFSPPPP
YNPPTPPTQSAGASSTGAIAGGVAAGAALVFAVPAIAFAMWRRRKPEEHFFDVPAEEDPEVHLGQLKKFSLRELQ
VASDNFNNKNILGRGGFGKVYKGRLADGTLVAVKRLKEERTPGGELQFQTEVEMISMAVHRNLLRLRGFCMTP
TERLLVYPYMANGSVASRLRERQPSEPPLDWDTRRRIALGSARGLSYLHDHCDPKIIHRDVKAANILLDEDFEAV
VGDFGLAKLMDYKDTHVTTAVRGTIGHIAPEYLSTGKSSEKTDVFGYGITLLELITGQRAFDLARLANDDDVML
LDWVKGLLKEKKVEMLVDPDLQSNYEEAEVESLIQVALLCTQGSPVERPKMSEVVRMLEGDGLAERWEEWQK
VEVVRQEAELAPLRNDWIVDSTYNLRAVELSGPR*

>*A._americanus*_BAK1
MGMERGISASVFLWLILVFHPLARVLANMEGDALHNLRTNLNDPNNVLQSWDPTLVNPCTWFHVTCNND
NSVVRVDLGNAALSGTLVPQLGQLKNLQYLELYSNNISGSIPSDLGNLTNLVSLDLYLNNFSGPIPDTLGNL
SKLRFLRLNNNSLAGQIPVSLTNVSALQVLDLSNNHLSGPVPSNGSFSLFTPISFNNNPQLCGPGTSHPCPGEP
PFSPPPPFVPASPSPTQGGNSASSTGAIAGGVAAGAALLFAAPAIGFAWWRRRKPQEHFFDVPAEEDPEVHL
GQLKRFSLRELQVATDSFSNKNILGRGGFGKVYKGRLADGSLVAVKRLKEERTPGGELQFQTEVEMISMA
VHRNLLRLRGFCMTPTERLLVYPYMANGSVASCLRERPPNEPPLDWPTRKRIALGSARGLSYLHDHCDPKII
HRDVKAANILLDEEFEAVVGDFGLAKLMDYKDTHVTTAVRGTIGHIAPEYLSTGKSSEKTDVFGYGIMLLE
LITGQRAFDLARLANDDDVMLLDWVKGLLKEKKLDMLVDPDLQNNYVEIEVEALIQVALLCTQGSPVDRP
KMSEVVRMLEGDGLAERWEEWQKVEVVRQEVELVPHRNSEWILDSTDNLHAVELSGPR*

>*D._alata*_BAK1
MLMEREGMTAWFLWLILVFQPLARVLANMEGDALHQLRTNLNDPNSVLQSWDPTLVNPCTWFHVTCNN
DNSVIRVDLGNAALSGSLVPQLGQLKNLQYLELYSNNISGTIPSELGNLTNLVSLDLYLNNFTGVIPDTLGN
LSKLRFLRLNNNTLTGQIPTSLTNINALQVLDLSNNNLSGEVPSSGSFSLFTPISFGNNPFLCGPGTTKPCPGA
PPFSPPPPFLAPGPPSSQGSSPSSTGAIAGGVAAGAALLFAAPAIGFAWWRRRKPQELFFDVPAEEDPEVHLG
QLKRFSLRELQVATDTFSNKNILGRGGFGKVYKGRLADGSLVAVKRLKEERTPGGELQFQTEVEMISMAV
HRNLLRLRGFCMTPTERLLVYPYMANGSVASCLRERPPSEPPLDWSTRRRIALGSARGLSYLHDHCDPKIIH
RDVKAANILLDEEFEAVVGDFGLAKLMDYKDTHVTTAVRGTIGHIAPEYLSTGKSSEKTDVFGYGIMLLEL
ITGQRAFDLARLANDDDVMLLDWVKGLLKEKRLEMLVDPDLQDNYIEAEVESLIQVALLCTQGSPMERPK
MSEVVRMLEGDGLAERWEEWQKVEVVRHEVEMAPHRSSEWILDSTDNLHAVELSGPR*

>*A._officinalis*_BAK1
MLAVMEMDYLAPSWFLFLILVAHPLARVLANMEGDALHSLQANLKDPNNVLQSWDPTLVNPCTWFHVT
CNSDNSVVRVDLGNANLSGQLVPQLGQLKNLQYLELYSNNIGGSIPTDLGNLKNLVSLDLYLNNFTGGIPD
SLGNLSKLRFLRLNNNSLAGSIPKSLTSINTLQVLDLSNNNLSGEVPSTGSFSLFTPISFANNPLLCGPGTAKP
CPGAPPFSPPPPFKPPIPQSPQGSSAPSTGTIAGGVAAGAALLFAAPAIGFAWWRRRKPQEYFFDVPAEEDPE
VHLGQLKRFSLRELQVATDGFSNKNILGKGGFGKVYKGRLVDGSLVAVKRLKEERTAGGELQFQTEVEMI
SMAVHRNLLRLRGFCMTPTERLLVYPYMANGSVASCLRERPPAELPLEWPIRRRIALGSARGLSYLHDHCD
PRIIHRDVKAANILLDEEFEAVVGDFGLAKLMDYKDTHVYTAVRGTIGHIAPEYLSTGKSSEKTDVFGYGI
MLLELITGQRAFDLGRLAKDDDVMLLDWVKGLLKERKLETLIDPELQSNYIEAEVESLIQIALLCTQGSPME
RPKMSEVVRMLEGDGLAERWEEWQKVEVVRQEVEMAPHCNSEWILDSTDNLHAVELSGPR*

>*C._nucifera*_BAK1
MATVERDAIVLWFLCLILVFYPVARVLANMEGDALHILRTNLNDPNNVLQSWDPTLVNPCTWFHVTCNN
DNSVIRVDLGNAALSGTLVPQLGQLKNLQYLELYSNNISGAIPSELGNLTELVSLDLYLNNFTGVIPDSLGD
LSKLRFFRLNNNNLSGHIPQSLTKISTLQVLDLSNNNLSGEVPSTGSFSLFTPISFNNNPLLCGPGTTKPCPGSP
PFSPPPPYNPTPASSPGNRASSTGAIAGGVAAGAALLFAAPAIGFAWWRRRKPQEHFFDVPAEEDPEVHLGQ
LKRFSLRELQVATDGFNNKNILGRGGFGKVYKGRLADGSLVAVKRLKEERTPGGELQFQTEVEMISMAVH
RNLLRLRGFCMTPTERLLVYPYMANGSVASCLRERPPSEPPLSWAARRRIAIGSARGLSYLHDHCDPKIIHR
DVKAANILLDEEYEAVVGDFGLAKLMDYKDTHVTTAVRGTIGHIAPEYLSTGKSSEKTDVFGYGIMLLELI
TGQRAFDLARLANDDDVMLLDWVKGLLKERRLEMLVDPDLQNNYVEAEVESLIQVALLCTQGSPMDRPK
MSEVVRMLEGDGLAEKWEEWQKVEVVRQDVEMAPHRKSEWIIDSTDNLNADELSGPR*

>*Z._officinalis*_BAK1
MARVVWELMLPRFFWFILTFDTFSTVLSNMEGDALHYLKTNLNDPNNVLQSWDPTLVNPCTWFHVTCNN
DNSVIRVDLGNAQLSGTLVPQLGQLKNLQYLELYSNNISGTIPSDLGNLTNLVSLDLYLNNFTGEIPDSLGK
LTKLRFLRLNNNSLSGPIPKSLTNITALQVLDLSNNNLSGEVPSTGSFSLFTPISFANNPLLCGPGTTKACPGA
PPLSPPPPFVPPMPPSSQGSSASSTGAIAGGVAAGAALLFAVPAILFAWWRRRKPPEHFFDVPAEEDPEVHLG
QLKRFSLRELQVATDNFSNKNILGRGGFGKVYKGRLADGSLVAVKRLKEERTPGGELQFQTEVEMISLAV
HRNLLRLRGFCMTPTERLLVYPYMANGSVASRLRERPPSEPPLEWATRRKIALGSARGLSYLHDHCDPKIIH
RDVKAANILLDEDFEAIVGDFGLAKLMDYKDTHVTTAVRGTIGHIAPEYLSTGKSSEKTDVFGYGIMLLELI
TGQRAFDLARLANDDDVMLLDWVKGLLKEKKLEMLVDPDLQNDYVEAEVESLIQVALLCTQGSPIERPK
MSEVVRMLEGDGLAERWEEWLKVEVGRLEEMAPRLLTNEWILDSTDNLRPVELSGPR*

S. cereale BAK1	100.00	85.60	82.78	84.78	85.58	84.48	85.28
Z. officinalis BAK1	85.60	100.00	84.87	86.88	86.26	86.60	88.34
A. officinalis BAK1	82.78	84.87	100.00	86.10	86.76	87.56	88.66
***S. polyrhiza* BAK1**	**84.78**	**86.88**	**86.10**	**100.00**	**88.02**	**90.58**	**88.96**
C. nucifera BAK1	85.58	86.26	86.76	88.02	100.00	89.30	90.40
A. americanus BAK1	84.48	86.60	87.56	90.58	89.30	100.00	91.23
D. alata BAK1	85.28	88.34	88.66	88.96	90.40	91.23	100.00

BKI1

>*A._thaliana*_BKI1
METNLQQVKNSSQTFSEKQNPKQEASPSPISSTCSSPSHDFSFTISLQPLSSSSKHISPTLRSPSKTTSSYQQTDPFAVDLSPA
DEIFFHGHLLPLHLLSHLPVSPRTSTGSYNDGFTLPVKDILPDQPTNNNNNTENAITNISTEAKDDNTEDKAEGEIRVKTK
PIKSFSLFGLSKWRKGFESNEREQEQQQQKIKKPMSLDLSHAVKKYIRMLFQKRGNGTQFWNRRQTSSYSFSSSLMGPN
GNSKTMINGSYNKRDLIRGRRGELFSAPASMRTSPTNSGHLRVSTAGLSSSSGSTSSSSSDSTMEELQAAIQAAIAHCKNS
SAVDRDDKVKDS*

>S._polyrhiza_BKI1
None!

>*S._cereale*_BKI1
MDAPRPRSPPPLFMPTTPPSPPPPPPPLSSSPSPDFSFSFSPFPPSPPPCHVHLRVLPLPAAADMSRTPLGRVGSDISHNNYA
KANHRQATSHSSCSSDDRDRAKTRASPFFPGPGGPWRSGEGRDDTAGKAEEDKKKVKGKRGPLEVGQRVKKYMASL
VEQLLASFSRHGERDRRGQRRRPHTFSVSGPGAAATMERERWRQRRGQLSSAPASLRASPVNSGHLSVGGSLVKVSTSS
EESTMEELQSAIQAAIAHCKNSIAVAKQ*

>*A._americanus*_BKI1
MATQKDSKMVVVGGEEKKEHKTKGHPSPPPPSPPPPQRNLIASTTSPPTSSSSSPSHEFSFTISLHPSIDDPNQYNNTTKSP
PPFTAIDLAPADDIFFHGHLLPLRILSRQVLIPPTTTTTTTTSSSSSSAVSFDPPIEEPKPPNTTTTATNDTKPKKPNKPIRNL
SNLFGAEFSRVVKKYVSASLLFLRGSSTREKAEAVRRRPCTFSGKDSDSVLKERRERWKGRRGGQFSAPASMRTSPTNS
GLLMATAGGATFSSSDESTMEELNNAIQAAVAHCKNSIAMKEEKCKC*

>*D._alata*_BKI1
MDSEEHHRLSEKEATTTTTTTSPSHEFSFTISLHPSPNSNPTSIKYSKNNPTFAIDLAPADDIFFHGHLLPLHLLSSTSISPRP
SDFSIENLNLPLDSTDGNQSQKYETYNNNNMLINEANETKEKFKPKNFSSSLFGFGKWLPKAGGGGEKHESGKTKKKK
MLDLSRVFKRYVNAIELLFTFRSDKEKQRLPPRPYSFSGNISVKDKEAWRKRRGELSAPASMRTSPTNSGLLVATSSVFS
SSDESTMEELQNAIQAAISHCKNSIAVSEEKCIC*

>*A._officinalis*_BKI1
MNEFEISIDVLGRRPGYLNGYRIYLRRSLSTRSIAKSAEQDAEELGFDYLGSREQLVMRVLRIVKKGVAQLTGSNLSPSL
TQISLAPADDIFFHGHLLPLHLRSHPRPSTDISVDDITIIPDGSHPINLNVNNKKNKPISLSSLLKLTKWLEDKKKHFLKKFF
SKEEKHKRDLQRRPYSFSGNSNRKEKWISRIGEFSAPASMRTSPTNSGLLPATPPGYSSSNESSMEELQNAIQAAIAHCKN
SSATKERQVEV*

>*C._nucifera*_BKI1
MDSHKPQRGEREANQGSKEDGEALPPPLPPSSNSSPSHDFSFAISVLPSSISSPNPIKCNKTTAPSNAVDMAPADDIFFHGH
LLPLHFVSYSSISSRPSDISIEDLSLPLEHLGSDGSLKYQNNYRHNNSETGETRERTKFKSLSSFFGLVKWRKASNTGEKEE
NKKKEKKGIDVMSRFLKKYVTMPELFFFFKGKREKRDLRRRPYSISGYSNPKERVGWRRRRGEFSAPASMRTSPTNSGL
LSAYSDVSTMEELQSAIQAAIAHCKNSTAPKGEK*

>*Z._officinalis*_BKI1
MEIEDNVTSPPRPPHPLPPSAITSPSHDFSFTISFQTLSSPATLRGSAAKSPSASSGAFDLAPADDIFLHGHLLPLRLLSHPGS
PPRDSDISFENFRFPLGHTDSDRNLNFFDSGKAGAKEESRATKPSSSALAAFFGLGKLRRRNEKEGAEAAATTTTKRKRK
GIDVIRLLRKYANLVELLFFSRTGERERERRRHEDDDLRRRPCSFSGHSNRNSKAEAAAQGEWRRRKKGQMSAPASMR
TSPSNSGLLSASSMTISSTSTSDSSTMEELQSAIQAAIAHCKNSIAVAAKQRAGDT*

S. cereale BKI1	100.00	32.07	38.83	40.85	36.89	43.58
A. officinalis BKI1	32.07	100.00	43.52	44.34	45.12	49.04
A. americanus BKI1	38.83	43.52	100.00	40.41	49.58	45.38
Z. officinalis BKI1	40.85	44.34	40.41	100.00	43.94	48.85
D. alata BKI1	36.89	45.12	49.58	43.94	100.00	53.61
C. nucifera BKI1	43.58	49.04	45.38	48.85	53.61	100.00

BSU1

>*A._thaliana*_BSU1
MAPDQSYQYPSPSYESIQTFYDTDEDWPGPRCGHTLTAVFVNNSHQLILFGGSTTAVANHNSSLPEISLDGV
TNSVHSFDVLTRKWTRLNPIGDVPSPRACHAAALYGTLILIQGGIGPSGPSDGDVYMLDMTNNKWIKFLVG
GETPSPRYGHVMDIAAQRWLVIFSGNNGNEILDDTWALDTRGPFSWDRLNPSGNQPSGRMYASGSSREDGI
FLLCGGIDHSGVTLGDTYGLKMDSDNVWTPVPAVAPSPRYQHTAVFGGSKLHVIGGILNRARLIDGEAVV
AVLDTETGEWVDTNQPETSASGANRQNQYQLMRRCHHAAASFGSHLYVHGGIREDVLLDDLLVAETSQS
SSPEPEEDNPDNYMLLDDYLMDEPKPLSSEPEASSFIMRSTSEIAMDRLAEAHNLPTIENAFYDSAIEGYVPL
QHGAETVGNRGGLVRTASLDQSTQDLHKKVISTLLRPKTWTPPANRDFFLSYLEVKHLCDEVEKIFMNEPT
LLQLKVPIKVFGDIHGQYGDLMRLFHEYGHPSVEGDITHIDYLFLGDYVDRGQHSLEIIMLLFALKIEYPKNI
HLIRGNHESLAMNRIYGFLTECEERMGESYGFEAWLKINQVFDYLPLAALLEKKVLCVHGGIGRAVTIEEIE
NIERPAFPDTGSMVLKDILWSDPTMNDTVLGIVDNARGEGVVSFGPDIVKAFLERNGLEMILRAHECVIDGF
ERFADGRLITVFSATNYCGTAQNAGAILVIGRDMVIYPKLIHPHPPPISSSEEDYT
DKAWMQELNIEMPPTPARGESSE*

>*S._polyrhiza*_BSU1
**MGSTPWLQNAPPYRVMETSWETEDDAPGPRCAHTLTAVAASKNHGPRLILFGGATAIEGGPSSSVPG
IRLAGVTNSVHAYCVVTRKWTRLRPAGDPPSPRAAHAAAAVGTMVVFQGGIGPAGHSTDDLYVLDL
TNDRCKWHRVVVQGAGPGPRYGHVMDLVGQRFLVTVSGNDGKRALSDAWALDTARKPYKWQKL
NPEGDKPTARMYATASVRSDGMLLLCGGRDSSNTPLADAYGLSLHTNGRWEWNLAPGASPSPRYQH
SSVFVDARLHVTGGALRGGRSVEGDAAVAVLDTASWIWLDRKGPVSSPRTSKASGSDPSVELLRRCR
HASACVGARIYVYGGLKGAAELPTSSDSQQAASSAYSRLDTESIEILIEASAAEAEAGSAAWLAAKAAS
EVSMEDSSSELNRYTDQGSDNLQSQEGSFDDNSLDVLEPDVRLHPRAVVVAKETVGNLGGLIRQLSL
DQFENESRRVYPSNSDQSYNGKKNLSRQRSPQGLHKKIISLLLRPRHWKPPTNRRFFLDTYEIGELCY
AAEQIFIQEPTVLQLKAPVKVFGDLHGQFGDLIRLFDEYGFPSTAGDITYIDYLFLGDYVDRGQHSLE
TVALLLALKIEYPENIHLIRGNHEAADINALFGFRLECIERMGENDGIWAWTRFNQMFNFLPLAALIE
KKIICMHGGIGRSINYVEQIEKLERPITMDAGSIVLMDLLWSDPTENDTIEGLRPNARGPGLVTFGPD
RVTDFCKRNKLQLIIRAHECVMDGFERFAQGQLITVFSATNYCGSANNAGAILVIGRGLVVVPKLIHP
LPPPLQSPESSPDRVTDSWMQELNIQRPPTPTRGRPQSAGDRNSLAY***

>*S._cereale*_BSU1
MGTAGKGAWVVPAPAYKEVEGWEGSGDDSPGYRCGHSLTVIAPTKGHGPRLILFGGATAIEAGATSGLPG
IRLAGVTNTVHSYDVDKRRWTRLHPAGDPPSPRAAHSAAAVGTMVVFQGGIGPAGHSTDDLYVLDLTND
KFKWHRVVVQGAGPGPRYGHCMDLVAQRYLVSVSGNDGKRVLSDAWALDTAQKPYKWQKLNPDGDRP
SARMYGTASARSDGMLLLCGGRDASGTPLSDAYGLLMHTNGQWEWTLAPGISPSPRYQHAAVFVGARLH
VTGGVLRGGRAIEGEGAIAVLDTAAGVWLDRNGIVTSRTLKSSNEHDASSDLLRRCRHAAASVGSQIYIYG
GLRGDILLDDFLIAENAPFQSETDRVPRSENQNRNHNFNSDSPPFEQYTNNNHETAPGFSTDKKSIDMLTEA
SAAEAEAVSAVWRAAKEASHASSEDSLSDGIGSESPLSETSPMADDLDDGGSMEPDVKLHSRAVVVAKEA
VGDLGCLVRQLSLDQFENESRRMHPANNDQSYSSKKALNRQRSPQGLHKKVISLLLRPRNWNAPADRTFF
LDSYEVGELCYAAEQIFMQEPTVLQLKAPVKVFGDLHGQFGDLMRLFDEYGYPSTAGDITYIDYLFLGDYV
DRGQHSLETITLLLALKVEYPEHVHLIRGNHEAADINALFGFRLECIERMGESDGIWAWTRFNQLFNYLPLA
AMIEKKIICMHGGIGRSINSVEQIEKIERPITMDVGSIVLMDLLWSDPTENDSVEGLRPNARGPGLVTFGPDR
VAEFCKRNKLQLIIRAHECVMDGFERFAHGQLITLFSATNYCGTANNAGAILVVGRGLVIVPKLIHPLPPPIN
SPESSPERAMDATWMQELNIQRPPTPTRGRPHSAGDRNSLAYI*

>*A._americanus*_BSU1
MGSKPWMVPPPTYRALVTSWDTEDDAPGPRCSHTLTAIAPTKSHGPRLILFGGATAIEGGASAGIRLAGVT
NSVHSYDVLTRKWIRIKPAGDPPSPRAAHAAAAVGTMVVFQGGIGPAGHSTDDLYVLDLTNDKFKWHRL
VVQGAGPGPRYGHAMDLVAQRYLVTVSGNDGKRVLYDSWCLDTAQKPYKWQKLNPEGDRPSARMYAT
ASARSDGLLVLCGGRDSSGTPLSDAYGLLMHTNGRWEWNLAPGVSPSPRYQHAAVFVGTRLHVTGGALR
GGRTLEGDSVTAVLDTAPGLWLDRNGVITSSRTNKTVTEFDISLELLRRGRHASASVGTRIYIFGGLRGDILL
NDLLIAEDPPFQSEVSSPIPVSEKSPNVSSPKHTQSGTSPESLNGTSSLDGSLDKRPIENHLEASAAEYEAVSQ
FWKNANAVSPIPLDEMPNDTSKSAAPETSSDESHAVSVEPDVRLHSRAVVVAKETVGNLGGLVRQLSLDQ
FENESRRMIPSNGDQAYPTKKFFNRQKSPQGLHKKIISSLLMPRSWKAPTNRRFFLDSYEVGELCYAAEQIF
MQEPTVLQLKAPIKVFGDLHGQFGDLMRLFDEYGFPSTAGDITYIDYLFLGDYVDRGQHSLETITLLLALKI
EYPENVHLIRGNHEAADINALFGFRLECIERMGENDGIWAWTRFNQLFNTLPLAALIEKKIICMHGGIGRSIN
SVEQIEKLERPITMDVGSGILMDLLWSDPTENDSIEGLRPNARGPGLVTFGPDRVSDFCKRNKLQLIIRAHEC
VMDGFERFAHGQLITLFSATNYCGTANNAGAILVVGRGLVIVPKLIHPLPPPLQSPETSPEHALSDETWMQE
LNIQRPPTPTRGRPQPLDRSSLAY*

>*D._alata*_BSU1
MVVFQGGIGPAGHSTDDLYVLDLTNDKYKWHRLVVQGAGPGPRYGHAMDLVAQRFLVTVSGNDGKRVL
SDAWALDTAQKPYRWQKLNPEGDKPSARMYATASARSDGMLLLCGGRDHSGVPLSDAYGLLMHTNGQ
WEWNLAPGVSPSPRYQHAAVFVGARLHVTGGVLKGGRAVEGDGAIAVLDTAAGVWLDRNGIVTSTRVN
KSAADLDPLELLRRCRHGSASVGTQIFIFGGLKGDVLLDDLLVAENSPFPSEITSSMYSIPSVTNHDKNNSSS
PVRSGLNPASDGLSMDQKSIDTLVAASAAEAEAVSAAWLAAKEASSVSSDSDLSDRNGPLPDAEDKSQVM
DASSDDGLASPLQPDVRLHPRAVVIAKEAVGNLGGLVRQLSLDHFENESRRMYPSNADQAYSNKRTLNRQ
KSPLGLHKKIISLLLRPRNWKAPANRNFFLDSYEVGELCYAAEQIFMQEPTVLQLKAPVKVFGDLHGQFGD
LMRLFDEYGFPSTAGDITYIDYLFLGDYVDRGQHSLETITLLLALKIEYPENIHLIRGNHEAADINALFGFRLE
CIERMGENDGIWAWTRFNQLFNTLPLAALIEKKIICMHGGIGRSINSVEQIEKLERPITMDVGSIVLMDLLWS
DPTENDSVEGLRPNARGPGLVTFGPDRVSDFCKRNKLQLIIRAHECVMDGFERFAHGQLITVFSATNYCGT
ANNAGAILVIGRGLVVVPKLIHPLPPPLNSPESSPEHVLEDTWMQELNIQRPPTPTRGRPQSASDRSSLAYI*

>*A._officinalis*_BSU1
MGSKPWMVPAPRYRRDGVTVGLRRRLARPALAHHTRDGGGPPPRTTATRLILFGGATAIEGGPSGIRLDGV
TNSVHSYDVETKKWTRIRPAGEPPSLRAAHAAAAVGTMVVFQGGIGPAGHSTDDLYVLDLTNDKYKWHR
LVVQGDGPGPRYGHAMDLVAQRYLVSVSGNDGKRVLSDAWALDTAQKPYRWQKLNPVGDKPSARMYA
TASARSDGMLLLCGGRDISGTPLADAYGLLMHTNGQWEWTLAPGVSPSPRYQHAAVFVGARLHVTGGVL
RGGRSIEGEGAVAVLDTAAGVWLDKNGIVTTSSRSNKSSANDRDASFELLRRCRHAVASVGSQIYVHGGL
RGDILLDDFLVAENAPFQSEIDSSMYSINSTPSATNHKLEQPSLDSIPERGSSGGSSLDQKSIDKLVQASTAEA
EAASAVWQAAREASEKGLSDDNEASPQAYEKSETSDINDGLERDVRLHPRAVVVAKEAVGNLGGLVRQL
SLEQFENEGRRVNPANGDQVYPPKKFLNRQKSPQGLHKKIISFLLRPRNWKSPANRSFFLDAYEVGELCYA
AEQIFMQEPTVLQLKAPVKVFGDLHGQFGDLMRLFDEYGFPSTAGDITYIDYLFLGDYVDRGQHSLETITLL
LALKIEYPENVHLIRGNHEAADINALFGFRLECIERMGESDGIWVWNRFNQLFNYLPLAALIEKKIICMHGGI
GRSINSVEQIEKLERPITMDVGSIILMDLLWSDPTENDSIEGLRPNARGPGLVTFGPDRVTDFCKRNKLQLIIR
AHECVMDGFERFAQGQLITLFSATNYCGTANNAGAILVVGRGLVIVPKLIHPLPPPLHSPESSPERVSDDTW
MQELNTQRPPTPTRGRPQSASDRNSLAY*

>*C._nucifera*_BSU1
MGSRTRPVPAPSYRPVEASWESAGDSPGPRCAHTLTAVPATGSHGPRLILFGGTTAIEGSPSGPGIRLAGAT
NSVHCFDIQTRKWTRIQPAGEPPSPRAAHVAAAVGTMVVFQGGIGPAGHSSDDLYVLDVTNDKFKWHRV
VVQGPGPGPRYGHVMDLVAHRYLVIVSGYDGKRCLSDEWALDTAQKPYRWHRLEPEGDKPSARVYATA
SSRSDGLLLLCGGRDSSGMPQSDAYGLLMHTSGRWEWTRAPGVSPSPRYQHSVAFIGARLHVTGGALRGG
RTVEGEGAIVVLDTAAGVWLDRNGIVTSSRMRKSSNDSDTSLGHLNRCRHASASTGFQIFVYGGLRGDML
LDDLVIAEDTYFQSAFSSISMENQNLNQYVDSEYPPMLSSSNKRQEAACYPSLSMDQKSAEKLAEASAAEA
EAVTAIWQAVKGASAVHTEGALSENNSGSKVNGSSDCSSTDGLEPDVHLHPRAVVVAQEAVGDLAGLVR
QLSLEHFENEGRRMYPSNSDPVYPTKFLNRQKSPQGLHKKIISLLLRPRNWKAPVNRSFFLDYCDVDELCH
AAEKIFKQEPTVLQLKAPVKIFGDLHGQFGDLMRLFDEYGFPSTAGDITYIDYLFLGDYVDRGQHSLETISL
LLALKIEYPGSIHLIRGNHEAADINALFGFRMECIERMGESDGIWAWTRFNQLFNFLPLAALIEKKIICMHGG
IGRSINSVEQIEKLERPITMDAGSIILMDLLWSDPTENDSIEGLRPNARGPGLVAFGPDRVADFC
TKNKLQLIIRAHECVMDGFERFAQGQLITVFSATNYCGTANNAGAILVVGRGLVVVPKLIHPLPPPPQSPESS
PEHVLEDTWMQELNIQRPPTPTRGRPQSANDRNSLAYI*

>*Z._officinalis*_BSU1
MGSRPWLNPAPSYHVLESSWETDDDAPGPRCGHSLTAVAATKSHGPRLILFGGATAIEGGASSAAPGIRLA
GVTNSLHSYDVKTRKWTRLHPAGEPPSQRAAHAAAAVGTMVVFQGGIGPAGHSTDDLYVLDLTNDKFK
WHRVVVQGPGPGPRYGHAMDLVAQRYLVTVSGNDGQRVLSDTWALDTAQKPYRWQKLNPEGDRPTAR
MYATASARSDGMLLLCGGRDSSGMPLSDAYGLLMHTNGKWEWTLAPGVSPSPRYQHAAVFVGARLHVT
GGALKGGRNVEGEASIAVLDTAAGVWLDRNGVVTSTRTYKSPTDNDGSLELLRRCRHALASVGTQVYVF
GGLKGDVLLDDFLVAENSTFQSEVSSSMSNSGSVPSVNYKPNQNVNLDSQFAEQPLNKKQDSASSASVDQ
KSIEMLTQASAAEAEAVSAVWRAARENISDDGDSEFPIIDETSGEDDLSDVPDSLEPGVRLHPRAVVVAKET
VGNLGGLVRQLSLDQFENESRRMYPTNVEQTYPTKKFLNRQKSPEGLHKKVISHLLRPRNWKPPANRRFFL
DSYEVGELCHAAEQIFRQEPTVLQLKAPVKVFGDLHGQFGDLMRLFDEYGSPSTAGDITYIDYLFLGDYVD
RGQHSLETITLLLALKIENPENIHLIRGNHEAADINALFGFRLECIERMGESDGIWAWTRFNQLFNYLPLAAL
IEKKIICMHGGIGRSINSVDQIEKLERPINMDVGSIVLMDLLWSDPTENDSIEGLRPNARGPGLVTFGPD
RVTEFCKRNKLQLIIRAHECVMDGFERFAQGQLITLFSATNYCGTANNAGAILVVGRGLVIVPKLIHPLPPPL
QSPESSPEHVLEDTWMQELNIQRPPTPTRGRPQSANDRSSLAYI*

C. nucifera BSU1	100.00	77.14	75.35	77.70	75.09	79.18	76.16
***S. polyrhiza* BSU1**	**77.14**	**100.00**	**79.02**	**79.76**	**76.09**	**80.87**	**77.42**
A. americanus BSU1	75.35	79.02	100.00	80.53	77.41	81.05	77.80
Z. officinalis BSU1	77.70	79.76	80.53	100.00	80.00	83.14	79.95
S. cereale BSU1	75.09	76.09	77.41	80.00	100.00	81.74	79.33
D. alata BSU1	79.18	80.87	81.05	83.14	81.74	100.00	83.68
A. officinalis BSU1	76.16	77.42	77.80	79.95	79.33	83.68	100.00

BIN2

>*A._thaliana*_BIN2
MADDKEMPAAVVDGHDQVTGHIISTTIGGKNGEPKQTISYMAERVVGTGSFGIVFQAKCLETGETVAIKKV
LQDRRYKNRELQLMRVMDHPNVVCLKHCFFSTTSKDELFLNLVMEYVPESLYRVLKHYSSANQRMPLVY
VKLYMYQIFRGLAYIHNVAGVCHRDLKPQNLLVDPLTHQVKICDFGSAKQLVKGEANISYICSRFYRAPELI
FGATEYTTSIDIWSAGCVLAELLLGQPLFPGENAVDQLVEIIKVLGTPTREEIRCMNPHYTDFRFPQIKAHPW
HKIFHKRMPPEAIDFASRLLQYSPSLRCTALEACAHPFFDELREPNARLPNGRPFPPLFNFKQEVAGSSPELV
NKLIPDHIKRQLGLSFLNQSGT*

>*S._polyrhiza*_BIN2
**MTQQHESHQLRQQQYLQNEALVKTLPPRPEMGDEKMTEASVVEGNNPRTGHIISTTIGGKNGEPKQ
TISYMAERVVGTGSFGIVFQAKCLETGETVAIKKVLQDKRYKNRELQLMRSMDHPNVISLKHCFFST
TSRDDLFLNLVMEYVPETVYRVLKHYSSVNQRMPLIYVKLYIYQVFKGLAYIHNVQGVCHRDVKPQ
NLLVDPLTHQVKLCDFGSAKVLVKGEANISYICSRYYRAPELIFGAAEYTTSIDVWSAGCVLAELLLG
QPLFPGESAVDQLVEIIKVLGTPTREEIRCMNPSYTEFRFPQIKAHPWHKVFHKRMPPEAIDLTSRLL
QYSPSLRCAALEACAHPFFDELREPAARLPNGRALPPLFSFKQELVGVSPDLVNKLIPEHARRQGGLA
TT***

>*S._cereale*_BIN2
MEAPPGPEPMVLDAPPPLAAAVPAHAATEKARTEGGDPVTGHIISTTIGGKNGEPKRTISYMAERVVGTGSF
GIVFQAKCLETGETVAIKKVLQDRRYKNRELQLMRSMDHPNVVSLKHCFFSTTSRDELFLNLVMEYVPETL
YRVLKHYSNANQRMPLIYVKLYMYQLFRGLAYVHTVPGVCHRDVKPQNVLVDPLTHQVKICDFGSAKVL
VPGEPNIAYICSRYYRAPELIFGATEYTTSIDIWSAGCVLAELLLGQPLFPGETAVDQLVEIIKVLGTPTREEIR
CMNPNYTEFRFPQIKAHPWHKIFHKRMPAEAIDLASRLLQYSPNLRCTALDACAHSFFDELREPNARLPNG
RPFPPLFNFKPELANASPELINRLVPEHVRRQNGLNFAHAGS*

>*A._americanus*_BIN2
MASLPLGPPPLQGNEGLVKVPSVRRPEMGDDKETSVMEGNGPVTGHIISTTIGGKNGEPKRTISYMAERVV
GSGSFGIVFQAKCLETGETVAIKKVLQDRRYKNRELQLMRSMDHPNVVCLKHCFFSTTNRDELFLNLVME
YVPENLYRVLKHYSDVNQRMPLIYVKLYTYQIFRGLAYIHTVPGVCHRDVKPQNLLVDPLTHQVKLCDFG
SAKILVNGEANISYICSRYYRAPELIFGATEYTTSIDIWSAGCVLAELLLGQPLFPGDSAVDQLVEIIKVLGTP
TREEIRCMNPNYTEFRFPQIKAHPWYKIFHKRMPPEAIDLTSRLLQYSPSLRCTALEACAHSFFDELREPNAR
LPNGRSLPPLFNFKQELMGASPDLINRLIPEHVRRQMGLNFVHPAGT*

>*D._alata*_BIN2
MASMPLGPQPPPPDRDVVMTDCRRPEVADDKEASVVDNNDTATGHIISTTIGGKNGEAKRTISYMAERVV
GTGSFGIVFQAKCLETGETVAIKKVLQDKRYKNRELQLMRSMDHPNVISLKHCFFSTTNKDELFLNLVMEY
VPETLYRVLKHYSNVNQRMPLIYVKLYTYQIFRGLAYIHTVPGVCHRDVKPQNLLVDPLTHQVKLCDFGS
AKVLVKGEANISYICSRYYRAPELIFGATEYTTTIDIWSAGCVLAELLLGQPLFPGESAVDQLVEIIKVLGTPT
REEIRCMNPNYTEYRFPQIKAHPWHKVFHKRMPPEAIDLASRLLQYSPSLRCSALEACAHPFFDELREPNAR
LPNGRPLPPLFNFKQELAGASPELINKLIPEHIRRQAGLSFTHPAGT*

>*A._officinalis*_BIN2
MASLPMGPPLLRPHHHQHHQEKNENEILVKNLDPCRPEMAEDKVTSVMEGNDPVTGHIISTTIGGKNGEPK
QTISYMAERIVGTGSFGIVFQAKCLETGETVAIKKVLQDRRYKNRELQLMRSMDHPNVISLKHCFFSTTSRD
ELFLNLVMEYVPETVYRVLKHYSNANQRMPLIYVKLYMYQIFRGLAYIHSVPGVCHRDVKPQNLLVDPLT
HQVKLCDFGSAKVLVKGEANISYICSRYYRAPELIFGATEYTTSIDIWSAGCVLAELLLGQPLFPGESAVDQ
LVEIIKVLGTPTREEIRCMNPNYTEFRFPQIKAHPWHKIFHKRMPPEAIDLASRLLQYSPSLRCTALEACAHSF
FDELREPNARLPTGRPLPPLFNFKQELSGASPELINKLIPEHVRRQPGFNLLIPAGT*

>*C._nucifera*_BIN2
MASLPPLGPHHHPDFQALNLAAAPRRPEMAEDKQASVIEGSDQVTGHIISTTIGGKNGEPKQTISYMAERVV
GTGSFGIVFQAKCLETQETVAIKKVLQDRRYKNRELQLMRAMDHPNVISLKHCFFSTTSRDELFLNLVMEY
VPETLYRVLRHYSNVNQRMPLIYVKLYTYQLFRGLAYIHTVPGVCHRDVKPQNVLVDPLTHQVKLCDFGS
AKVLVKGEANISYICSRYYRAPELIFGATEYATSIDIWSAGCVLAELLLGQTLFPGESAVDQLVEIIKVLGTP
TREEIRCMNPNYTEFRFPQIKAHPWHKIFHKRMPPEAIDLTSRLLQYSPSLRCTALDACAHPFFDELREPNAR
LPNGRALPPLFNFKHELAGASPELINKLIPEHVKRQSGLSFLHPAGT*

>*Z._officinalis*_BIN2
MASLPLGPHQPPPPQPAHDPAAGALLSLAPPHLTEMADNNKQESVVEASESVTGHIISTTIGGKNGEPKQ%0
ATISYMAERVVGTGSFGIVFQAKCLESGETVAIKKVLQDKRYKNRELQLMRFMDHPNVISLKHCFFSTTVR
%0ADELFLNLVMEYVPESLYGVLRHYSSANQRMPLIHVKLYTYQIFRGLAYIHTVSGVCHRDLKPQNVLV
DPL,THQVKICDFGSAKVLVKGEANISYICSRYYRAPELIFGATEYTSSIDVWSAGCVLAELLLGQPLFPGES
A%0AVDQLVQIIKVLGTPTREEIRCMNPNYTEFRFPQIKAHPWHKIFHKRMPPEAIDLISRLLQYSPDFRCSA
L%0AEACAHPFFNELREPNMRLPNGRPLPPLFNFKQELAGASPELINRLIPEHMGRQSGLNFLHSAGT*

Z. officinalis BIN2	100.00	81.41	81.16	85.36	86.35	83.66	84.11
***S. polyrhiza* BIN2**	**81.41**	**100.00**	**81.91**	**87.63**	**86.04**	**86.89**	**86.50**
S. cereale BIN2	81.16	81.91	100.00	84.34	85.10	85.53	84.75
D. alata BIN2	85.36	87.63	84.34	100.00	88.50	88.56	88.37
C. nucifera BIN2	86.35	86.04	85.10	88.50	100.00	88.22	89.41
A. officinalis BIN2	84.11	86.50	84.75	88.37	89.41	89.88	100.00

BZR1/BES1

>*A._thaliana*_BES1
MTSDGATSTSAAAAAAAMATRRKPSWRERENNRRRERRRRAVAAKIYTGLRAQGNYNLPKHCDNNEVL
KALCSEAGWVVEEDGTTYRKGHKPLPGDMAGSSSRATPYSSHNQSPLSSTFDSPILSYQVSPSSSSFPSPSRV
GDPHNISTIFPFLRNGGIPSSLPPLRISNSAPVTPPVSSPTSRNPKPLPTWESFTKQSMSMAAKQSMTSLNYPF
YAVSAPASPTHHRQFHAPATIPECDESDSSTVDSGHWISFQKFAQQQPFSASMVPTSPTFNLVKPAPQQLSP
NTAAIQEIGQSSEFKFENSQVKPWEGERIHDVAMEDLELTLGNGKAHS*

>*S._polyrhiza*_BES1
MTSRVGRLPTWKERENNKRRERRRRAIAAKIFSGLRAYGNYKLPKHCDNNEVLKALCAEAGWIVED
DGTTYRKGCKPAQPSPEMAARGTPANLSPCSSPYQPSPLSSSFPSPAPSYHASPSSSSFPSPSRLENSANP
NIDPSHLLPFLRNIALPPLRISNSAPVTPPLSSPTASRPPKLRKSEWDCAAAFPHPLFAVSAPASPTRKPA
SIPECDESDASTVDSGRWVSFQMAAPPSPTFNLVNPVSRPQAAAPPIAANGISLPERGRGGPEFEFECR
RVKPWEGERIHEVGVDDLELTLGGAKHQVQLNSA*

>*S._cereale*_BES1
MMHGPAGGSGGGHGLGGTRVPTWRERENNRRRERRRRAIAAKIYTGLRAYGNYNLPKHCDNNEVLKALCNEA
GWVVEPDGTTYRRGCKPPPQARTDPMRSTSASPCSSYQPSPRASYNPSPASSSFPSSGSSSHITIGGGNNFVGGVEG
SSLIPWLKNLSSNPSFASSSKLPQLHHLYFNGGSISAPVTPPSSSPTHTPRMKTDWESQCVLPPWAGANYTSLPNST
PPSPGHHVAPDPAWLAGFQISSAGPSSPTYNLVSHNPFGIALASSSRACTPGQSGTCSPVMGDHAPAHHDVQMEM
VDGAPDDFAFGSNSNGNNGSPGLVKAWEGERIHEECASDELELTLGSSKTRGEPPF*

>*A._americanus*_BES1
MTSGGRLPTWKERENNKRRERRRRAIAAKIYTGLRTYGNYKLPKHCDNNEVLKALCIEAGWTVEDDGTT
YRKGCKPPPSEAANMAGTSTNISPCSSQHPSPPSSAFPSPGPSYHASPSSSSFPSPTRFDLSDPTTNPTTNNHQ
NNLNNNANYLIPFLRNLASLPPIRISNSAPVTPPLSSPTSRPSKMSKPDWDHYYYSNAASSAIRQATTLFAAS
APASPTRRHRPGGPATIPECDESDASTIDSGRWVSFGTGPPSSATPASPTYNLVNAIVARPENGGGGLVWGG
GVAENEIGRGGGSEFEFECGKVKAWEGERIHEVGVDDLELTLGGGKHC*

>*D._alata*_BES1
MTSGVGRMPTWKERENNKRRERRRRAIAAKIFSGLRALGNYKLPKHCDNNEVLKALCAEAGWIVEPDGT
TYRKGCKPPPPPPTGAPPLGQSTNISPCSSHHPSPVPSYHASPTSSSFPSPTRISNPAAVNPSYLLPFLHNLSSLP
PLRISNSAPVTPPLSSPTASHPPKIQKPDWDYSAFCHPLFAASAPASPTRARQHFYPATIPECDESDASTVDSG
RWVSFQLSTAPASPTFNLVKPLAPVQDAGIGANANVNASIAEISRGGGTEFEFENRMVKAWEGERIHEVAV
DDLELTLGTRKKHG*

>*A._officinalis*_BES1
MTSGGGRLPTWKERENNKRRERKRRAIAAKIFTGLRTLGNYKLPKHCDNNEVLKALCAEAGWIVEEDGTT
YRKGCKPPPPPPEATTGGGPSTNISQCSSSHHLSPTSSSFPSPVPSYHASPSSSSFPSPNRLDASNPNPNLTPSY
LLPFLRNFSLPPLRISNSAPVTPPLSSPTSSRPQKLKKPDWDYAVHRHPLFNASAPSSPTRGHRHPLFNAPIPE
CDESDVSTVDSGRWVSFQMTAPSSPTFNLVTGPVVGPGGVPEKGRGATEFEFESGTVKPWEGERIHEVGVE
DLELTLGMGNSNAK*

>*C._nucifera*_BES1
MTSGGGRLPTWRERENNKRRERRRRAIAAKIFSGLRTLGNYKLPKHCDNNEVLKALCAEAGWVVEEDGT
TYRKGCKPPPPPPEAAAGGQSTNISPCSSSHHLSPPSSSFPSPVPSYHASPSSSSFPSPTRMENSNPGVNPSYLL
PFLRNLSTLPPLRISNSAPVTPPLSSPTASRPLKIRKPDWDCAAFRHPLFAASAPASPTRGRHHGHPATIPECD
ESDASTVDSGRWVSFQMTAPASPTFNLVKPVAQMPETVVGPGGGGGMSDRGRGGMEFEFENGRVKPWV
GERIHEVGVEDLELTLGVGSSGSK*

>*Z._officinalis*_BES1
MTAGEGRQPTLKERENNKRRERRRRAMAAKIFSGLRSMGNYKLPKHCDNNEVLKALCREAGWIVEEDGT
TYRKGCKPPTPPPEFAGASSGISPCSSPLLSPILSSFPSPVPSYHTSPLTSSFPSPSRNDNVHNPSVNPSSLLPFLQ
NLTSLPPLRISNSAPVTPPPSSPSASHPPKLRNLDNTFCYSLYAISAPSSPIRGHQQGQPMTIPECDESDASTVD
SGQEGAGSHMAVPGSPTFNLVKTFVAAKGTAIGTSLGVPEKGRSMEFEFENRWVKPWEGERIHDVGPDDI
QLTLGVGGTTPKLNVTS*

S. cereale BES1	100.00	52.40	49.08	51.67	48.55	54.32	52.90
A. americanus BES1	52.40	100.00	62.84	72.64	70.00	70.47	74.17
Z. officinalis BES1	49.08	62.84	100.00	68.40	66.22	68.38	69.57
A. officinalis BES1	51.67	72.64	68.40	100.00	74.92	73.45	82.15
***S. polyrhiza* BES1**	**48.55**	**70.00**	**66.22**	**74.92**	**100.00**	**73.81**	**77.23**
D. alata BES1	54.32	70.47	68.38	73.45	73.81	100.00	78.26
C. nucifera BES1	52.90	74.17	69.57	82.15	77.23	78.26	100.00

PP2A

>*A._thaliana*_PP2A
MPPATGDIDRQIEQLMECKALSETEVKMLCEHAKTILVEEYNVQPVKCPVTVCGDIHGQFYDLIELFRIGGS
SPDTNYLFMGDYVDRGYYSVETVSLLVALKVRYRDRLTILRGNHESRQITQVYGFYDECLRKYGNANVW
KHFTDLFDYLPLTALIESQVFCLHGGLSPSLDTLDNIRSLDRIQEVPHEGPMCDLLWSDPDDRCGWGISPRG
AGYTFGQDIATQFNHTNGLSLISRAHQLVMEGFNWCQEKNVVTVFSAPNYCYRCGNMAAILEIGENMDQN
FLQFDPAPRQVEPETTRKTPDYFL*

>*S._polyrhiza*_PP2A
MSVANPVASDSHGNVDEQISQLMQCKPLSELEVRALCDKAKEILMEESNVQPVKSPVTICGDIHGQF
HDLAELFRIGGKCPDTNYLFMGDYVDRGYYSVETVTLLVALKVRYPQRITILRGNHESRQITQVYGF
YDECLRKYGNASVWKTFTDLFDYFPLTALVESEIFCLHGGLSPSIETLDNIRSFDRVQEVPHEGPMCD
LLWSDPDDRCGWGISPRGAGYTFGQDISEQFNHTNNLNLIARAHQLVMDGYNWGHEQKVVTIFSAP
NYCYRCGNMASILEVDDSKGHTFIQFEPAPRRGEPDVTRRTPDYFL*

>*S._cereale*_PP2A
MPPHGDLDRQIAHLRECKHLPEAEVKGLCEQAKAILMEEWNVQPVRCPVTVCGDIHGQFYDLIELFRIGGE
SPDTNYLFMGDYVDRGYYSVETVSLLVALKVRYRDRITILRGNHESRQITQVYGFYDECLRKYGNANVWK
YFTDLFDYLPLTALIENQVFCLHGGLSPSLDTLDNIRALDRIQEVPHEGPMCDLLWSDPDDRCGWGISPRGA
GYTFGQDIAQQFNHTNGLSLISRAHQLVMEGFNWCQDKNVVTVFSAPNYCYRCGNMAAILEIGENMDHN
FLQFDPAPRQIEPDTTRKTPDYFL*

>*A._americanus*_PP2A
MPSHGDLDRQIEHLRECKFLPEAEVKALCEQARAILVEEWNVQPVKCPVTVCGDIHGQFHDLIELFRIGGD
APDTNYLFMGDYVDRGYYSVETVSLLVALKVRYRDRITILRGNHESRQITQVYGFYDECLRKYGNANVW
KYFTDLFDYLPLTALIESQIFCLHGGLSPSLDTLDNIRALDRIQEVPHEGPMCDLLWSDPDDRCGWGISPRGA
GYTFGQDIAAQFNHTNGLSLISRAHQLVMEGFNWCQEKNVVTVFSAPNYCYRCGNMAAILEIGENMDQNF
LQFDPAPRQIEPDTTRKTPDYFL*

>*D._alata*_PP2A
MPASHGDLDRQIERLRECKFLPEAEVKALCEQARAILVEEWNVQPVRCPVTVCGDIHGQFHDLIELFRIGGD
APDTNYLFMGDYVDRGYYSVETVTLLVALKVRYRDRITILRGNHESRQITQVYGFYDECLRKYGNANVW
KFFTDLFDYLPLTALIESQIFCLHGGLSPSLDTLDNIRALDRIQEVPHEGPMCDLLWSDPDDRCGWGISPRGA
GYTFGQDIAQHFNHTNGLSLVARAHQLVMEGFNWCQDKNVVTVFSAPNYCYRCGNMAAIMEIGENMDQ
NFLQFDPAPRQIEPDTTRKTPDYFL*

>*A._officinalis*_PP2A
MPSYSDLDRQIEHLRECKFLPEVEVKALCEQARAILMEEWNVQPVKCPVTVCGDIHGQFHDLIELFKIGGD
APDTNYLFMGDYVDRGYYSVETVSLLVALKVRYRDRITILRGNHESRQITQVYGFYDECLRKYGNANVW
KYFTDLFDYLPLTALIENQVFCLHGGLSPSLDTLDNIRALDRIQEVPHEGPMCDLLWSDPDDRCGWGISPRG
AGYTFGQDIAQQFNHTNGLSLVARAHQLVMEGFNWCQDKNVVTVFSAPNYCYRCGNMAAILEIGENMSQ
NFLQFDPAPRQIEPDTTRKTPDYFL*

>*C._nucifera*_PP2A
MPSHADLDRQIEHLRECKFLPEAEVKVLCEQARAILMEEWNVQPVKCPVTVCGDIHGQFYDLIELFRIGGD
APDTNYLFMGDYVDRGYYSVETVTLLVALKVRYRDRITILRGNHESRQITQVYGFYDECLRKYGNANVW
KYFTDLFDYLPLTALIENQIFCLHGGLSPSLDTLDNIRALDRIQEVPHEGPMCDLLWSDPDDRCGWGISPRG
AGYTFGQDIAQQFNHTNGLSLVARAHQLVMEGYNWCQDRNVVTVFSAPNYCYRCGNMAAILEIGENMD
QNFLQFDPAPRQIEPDTTRRTPDYFL*

>*Z._officinalis*_PP2A
MSKSSERCRRLLHQRPTILHNFSDRDLCLPRAMPPYADLDRQIEHLRECKFLPESEVKALCEQARAILVEEW
NVQPVKCPVTVCGDIHGQFHDLIELFRIGGEAPDTNYLFMGDYVDRGYYSVETVTLLVALKVRYRDRITIL
RGNHESRQITQVYGFYDECLRKYGNANVWKHFTDLFDYLPLTALIENQIFCLHGGLSPSLDTLDNIRDLERI
QEVPHEGPMCDLLWSDPDDRCGWGISPRGAGYTFGQDIAQQFNHRNGLTLVSRAHQLVMEGFNWCQERN
VVTVFSAPNYCYRCGNMAAIMEVGENMEQNFLQFDPAPRQFEPETTRKTPDYFL*

***S. polyrhiza* PP2A**	**100.00**	**75.48**	**77.78**	**78.76**	**77.45**	**79.08**	**77.85**
Z. officinalis PP2A	75.48	100.00	91.18	93.14	93.14	93.46	92.18
S. cereale PP2A	77.78	91.18	100.00	94.44	94.44	95.10	93.14
C. nucifera PP2A	78.76	93.14	94.44	100.00	96.08	95.75	95.10
A. officinalis PP2A	77.45	93.14	94.44	96.08	100.00	96.08	94.77
A. americanus PP2A	79.08	93.46	95.10	95.75	96.08	100.00	96.08
D. alata PP2A	77.85	92.18	93.14	95.10	94.77	96.08	100.00

BSKs

>*A._thaliana*_BSK1
MGCCQSLFSGDNPLGKDGVQPQPLSQNNHGGATTADNGGSGGASGVGGGGGGGGIPSFSEFSFADLKAAT
NNFSSDNIVSESGEKAPNLVYKGRLQNRRWIAVKKFTKMAWPEPKQFAEEAWGVGKLRHNRLANLIGYC
CDGDERLLVAEFMPNDTLAKHLFHWENQTIEWAMRLRVGYYIAEALDYCSTEGRPLYHDLNAYRVLFDE
DGDPRLSCFGLMKNSRDGKSYSTNLAYTPPEYLRNGRVTPESVTYSFGTVLLDLLSGKHIPPSHALDMIRGK
NIILLMDSHLEGKFSTEEATVVVELASQCLQYEPRERPNTKDLVATLAPLQTKSDVPSYVMLGIKKQEEAPS
TPQRPLSPLGEACSRMDLTAIHQILVMTHYRDDEGTNELSFQEWTQQMKDMLDARKRGDQSFREKDFKTA
IDCYSQFIDVGTMVSPTVFGRRSLCYLLCDQPDAALRDAMQAQCVYPDWPTAFYMQSVALAKLNMNTDA
ADMLNEAAQLEEKRQRGGRGS*

>*S._polyrhiza*_BSK
**MSAGQRTEKWAHSPSGGDSGLSAGESQTIEWSMRLRVAYYIAEALDYCNSQGRRLYHDLNAYRVLF
DEDGNPRLSCFGLMKNSRDGKSYSTNLAYTPPEYLRNGRVTPESVIFSFGTVLLDLLSGKHIPPSHALD
MIRGKNILLLMDSHLEGKFSTEEATALVDLASRCLQYDPRERPDTVSLVATLAPLQTRSEVPSQVML
GITKQEEEAPLGPQRPLSPMGDACFRMDLTAIHQILVMTHYRDDEGTNELSFQEWTQQMRDMLDA
RKRGDFAFRDKDFKAAIDCYSQFVDVGTMISPTVYARRSLCHLMCDQADAALRDAMQAQCVYPDW
PTAFYMQAVALAKLNMPSDAADMLNEAAALEEKKQKGGGRGS***

>*S._cereale*_BSK
MGCCGSSLRSWVHAEKPPGPRRPAPPPPPPHRPSFSLKAQKAAPPPPAPRAEEQEEVPALAEFSLAELRAAT
DGFAAGNIVSESGEKAPNLVYRGRLRGAAPRAIAVKKFAKHAWPDPKQFAEEAKGVGKLRHRRMANLIG
YCCDGDERLLVAEFMSNDTLAKHLFHWENQTIEWAMRLRVAYYIAEALGYCSNEERSLYHDLNAYRVLF
DENGDPRLSCFGLMKNSRDGKSYSTNLAYTPPEYLRNGRVTAESVIFSFGTVLLDLLSGKRIPPSHALDMIR
SRNIQALMDSHLEGNYSTEEATTLVNLASQCLQYEPRDRPDIKKLVSILESLQTKSEVPSYVMLGVPKPDEP
SKAPPSPTPQPQHPLSPMGEACSRMDLTAIHQILVSMHYRDDEGSNELSFQEWTQQMRDMLDARKQGDFA
FRDKDFKAAIDCYTQFVDVGTMVSPTVYARRSLCHLMCDQPDAALRDAMQAQCVYPDWPTAFYMQAVA
LSKLDMQSDATDMLNEASQLEEKRQKSSRGP*

>*A._americanus*_BSK
MGCCESCFLTKTGHEKRHRKHLQQVQQPPQQHLLQQRQSFSQNAPEGEAGGVPAFSEYSLADLKAATNNF
SAEFIVSESGEKAPNLVYKGRLQSRRWIAVKKFTKMAWPDPKQFAEEAWGVGKLRHRRLANLIGYCCDG
DERLLVAEYMPNDTLAKHLFHWENQTLEWAMRLRVACYIAEALEYCSSESRPLYHDLNAYRVLFDEDGD
PRLSCFGLMKNSRDGKSYSTNLAYTPPEYLRNGRVTPESVIFSFGTVLLDLLSGKHIPPSHALDMIRGKNILL
LMDSHLEGNFSMDEATALVDLASRCLQYEPRERPNTKNLVDTLAPLQTKSEVPSHVMMNIPKREEAPSTPQ
HPLSPMGEACSRMDLTAIHQILVGTHYRDDEGTNELSFQEWTQQMREMLEARKRGDFAFRDKDFKTAIDC
YTQFIDVGTMVSPTVYARRSLCHLLCDQADAALRDAMQAQCVYPDWPTAFYMQAVALAKLDMHKDAA
DMLNEAAGLEEKKQKNGGRAS*

>*D._alata*_BSK
MGSCCSSELAETQLEKQKHQHANHQQHQPQRASFSLHHAAPLAAVGDAPAAFAEFSLAELKAATGGFSSD
NIVSESGEKAPNLVYKGRLQNRTWIAVKKFTRMAWPDPKQFAEEAWGVGKLRHRRLANLIGYCCDGDER
LLVAEYMPNDTLAKHLFHWENQTIEWAMRLRVAFYIAEALEYCSNEGRPLYHDLNAYRVLFDEDGDPCLS
CFGLMKNSRDGKSYSTNLAYTPPEYLTNGRVTPESVIFSFGTVLLDLLSGKRIPPSHALDMIRGKNILLLMDS
HLEGNFSTEEATALVDLASRCLQYEPRERPSTKDLVATLAPLQPKSEVPSHVMLGISKEEETPAPQHPLSPM
GEACSRMDLTAIHQILVMTHYRDDEGTNELSFQEWTQQMKDMLDARKRGDYAFRDKDFKTAIDCYTQFI
DVGTMVSPTVYARRSLCHLMCDQADAALRDGMQAQCVYPDWPTAFYMQAVALAKLNMHSDAADMLN
EAAGLEEKRQRGGKGT*

>*A._officinalis*_BSK
MPNDTLCKHLFHWENQTIEWAMRLRVPYYIAEALDYCSAEGRPLYHDLNAYRVLFDEDGDPRLSCFGLM
KNSRDGKSYSTNLAYTPPEYLRNGRVTAESVIFSFGTVLLDLLSGKHIPPTHALDMIRGKNILLLMDSHLEG
NFSTEEATSLMDLASRCLQYEPRERPNTKDLVATLEPLQTKLEVPSYVMLGISKHEEAPATPQHPLSPMGEA
CSRMDLTAIHQILVTTHYRDDEGTNELSFQEWTQQMRDMLDARKRGDFAFRDKDFKTAIDCYTQFLDVGT
MISPTVYARRSLCHLMSDQPDAALRDAMQAQCVYPEWPTAFYMQAVALAKLDMHNDAADMLNEAAGL
EKKRQNGMKAS*

>*C._nucifera*_BSK
MGSRVSKLTCCWGSRYKGTVLEAPDVETEETGETYDLPPFQEFSFEQLRLATSGFAVENIVSEHGEKAPN
VVYKGKLDAQRRIAVKRFNRSAWPDPRQFLEEAKAVGQLRNHRLANLLGCCCEGNERLLVAEFMPNDTL
AKHLFHWETQPMKWPMRLRVVLYLAEALEYCTSKGRALYHDLNAYRVLFDDDCNPRLSCFGLMKNSRD
GKSYSTNLAFTPPEYLRTGRVTPESVIYSFGTLLLDVLSGKHIPPSHALDLIRDRNFNMLTDSCLEGQFSNED
GTELVRLASRCLQYEQRERPNVKSLVLALTPLQKETEVASYVLMDMPHGGASSLEALSLSPLGEACSRMD
LTAIHEILEKIGYKDDEGTANELSFQMWTNQMQETLNSKKKGDTAFRHKDFNTAIDCYTQFIDVGTMVSP
TIFARRCLSYLMSDMPQQALNDAMQALVISPTWPTAFYLQAAALLALGMENEAREALKDGSSLETKKDGG
H*

>*Z._officinalis*_BSK
MGSCCSSLPPENRLPGEAAVEAPEEKMKNLPNLHTNHHQPRRSFSPVPDGGGGDEEGVPPFAEFLLPELK
AATNGFSAKNIVSESGDKAPNLVYKGRLQNRRWIAVKKFSRAAWPDPKQFAEEAWGVGKLRHRRLANLI
GYCCDGNERLLVAEYMPNDTLAKHLFHWENQTIEWAMRLRVACFIAEALEYCSNEGRPLYHDLNAYRVL
FDEDGDPRLSCFGHMKNSRDGKSYSTNLAYTPPEYLRNGRVTPESVIFSFGTILLDLLSGKHIPPSHALDMI
RGKNILVLMDSHLEGNFSTEEATSVVDLASQCLQYEPRDRPDIKKLVATLAPLQTKSDVPSHVMLGIQKR
EEAPPTTLHPLSPLGEACSRMDLTAIHQILVMAHYREDQTTNELSFQEWTQQMRDILDARKKGDFAFRDK
DLKTAIECYSQFIDVGTMVSPTVYARRSLCHLMCDQPDAALRDAMQAQYIYPDWHTAFYMQAVALSKLN
MQSDAMDMLQEAAMLEEKRQKGGKVP*

C. nucifera BSK	100.00	60.87	62.11	62.16	63.47	62.50	66.57
***S. polyrhiza* BSK**	**60.87**	**100.00**	**77.75**	**78.23**	**82.57**	**83.29**	**83.66**
S. cereale BSK	62.11	77.75	100.00	75.98	76.97	77.58	84.21
Z. officinalis BSK	62.16	78.23	75.98	100.00	79.13	81.15	84.21
A. americanus BSK	63.47	82.57	76.97	79.13	100.00	84.52	88.64
D. alata BSK	62.50	83.29	77.58	81.15	84.52	100.00	90.00
A. officinalis BSK	66.57	83.66	84.21	84.21	88.64	90.00	100.00

CDGs

>*A._thaliana*_CDG1
MVSCLCFRPSRKTKLKDKSHKRSIRNQTSSSSAQPAGTAKEVDSSSSQTVVQDSSRYRCQIFSYRELAIATNS
FRNESLIGRGGFGTVYKGRLSTGQNIAVKMLDQSGIQGDKEFLVEVLMLSLLHHRNLVHLFGYCAEGDQR
LVVYEYMPLGSVEDHLYDLSEGQEALDWKTRMKIALGAAKGLAFLHNEAQPPVIYRDLKTSNILLDHDYK
PKLSDFGLAKFGPSDDMSHVSTRVMGTHGYCAPEYANTGKLTLKSDIYSFGVVLLELISGRKALMPSSECV
GNQSRYLVHWARPLFLNGRIRQIVDPRLARKGGFSNILLYRGIEVAFLCLAEEANARPSISQVVECLKYIIDH
TIRKERRTRRRLLGGNKDGAGTSRSPDETMMRMLEEEEEYVTSEEAIERRRVIVDDARTWAGMNRRGATP
PTPTP*

>*S._polyrhiza*_CDG
**MGCFPCFGSSEQVKERRGEVKAGGDSKKDPSSTAPSSHHAARVGSEKLKSRSGTETRKETTAPKDGP
THIAAQIFTFRELAAATKNFRQECLLGEGGFGRVYKGRLESTGQVVAVKQLDRNGLQGNREFLVEV
LMLSLLHHPNLVNLIGYCADGDQRLLVYEFMPLGSLEDHLHDIPPDKEPLDWNTRMKIAAGAAKGL
EYLHDKANPPVIYRDFKSSNILLDEGFHPKLSDFGLAKLGPVGDKTHVSTRVMGTYGYCAPEYAMTG
QLTLKSDVYSFGVVLLELITGRKAIDNARAAGEHNLVAWARPLFKDRRKFPKMADPLLQGRYPMRG
LYQALAVAAMCLQEQAATRPLIGDVVTALSYLASQSYDPNAAGAHNSRAGGGPSTPRGREDWKSLS
VGGDSQLAAFSPARNSPDFRKRDLARGVSFGAEVGRGELGGGSGRKTALVEDLEGEESQRGSPMNA
PKARNGPRNANRDADRERAIAEAKVWGENWRDRRHANAQGSFDSTHE***

>*S._cereale*_CDG
MGCFPCFDSGSDGELLYPKQGGGGGGNGTGGRTAAAASSSGVGAREERPMVPPRVEKLPAGAEKARARG
NAGMKELSDLRDANGNVLSAQTFTFRQLTAATRNFREECFIGEGGFGRVYKGRLDGGQVVAIKQLNRDGN
QGNKEFLVEVLMLSLLHHQNLVNLVGYCADGEQRLLVYEYMPLGSLEDHLHDLPPDKEPLDWNTRMKIA
AGAAKGLEYLHDKAQPPVIYRDFKSSNILLGDDFHPKLSDFGLAKLGPVGDKSHVSTRVMGTYGYCAPEY
AMTGQLTVKSDVYSFGVVLLELITGRKAIDSTRPHGEQNLVSWARPLFNDRRKLPKMADPGLQGRYPMRG
LYQALAVASMCIQSEAASRPLIADVVTALSYLASQIYDPNAIHASKKAGGDQRSRVSDSGRTLLKNDEAGS
SGHKSDRDDSPREPPPGILNDRERMVAEAKMWGANLREKTRAAASAQGSLDSPTETG*

>*A._americanus*_CDG
MGCFSCFDSKEEVGLNQESGRDDIKREEQPMVAPRINKVSSAADKVKSRNNSVLKKEPSGPKETIPAVHISA
QTFTFRELAAATKNFRPECFLGEGGFGRVYKGRLESTGQAVAVKQLDRNGLQGNREFLVEVLMLSLLHHP
NLVNLIGYCADGDQRLLVYEFMPLGSLEDHLHDLPPEKEPLDWNTRMKIAAGAAKGLEYLHDEANPPVIY
RDFKSSNILLDEGYHPKLSDFGLAKLGPTGDKSHVSTRVMGTYGYCAPEYAMTGQLTVKSDVYSFGVVFL
ELITGRKAIDSTRAHGEQNLVTWARPMFNDRRKLQKLADPRLQGRYPMRGLYQALAVASMCIQEQAATR
PLIADVVTALSYLANQSYDPNSAPTPSLRVTGSRAGLSERNLVKKENGGESEKDDSPKETARILNRDLERER
AVAEAKMWGENWRRANAQGNVDVVNG*

>*D._alata*_CDG
MGCWPCFGSDKSKGEEKKPGGDFRKEGSTAPSVTRVASDKSKSRDGSESKKEASAPKDGNPGHIAAQTFT
FRELAAATKNFRQECLLGEGGFGRVYKGRLESTGQVVAVKQLDRNGLQGNREFLVEVLMLSLLHHPNLVS
LIGYCADGDQRLLVYEFMPLGSLEDHLHDVPSHKEPLDWNTRMKIAAGAARGLEYLHDKANPPVIYRDFK
SSNILLDEGYFPKLSDFGLAKLGPVGDKTHVSTRVMGTYGYCAPEYAMTGQLTLKSDVYSFGVVFLELITG
RKAIDSTRPTGEQNLVAWARPLFKDRRKFPKMADPLLQGHYPMRGLYQALAVAAMCLQEQAATRPLIGD
VVTALSYLASQTYDPNATASQRVGPSTPRSREDRRNLGCVSDSQFVHSPHRNSPDFRQRDLTRGSSRGDSA
SGSGRKGILDEFDRQDSQKDSPIHGTKARDSPKNLNRDLNRELAIAEAKVWGENWRERKRTNTPGSFDGT
DE*

>*A._officinalis*_CDG
MMGCFPCFGSTKGSKIEVNSSQQGGGDLKKEASVAPSSDKSKSQSGSESKNEALVTKEGNATRIAARTFTF
RELAAATQNFRQECLIGEGGFGRVYKGRLEDGQVVAVKQLDRNGLQGNREFLVEVLMLSLLHHPNLVNLI
GYCADGDQRLLVYEFMPLRSLEDHLHDLPSDKEPLDWNTRMKIAAGAAKGLEYLHDKANPPVIYRDLKSS
NILLGEGYHPKLSDFGLAKLGPVGDKTHVSTRVMGTYGYCAPEYAMTGQLTLKSDVYSFGVVLLEIITGRK
AIDSTRPTGEQNLVAWARPLFKDRRKFPKMADPLLQGRYPMRGLYQALAVAAMCLQEQAATRPLIGDVV
TALTYLASQSYDPNAAPPQRNRASLHALRTKEESRGLGGGFDSPRANDSPRQNSPGFRYNRKETLEEPEAH
ESHNDSPKNFGKAVESRRSLKDIDRERAVAEAKLWGENSRERRRSNAAGSYEGTNE*

>*C._nucifera*_CDG
MGCFLCAGEPSKKGKEKDQIPTASEEKSKSNTLSDIKKQSSQDAKEISNNGCEHIAAQTFTFRELAAATKNF
RADCLLGEGGFGRVYKGQLDSINQVVAIKQLDRNGLQGNREFLVEVLMLSLLHHPNLVNLIGYCAEGDQR
LLVYEYMPLGSLEDHLHDPSPDKKRLDWNTRMKIAAGAAKGLEYLHDKASPPVIYRDLKCSNILLGEGYH
PKLSDFGLAKLGPVGDKTHVSTRVMGTYGYCAPEYAMTGQLTLKSDVYSFGVVLLELITGRRAIDNSRAA
GEHNLVAWARPLFKDRRKFSQMADPMLQGQYPVRGLYQALAVAAMCVQEQPTMRPLIADVVTALTYLA
SQTYNPEAQSNQNASRLTAPGTPPRTRRDSEKRPNGGSERDQLRGLK*

>*Z._officinalis*_CDG
MGCFPCFESQRGLALNPRRPKNDVRAEHLHPMVPPHIDRVSSLANSGNAGSNSGTKKEVQSVKDLSGVTIS
AQTFTYRELAAATKNFRDECFLGEGGFGCVYKGQLESTGQIVAVKQLNRNGLQGNREFLVEVLMLSLLHH
QNLVNLIGYCADGEQRLLVYEFMPLGSLEDHLHDLPHDNESLDWNTRMKIAAGAAKGLEYLHDKAIPPVI
YRDFKSSNILLGEGYHPKLSDFGLAKLGPTGDKSHVSTRVMGTYGYCAPEYAMTGQLTVKSDVYSFGVVL
LELITGRKAIDSTKPHGEENLIAWAQPMFNDRRKLPRLADPRLQGRFPMRGLYQALAVASMCIQEQAASRP
LIADVVTALSYLANQAYDLNASPTSSSRSGGDQSERKVRSGGRMIVRNEEGGGSGQKLEPEGSERQDSPRE
VVGLLKKDFDRERAVAEAKMWGENWREKMRANSHDTSNANS*

C. nucifera CDG	100.00	73.44	75.13	75.00	69.65	73.28	69.40
A. officinalis CDG	73.44	100.00	76.63	77.99	70.55	75.85	72.36
***S. polyrhiza* CDG**	**75.13**	**76.63**	**100.00**	**80.08**	**67.26**	**73.61**	**69.51**
D. alata CDG	75.00	77.99	80.08	100.00	68.20	74.94	69.44
S. cereale CDG	69.65	70.55	67.26	68.20	100.00	70.39	71.52
A. americanus CDG	73.28	75.85	73.61	74.94	70.39	100.00	75.22
Z. officinalis CDG	69.40	72.36	69.51	69.44	71.52	75.22	100.00
